# Isolation of a Lewis acid-base stabilized stannanone

**DOI:** 10.1039/d5sc06549f

**Published:** 2025-10-07

**Authors:** Mike Jörges, Daniel Knyszek, Manoj Kumar, Varre S. V. S. N. Swamy, Viktoria H. Gessner

**Affiliations:** a Faculty of Chemistry and Biochemistry, Ruhr-University Bochum, Universitätsstrasse 150 44801 Bochum Germany viktoria.gessner@rub.de

## Abstract

A diylide-substituted stannanone, stabilized by the Lewis acid SnCl_2_ (formally written as Y_2_Sn=O → SnCl_2_) has been successfully isolated and characterized by spectroscopic, crystallographic and computational methods. The nature of the ylide substituent proved critical for its successful isolation. While oxidation of a stannylene with a cyano-substituted ylide yielded only a dimeric stannoxane, incorporation of a thiophosphinoyl moiety provided sufficient steric bulk and additional stabilization through P=S coordination, enabling the isolation of the monomeric stannanone. Computational studies revealed a strongly polarized Sn–O bond with negligible π-contribution to the bonding interaction and high opposing charges, resulting in a short Sn–O linkage but a high reactivity toward bond cleavage. This study highlights the challenge associated with stabilizing formal multiple bonds with the heavier atoms and underscores the importance of substituent design in achieving such stabilization.

## Abstract 2

A diylide-substituted Lewis acid-base stabilized stannanone was successfully isolated. Experimental and computational studies revealed a short, but highly polarized Sn–O bond with negligible π-contribution and high reactivity toward bond cleavage.

## Graphical abstract

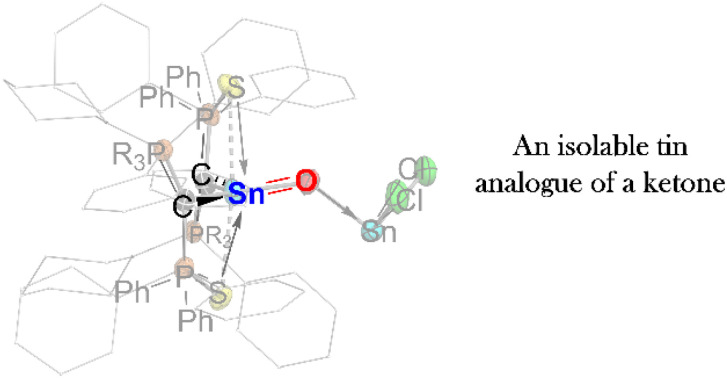

## Introduction

The carbonyl moiety is one of the most ubiquitous functional groups in both nature and organic synthesis. Its highly stable yet polar C=O linkage gives rise to a vast array of chemical reactivity and enables numerous transformations, including many large-scale processes. By contrast, much less is known about the corresponding compounds of the heavier group 14 elements owing to the pronounced lability of E = O bonds with E = Si–Pb.^1–3^ The weaker π overlap, combined with the greater electronegativity difference between oxygen and the tetrel atom imparts a pronounced ylidic character to the E^+^–O^−^ linkage, leading to an enhanced reactivity and reduced stability relative to singly bonded analogues.^4^ Therefore, heavier carbonyls tend to undergo oligo- and polymerization reactions, as exemplified by the differences between carbon and silicon dioxide, or by the polymer chemistry of silicones.^5^ Consequently, heavier carbonyls have long been considered elusive species.^6–9^

**Fig. 1:**
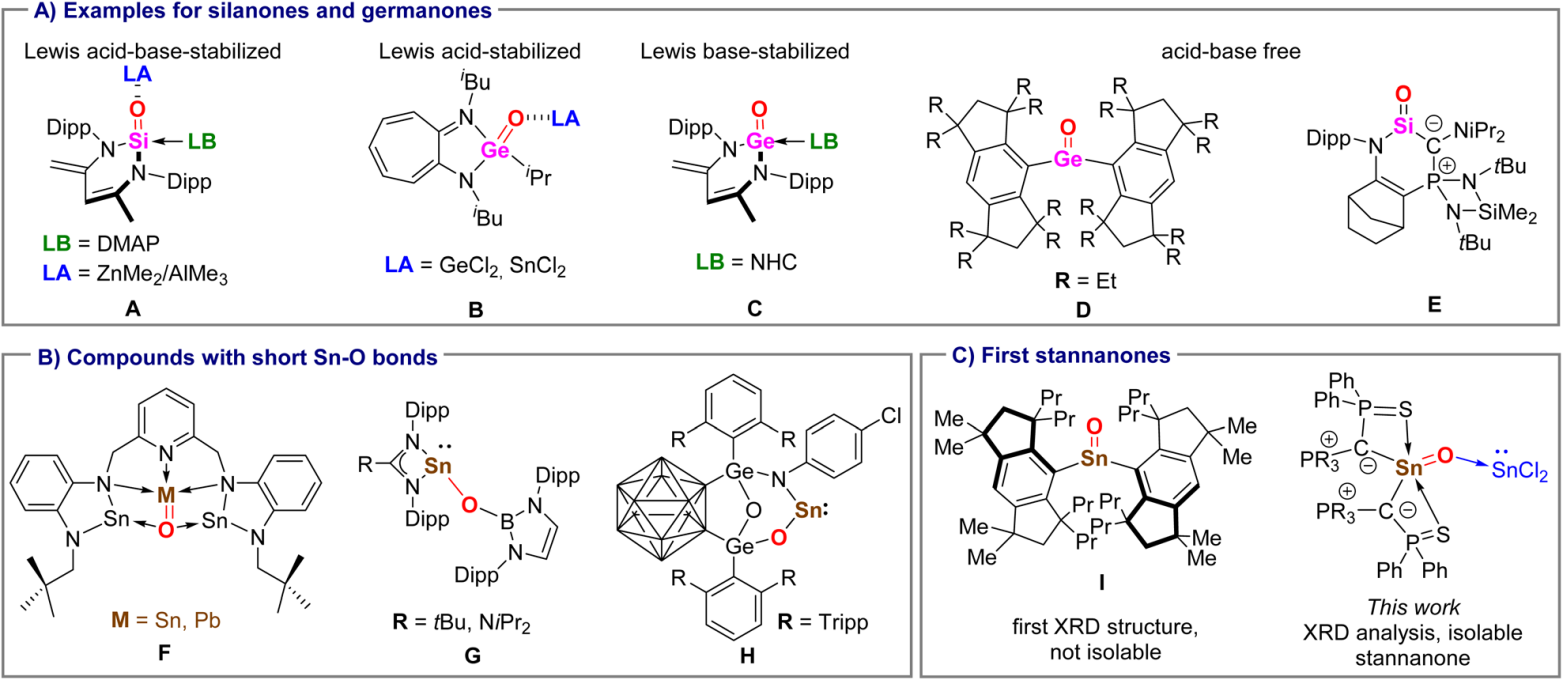
(A) Examples of isolated acid/base and three-coordinate silanones and germanones (Dipp = 2,6-diisopropylphenyl, DMAP = 4-dimethylaminopyridine), (B) reported compounds with short Sn=O bonds and structures of reported stannanones. (C) Structures of the first stannanone and Lewis acid–base stabilized stannanone reported in this work.

The rich chemistry of the C=O moiety in organic compounds has spurred intense research efforts in recent years to isolate heavier carbonyl analogues and to elucidate their structural and chemical properties. After the first report of a donor–acceptor stabilized silacarbonyl compound (A, Fig. 1) by Driess in 2007, many examples of isolable acid/base coordinate silanones have been reported.^10–13^ Using the same approach, also isolable germanones could be generated,^14^ for instance Nagendran's amino-troponiminate-supported Lewis acid-base stabilized germanones B or Driess's Lewis base stabilized system C.^14,15^ Donor/acceptor free, three-coordinate silanones and germanones long remained inaccessible but could be isolated through kinetic stabilization by introduction of highly sterically demanding substituents such as in Tamao's germanone D,^16^ or through electronic stabilization by employing strong donor substituents as employed in the first room temperature stable silanone E reported by Kato *et. al.*^17^

Although many additional examples of isolable silanones^18–24^ and germanones^25,26^ have been reported until today, the situation is much different for their heavier analogues. Owing to the increasing electronegativity difference and weaker π-overlap when descending the group, examples of isolated compounds with Sn–O or Pb–O multiple bonds are extremely rare. In 2008, the Hahn group reported the heavier carbon monoxide congeners F, featuring a formal E = O double bond with tin and lead.^27^ However, the reported Sn=O bond length of 2.079 (2) Å was only slightly shorter than the Sn–O single bonds in F, arguing for only a weak π-interaction. Until today, this compound remains the only isolated species with a partial Sn=O multiple bond character. In the preparation phase of this manuscript, Matsuo and coworkers reported the structural characterization of the three-coordinate stannanone I with a short Sn=O bond of 1.864 (6) Å but failed to isolate the compound due to its high reactivity.^28^ Further attempts to access stannanones led to structures with Sn–O single bonds or completely different reaction products through intramolecular rearrangement reactions.^29^ For example, Aldridge and Mo *et al.* independently reported the synthesis of oxylstannylenes G (ref. 30) and H,^31^ respectively, through oxidation of stannylenes with nitrous oxide followed by migration of one substituent. Notably, both compounds featured shorter Sn–O bond lengths (approx. 2.027 Å) compared to F.

In recent years, our group has showcased the potential of ylide groups in isolating reactive main group compounds^32^ and promoting small molecule activation.^33,34^ Their strong donor ability has proven highly effective in stabilizing low-valent and cationic species.^35–38^ Building on this insight, we hypothesized that ylide-functionalization could enable the stabilization of a stannanone. Here, we report on the isolation of a diylidylstannanone stabilized by a thiophosphinoyl-tethered ylide and SnCl_2_ as additional Lewis acid.

## Results and discussion

**Fig. 2:**
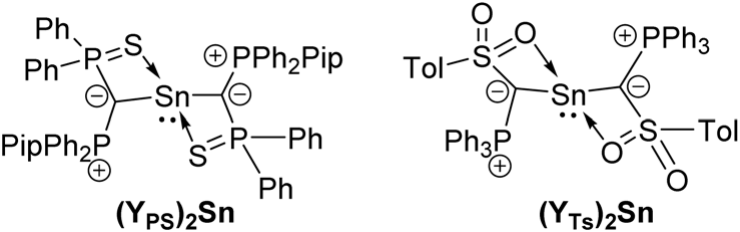
Diylidylstannanones tested in the synthesis of the corresponding stannanones (Pip = piperidyl).

We began our studies with the oxidation of the recently reported diylidylstannylenes (Y_PS_)_2_Sn and (Y_TS_)_2_Sn (Fig. 2).^33,39^ However, treatment of the compounds with nitrous oxide repeatedly resulted in the formation of complex product mixtures including considerable amounts of free ylide. Therefore, we turned our attention towards monoylidylstannylenes of the type YSnR with a variable R substituent. The variable R group appeared advantageous for tuning ligand properties to enable a sufficient stabilization of a stannanone.

**Scheme 1:**
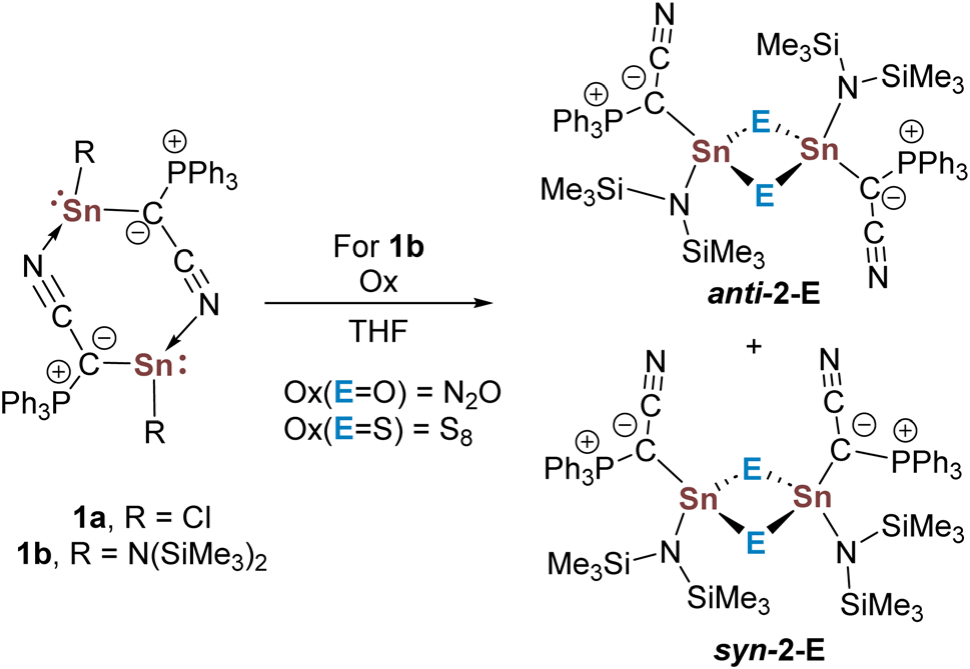
Formation of the cylic heavier stannoxane derivatives *syn*-2-E through oxidation of the dimeric stannylenes 1.

**Fig. 3:**
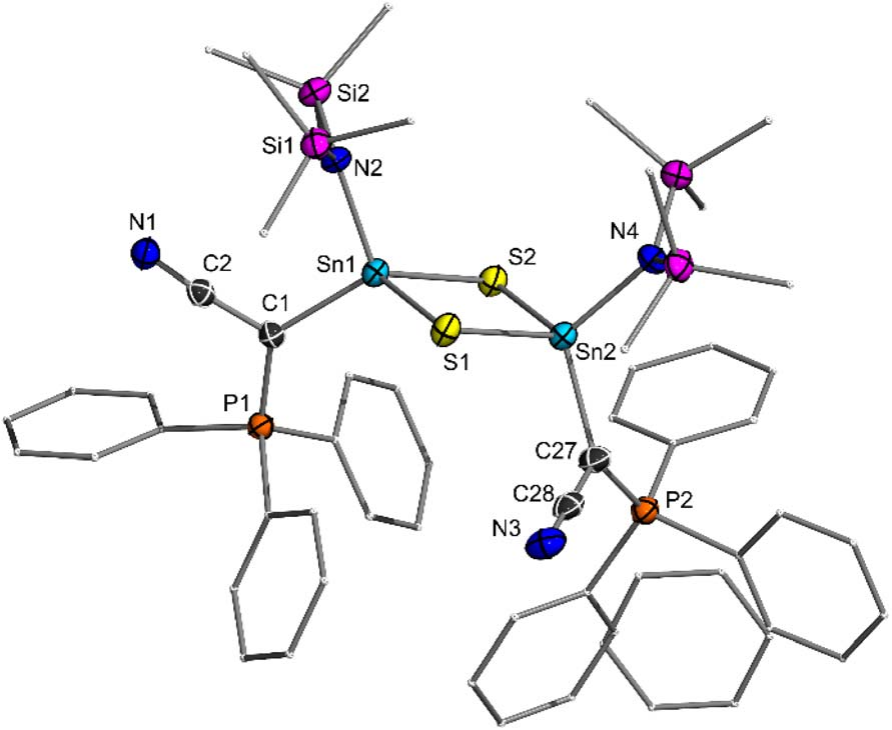
Molecular structure of *syn*-2-S, with thermal ellipsoids drawn at the 50% probability level. Hydrogen atoms omitted for clarity. Important bond lengths and angles as well as crystallographic details are given in the SI.

We initially started oxidation experiments using the ylidylstannylenes Y_CN_SnR [R=Cl (1a), N(SiMe_3_)_2_ (1b)] containing a cyano-substituted ylide (Scheme 1).^40^ We quickly had to rule out the chlorostannylene due to its poor solubility, which hindered further reactions. In contrast, the amido system 1b readily reacted with N_2_O to form a new product as confirmed by ^31^P{^1^H} NMR spectroscopy, which we could identify as the cyclic stannoxane 2-O (Scheme 1). A similar reactivity was observed with sulfur,^41^ for which the corresponding heavier carbonyl analogues are usually more easily accessible.^42–48^ Crystallization and single-crystal X-ray diffraction (XRD) analysis of the products 2-E revealed dimeric structures in the solid-state (Fig. 3, SI) with Sn–O (average: 2.002 (5) Å) and Sn–S bond lengths (average: 2.423 (1) Å) in the range of typical single bonds.^49^ For both the oxygen and sulfur compound, the isomer *syn*-2-E with both ylide groups facing the same side of the central four-membered ring was observed.

Density functional theory (DFT) calculations on the B86/def2-TZVPP level of theory confirmed the preferred formation of the *syn*-isomers, which are energetically favored by 5.5 kcal mol^−1^ in the case of *syn*-2-O and by 6.2 kcal mol^−1^ in the case of *syn*-2-S. In addition, the dimers are clearly favored over the monomeric heavier ketones by more than 50 kcal mol^−1^ in each case (see SI for details).

**Scheme 2:**
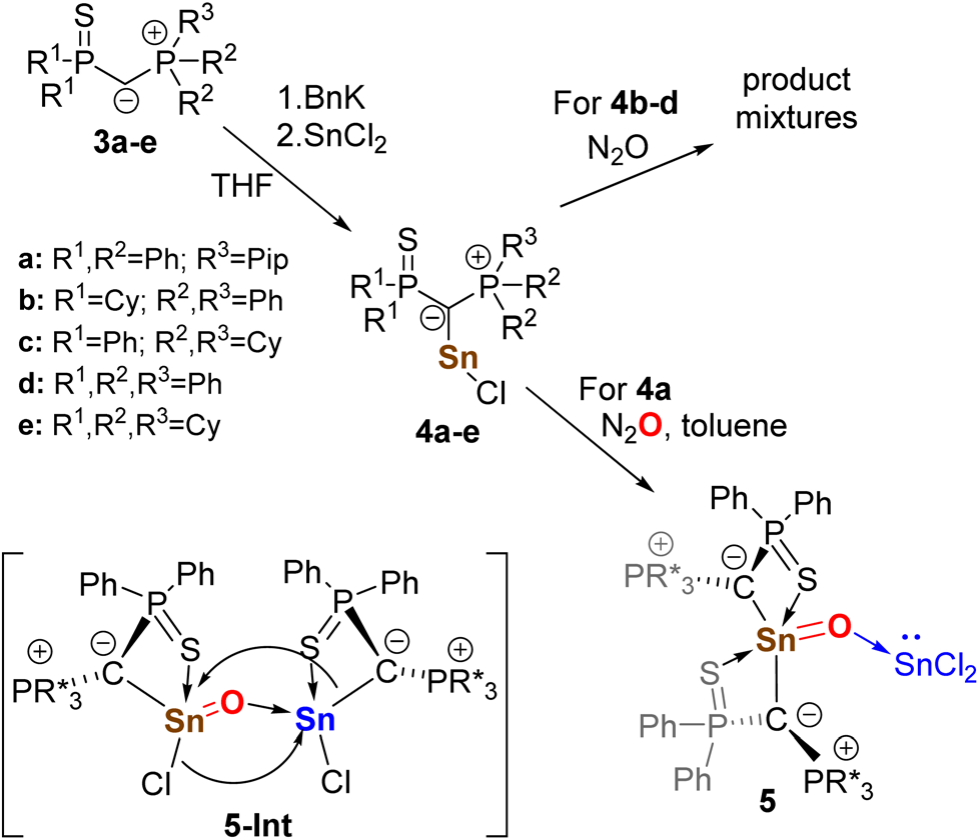
Synthesis of chlorostannylenes 4 and oxidation with nitrous oxide to the Lewis acid base-stabilized stannanone 5 (Pip = piperidyl).

To prevent the dimerization of the formed stannanone to the cyclic stannoxane, we turned our attention to an ylide system with increased steric bulk. The thiophosphinoyl substituted ligands 3a and 3d previously reported by our group appeared to be suitable candidates owing to their steric demand and ability to readily form mono- and diylide-substituted stannylenes.^39,50,51^ Recognizing that solubility could remain a challenge for the isolated stannylenes, we sought to develop similar ylide systems with enhanced solubility. Therefore, we also targeted the synthesis of cyclohexyl-substituted ylides 3b-c and 3e (Scheme 2). Although the synthesis of the fully cyclohexyl-substituted ylide 3e proceeded as planned, its reduced acidity prevented deprotonation to the metalated ylide, even when using strong metal bases such as benzyl potassium. In contrast, the unsymmetrical ylides 3b and 3c and their metalated congeners were readily accessible,^52^ and allowed the selective synthesis of stannylenes 4b and 4c. Attempts to oxidize these complexes with N_2_O to obtain the desired heavier acyl chlorides initially appeared successful. However, the obtained products rapidly decomposed *via* disproportionation, consistently yielding products containing tin(iv) chloride species (see below).

Since isolating a stannanone using ylides 3b–3e proved unsuccessful, we shifted our focus to ylide 3a and its corresponding stannylene 4a. Initial experiments showed the successful synthesis of a new compound, whose selectivity, however, proved to be highly sensitive to the reaction conditions. To circumvent solubility issues with 4a and prevent decomposition, a precise control of reaction time and choice of solvent system was found to be decisive. The *in situ* formation of 4a*via* deprotonation of 3a with BnK in toluene, followed by reaction with 1 equiv. SnCl_2_, and subsequent addition of THF prior to exposure to N_2_O proved to be the optimal reaction conditions. In contrast, excess SnCl_2_ led to the formation of SnCl_3_ species. Strict anhydrous conditions were critical, as even trace amounts of water in N_2_O prevented any selective product formation. Under the optimal conditions the product crystallized directly from the reaction mixture and could be isolated as colorless solid in yields between 85 and 93%. XRD analysis revealed the product to be the diylide-substituted stannanone 5 stabilized by SnCl_2_ as Lewis acid (Fig. 3). The formation of 5 indicates that the oxidation of 4a proceeds through the stannylene-stabilized chlorostannanone intermediate 5-Int, which undergoes exchange of an ylide and chloride ligand to yield 5. Consequently, stannanone 5 cannot be accessed through the oxidation of (Y_PS_)_2_Sn (Fig. 2) in the presence of SnCl_2_. All oxidation attempts starting from the diylidylstannylene have proven to be highly unselective.

The stannanone proved to be stable at room temperature under an inert atmosphere for extended periods (at least 6 months) and also showed extended stability in non-protic solvents. Even when a solution of 5 in CDCl_2_ was heated for three days at 70 °C, no decomposition was observed. Nonetheless, attempts to scale up the reaction from mg scale in a J-Young NMR tube to 20 mL Schlenk tubes remained unsuccessful.

**Fig. 4:**
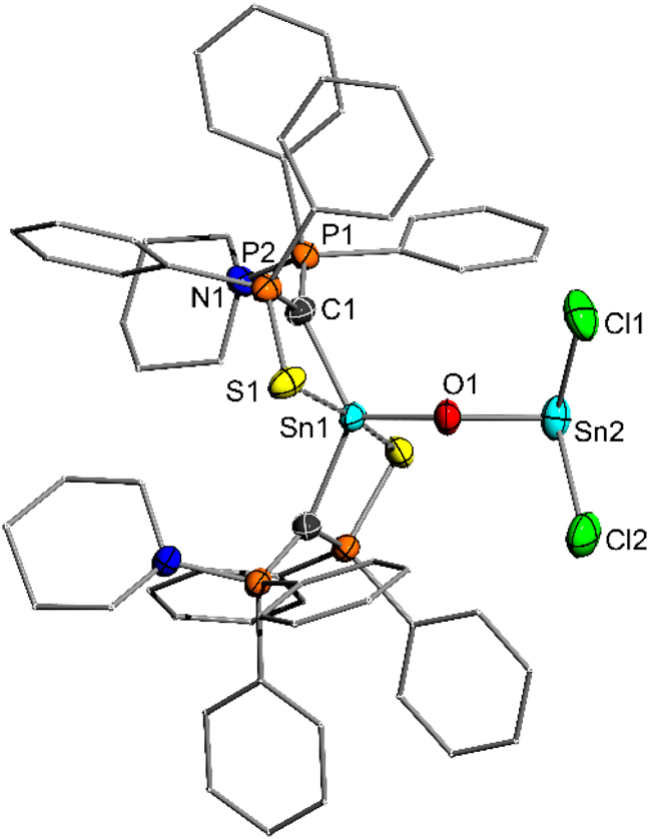
Molecular structure of 5 with thermal ellipsoids drawn at 50% probability level. Hydrogen atoms are omitted for clarity. Important bond lengths [Å] and angles [°]: Sn1–O1 1.955 (4), O1–Sn2 1.984 (4), C1–Sn1 2.126 (3), C1–P1 1.700 (3), C1–P2 1.723 (3), Sn1–O1–Sn2 134.9 (2), P–C–P 128.5 (2).

5 is characterized by two doublets in the ^31^P{^1^H} NMR spectrum at 29.8 (Ph_2_PS) and 41.3 ppm (Ph_2_PPip) with a small coupling constant of ^2^*J*_PP_ = 3.5 Hz and the expected signals for the phenyl and piperidyl protons in the ^1^H NMR spectrum. Further analysis in solution was unfortunately hampered by the extremely low solubility in common aprotic solvents. However, the purity of the crystalline compound was unambiguously confirmed by elemental analysis. In the solid-state structure (Fig. 4), the Sn1–O1 bond length of 1.954 (3) Å is slightly shorter than the Sn2–O1 bond (1.984 (4) Å), suggesting that 5 is more accurately described as a SnCl_2_-stabilized stannanone, rather than as a heavier analogue of phosgene (form 5c, Fig. 4e). Importantly, both bonds are clearly shorter than the Sn–O bonds in compounds F–H, but longer than in Matsuo's Lewis acid base-free stannanone I (1.89 Å). The Sn1–O1–Sn2 angle in compound 5 of 134.9 (2)° is in line with analogous compounds, such as the respective Ge–O–Sn angle of 136.10 (3)° in the SnCl_2_-stabilized germanone B. Interestingly, the Sn–C distance (2.126 (3) Å) in the stannanone is shortened compared to the chlorostanylene precursor 4a (2.202 (2) Å) as a consequence of the oxidation of the tin center and an increased shift of electron density from the ylide ligand to tin. As a result, the electrostatic interactions in the ylide backbone are weakened, as expressed in a slight elongation of the P–C bonds (*e.g.* P1–C1: from 1.683 (2) Å in 4a to 1.700 (3) Å in 5).

**Fig. 5:**
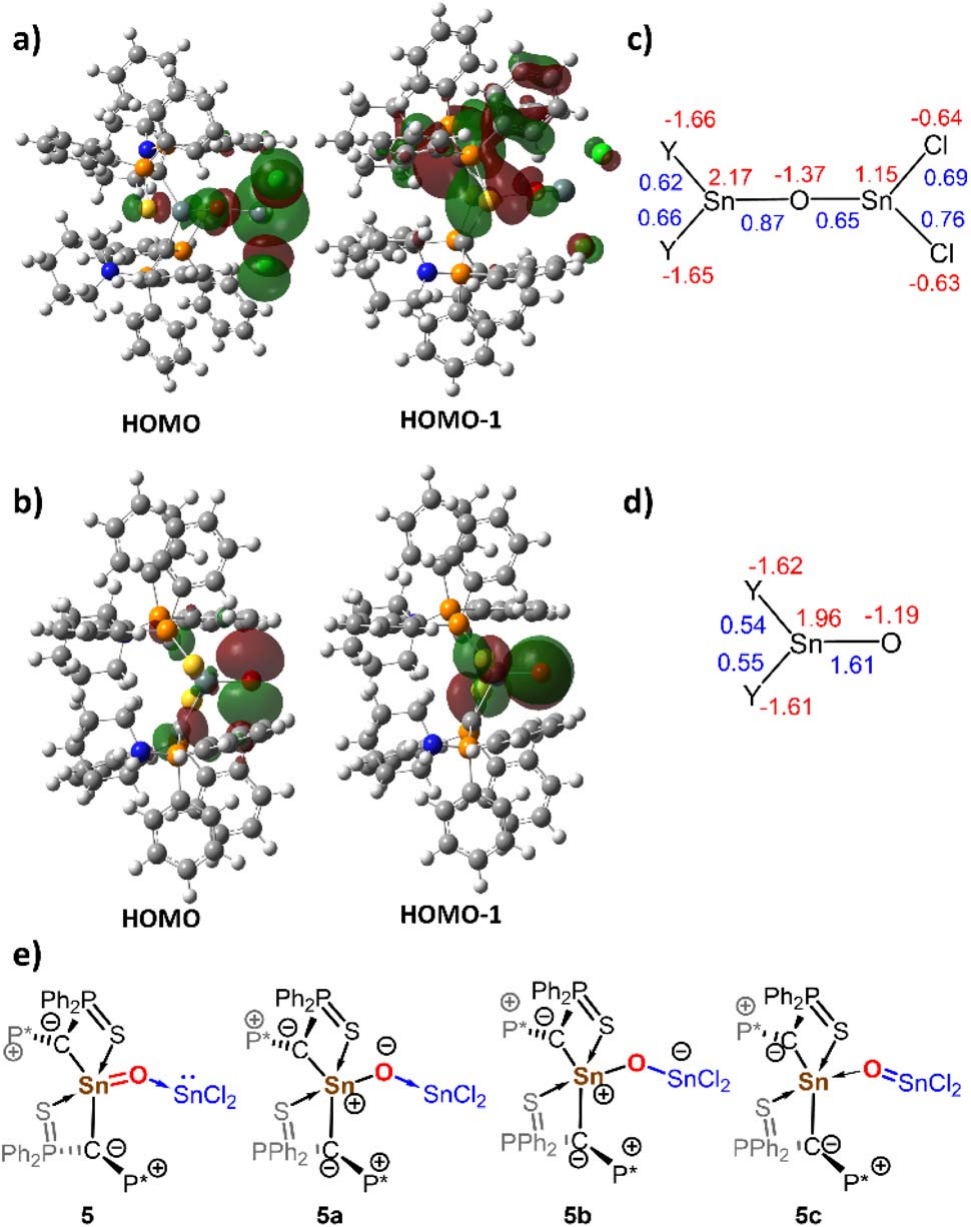
Display of the Kohn–Sham orbitals in (a) 5 and in (b) 5′ as well as calculated NPA charges and Mayer bond indices in (c) 5 and (d) 5′. Possible Lewis structures of 5. (e) Canonical structures of 5.

To gain insights into the bonding situation, DFT calculations on the BP86/def2-TZVPP//BP86/def2-svp level of theory were performed for 5 and the SnCl_2_-free stannanone 5′ (see the ESI for details). The optimized structure of 5 closely matches the experimental values obtained from single crystal XRD analysis (Fig. 4 and Table 1) and confirms the shorter Sn–O bond to the ylide-substituted tin centre. The Sn1–O bond only shortens slightly in the Lewis acid-free analogue 5′, a trend also observed for Matsuo's stannanone I. Analysis of the frontier molecular orbitals shows that the highest occupied molecular orbital (HOMO) of 5 is primarily localized on the SnCl_2_ moiety, corresponding to the lone pair at the tin centre and confirming its role as a Lewis acid (Fig. 5). HOMO-1 and HOMO-2 are localized on the ylidic carbon atoms. No π-symmetric molecular orbital indicative for a multiple bond between tin and oxygen was found.

**Table 1:** Calculated bond lengths, bond angles, Mayer bond indices (MBI) and the ellipticity of the electron density (*ε*) and the charge density (*ρ*) at the bond critical points (BCPs) of compounds 5 and 5′, I, and I–SnCl_2_ (B86/def2tzvpp)

	5	5′	I-SnCl_2_	I
d(Sn1–O) [Å]	1.96	1.89	1.93	1.89
d(Sn2–O) [Å]	2.10	—	2.14	—
Sn1–O–Sn2 [°]	126.3	—	140.2	—
*ρ*(BCP Sn1–O)	0.12	0.14	0.13	0.14
*ρ*(BCP Sn2–O)	0.08	—	0.08	—
*ε*(BCP Sn1–O)	0.03	0.09	0.03	0.01
*ε*(BCP Sn2–O)	0.03	—	0.02	—
MBI (Sn1–O)	0.87	1.61	0.94	1.52

Natural bond orbital (NBO) analysis yielded a highly ionic picture of the bonding situation with predominantly electrostatic interactions within the Sn1–O–SnCl_2_ scaffold. Orbital interactions within this linkage are exclusively represented in the second order perturbation theory analysis, which yielded additional stabilizing O → Sn donor–acceptor interactions from the oxygen centre to both tin centres. In contrast, intrinsic bond orbital (IBO) analysis showed σ-bond orbitals for the Sn–O bonds, but no π-bonding orbitals. Accordingly, low Mayer bond indices (MBI) of 0.87 for the Sn1–O1 bond and 0.65 for Sn2–O1 bond are obtained. Additionally, the Sn–O–Sn linkage features strongly opposing charges, with a higher positive charge at Sn1 (*q* = 2.18) than at Sn2 centre (*q* = 1.16), supporting the oxidation of the Sn1 center and the interpretation of 5 as an SnCl_2_ stabilized stannanone, heavier ylide 5a or as zwitterionic stannoxane represented by the Lewis structure 5b (Fig. 5e). A high bond polarization is also found in the SnCl_2_-free stannanone 5′. Despite a significantly increased MBI for the Sn=O bond of 1.61, the IBO analysis of 5′ also shows no π-bonding orbital and both the HOMO and HOMO-1 are localized on the oxygen atom.

**Fig. 6:**
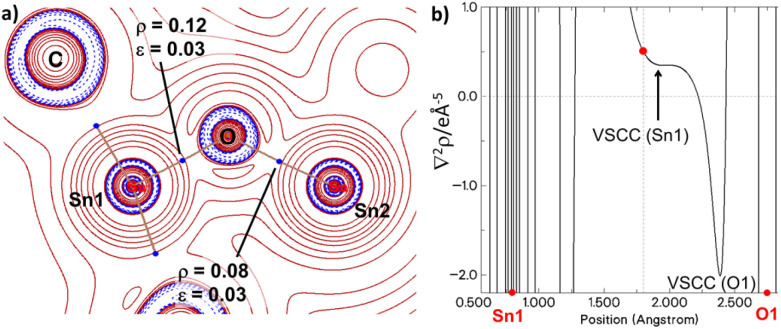
Topological analysis of 5. (a) Contour plot of the Laplacian of electron density in 5. BCPs of the Sn1–O–Sn2 linkage are shown as blue dots with the respective values for the charge density *ρ* and ellipticity ε of the electron density. (b) 1D Laplacian profiles of the Sn1–O1 bond-path. BCPs are shown as red dots.

Next, we also examined the electronic structure by quantum theory of atoms in molecules (QTAIM) calculations,^53^ which revealed bond critical points (BCPs) for the Sn1–O linkage with low ellipticity values of *ε* = 0.03 for 5 and *ε* = 0.09 for 5′, indicative of single bonds (Fig. 6). Interestingly, analysis of the Laplacian profile along both Sn–O bond paths in 5 shows only one pronounced valence-shell charge concentration (VSCC) minimum close to the electronegative oxygen atom and a mere shoulder for the Sn VSCC. Both VSCCs reside in the atom basin of the oxygen atom, indicating a dative O → Sn interaction for both Sn–O bonds as suggested by the NBO analysis.^54^

To examine whether this bonding situation is unique to our diylidylstannanone, we conducted comparative electronic structure analyses of Matsuo's stannanone I (including its Lewis acid adduct with SnCl_2_) and the Lewis acid stabilized germanone B reported by Nagendran *et al.* (see SI).^15^ For compound I, a higher MBI of 1.52 was calculated; however, this value reduces to 0.94 upon SnCl_2_ coordination, mirroring the trend observed for compound 5 and 5′. Topological analysis of I further reveals bond parameters comparable to those of 5′, albeit with an even lower ellipticity for the Sn1–O1 bond of *ε* = 0.01. Overall, the calculations demonstrate that the π-contribution to the bonding between the tin and the oxygen centre in the stannanones is negligible. The Sn–O bond is best described by an ylidic interaction with a polar single bond which shortens relative to standard Sn–O single bonds due to additional electrostatic interactions (structure 5a and 5b, Fig. 4).

In line with the highly polar Sn–O bond, stannanone 5 displays pronounced sensitivity toward trace amounts of water. Initial screening reactions towards small molecules, which at first appeared selective based on NMR analysis, were later attributed to unintended side reactions with residual water present in the starting materials. Careful drying of the reactants eliminated these side reactions and completely suppressed the reactivity. In the absence of water, 5 was found to be stable in the solid state for several months without any detectable decomposition.

**Scheme 3:**
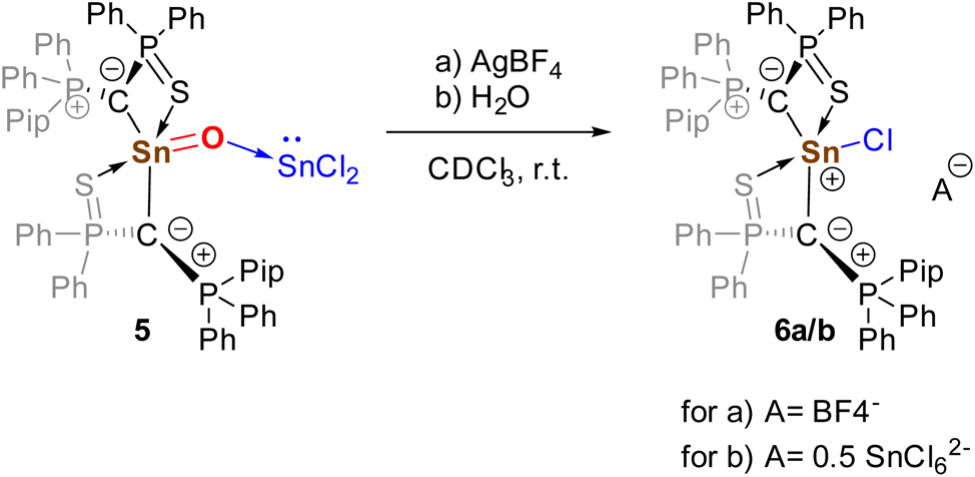
Reaction of stannanone 5 with AgBf_4_ and H_2_O (Pip = piperidyl).

**Fig. 7:**
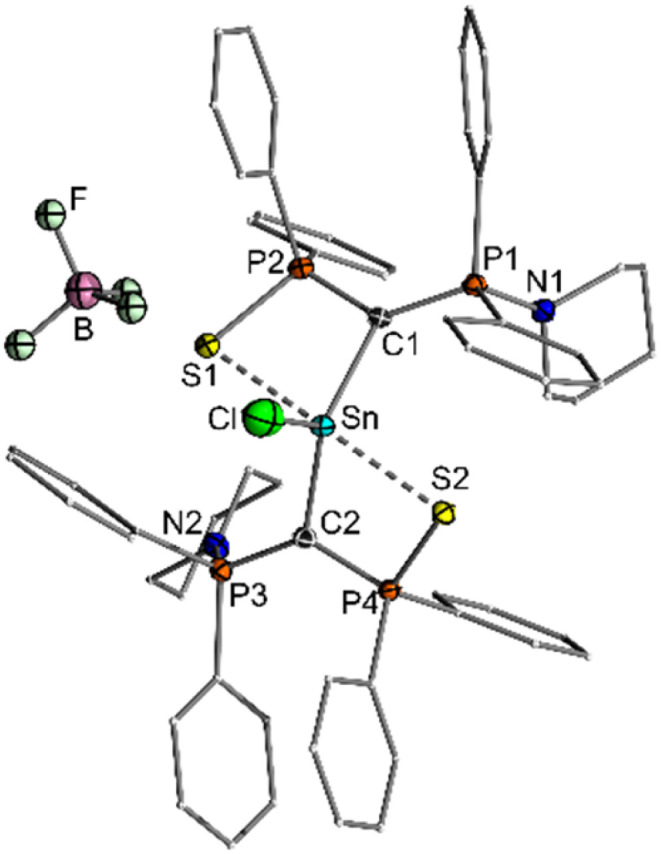
Molecular structure of the BF_4_^−^ salt of 6. Thermal ellipsoids drawn at 50% probability level. Hydrogen atoms omitted for clarity. Important bond lengths and angles as well as crystallographic details are given in the SI.

In general, 5 was found to be highly reactive towards various substrates (*e.g.* CO, CO_2_, S_8_, CS_2_), but usually reacted in an uncontrollable fashion producing multiple products. Attempts to remove (addition of bases such as NH_3_, dmap) or exchange the SnCl_2_ Lewis acid (*e.g.* with GeCl_2_, ZnF_2_, B(C_6_F_6_)_3_) always resulted in decomposition. The only transformation, from which a clean product could be isolated, was achieved upon treatment with AgBF_4_ as halide abstraction reagent. This reaction led to the formation of the bisylide-substituted tin chloride cation 6, whose BF_4_ salt was generated with high selectivity but could not be isolated in pure form due to Sn–C bond cleavage occurring during workup (Scheme 3 and Fig. 7). Interestingly, 6 was also formed in many screening reactions, in which water was present, and could be crystallized as its SnCl_6_^2−^ salt. For example, the reaction proceeded highly selectively and rapidly with benzaldehyde in CDCl_3_. In addition to compound 6, ylide-substitute SnCl_3_ was also obtained from some of the reaction attempts (see the ESI), further underscoring the tendency of the stannanone to undergo Sn–O bond cleavage. In general, the high sensitivity toward trace amounts of water and the formation of the Sn–O bond cleavage products support the interpretation of the Sn–O bond as a highly polar ylidic bond.

## Conclusions

To conclude, we have synthesized the first isolable monomeric Lewis acid base-stabilized stannanone formed through an oxidation-induced rearrangement in an ylide-substituted chlorostannylene. Although the stannanone exhibits a short Sn–O bond length of 1.95 Å, calculational studies revealed that there is negligible π-contribution to the bonding. Overall, the Sn–O bond was found to be highly polar, with a significant electrostatic component that accounts for its shortened bond length. As a results of this bonding situation, the stannanone is extremely sensitive to trace amounts of water and often reacts an uncontrollable fashion. Experimental observations indicate that the Sn–O bond undergoes facile cleavage, as evidenced by the formation of a tin cation. These findings highlight the challenges associated with stabilizing compounds of heavier elements featuring multiple bonds, especially when such bonds involve elements from different periods of the periodic table with markedly different properties.

## Author contributions

V. H. G. designed and oversaw the project. M. J. planned the study and carried out most of the synthetic work, analysed the spectroscopic data. D. K. performed all computational studies. M. K. synthesized compound 2-O and helped with crystal refinement of 2-S. V. S. V. S. N. S. synthesized compound 2-S. The manuscript was written by M.J. and D. K. and finalized by V. H. G.

## Conflicts of interest

There are no conflicts to declare.

## Supplementary Material

SC-OLF-D5SC06549F-s001

SC-OLF-D5SC06549F-s002

## Data Availability

CCDC 2481150 (4a-SnCl_3_), 2481151 (4c+N_2_O), 2481152 (6-SnCl_6_), 2481153 (6-BF_4_), 2481154 (2-S) and 2481155 (5) contain the supplementary crystallographic data for this paper.^55*a*–*f*^ The data that support the findings of this study are available in the supporting information (SI). Supplementary information: this includes experimental procedures, NMR and IR spectra as well as crystallographic and computational details. See DOI: https://doi.org/10.1039/d5sc06549f.
