# Supplementary material for: Isolation of a Lewis acid-base stabilized stannanone
Source: Chem Sci. 2025 Oct 7;16(44):21087–93. doi: 10.1039/d5sc06549f (PMC12519236; doi:10.1039/d5sc06549f)
Supplement: SC-016-D5SC06549F-s001 [file SC-016-D5SC06549F-s001.pdf]

Supplementary Information (SI) for Chemical Science.
This journal is © The Royal Society of Chemistry 2025

Isolation of a Lewis acid-base stabilized stannanone

Mike Jörges,[a] Daniel Knyszek,[a] Manoj Kumar,[a] Varre S. V. S. N. Swamy,[a] Viktoria H. Gessner,*[a]

Ruhr University Bochum, Faculty of Chemistry and Biochemistry, Chair of Inorganic Chemistry II,
Universitätsstrasse 150, 44780 Bochum, Germany
E-mail: viktoria.gessner@rub.de

Table of Contents

1. Experimental Details 2
1.1. General Experimental Information 2
1.2 Synthesis of compounds 2 3
1.3 Synthesis of compounds 3 4
1.4 Synthesis of compounds 4 7
1.5 Synthesis of compound 5 9
1.6 Synthesis of compounds 6 10
1.7 List of substrates tested in reactivity studies with stannanone 5 11
2. NMR spectra of all compounds 12
2.1 NMR spectra of compounds 2 12
2.2 NMR spectra of compounds 3 16
2.3 NMR spectra of compounds 4 24
2.4 NMR spectra of compounds 5 31
2.5 NMR spectra of compounds 6 34
3. Crystal structure determination. 36
3.1 General information 36
3.2 ORTEP plots of all crystal structures 40
4. DFT 47
4.1 General remarks 47
4.2 Molecular Orbitals 48
4.3 QTAIM bonding Analysis 49
4.4 NBO Analysis 50
4.6 Coordinates of optimized structures 51
5. References 65

1. Experimental Details

1.1. General Experimental Information

All experiments (if not stated otherwise) were carried out under a dry, oxygen-free argon atmosphere using standard Schlenk techniques. Argon (99.999%) was purchased from Air Liquide. Solvents and chemicals: Involved solvents were dried using a MBraun SPS **7** (THF, toluene, diethyl ether, n-hexane, n-pentane, acetonitrile) or dried in accordance with standard procedures and stored under an argon atmosphere over 3 Å or 4 Å molecular sieves. Reagents were purchased from Sigma-Aldrich, ABCR, Acros Organics or TCI Chemicals and used without further purification if not stated otherwise. BnK[1], **1a**, **1b**[2], **3a**,[3] **3b**[4] and **3d**[5] were synthesized following literature procedures. In the attempts to oxidize the stannylenes **4b–4d** to the corresponding stannanones using N_2O, product mixtures were consistently detected in the NMR spectra. Attempts to crystallize individual products repeatedly resulted in unwanted side products. A single crystal obtained from the reaction of **4b** with N_2O (**4b+N$_2$O**) and another from the reaction of **4c** with N_2O (**4c+N$_2$O**) are presented in Chapter 3.

^{1}H, ^{13}C{^{1}H}, ^{77}Se{^{1}H}, ^{31}P{^{1}H} NMR spectra were recorded on Avance-III-400 spectrometers at 22 °C if not stated otherwise. All values of the chemical shift are in ppm regarding the δ-scale. All spin-spin coupling constants (*J*) are printed in Hertz (Hz). To display multiplicities and signal forms correctly the following abbreviations were used: s = singlet, d = doublet, t = triplet m = multiplet, dd = doublet of doublet, br = broad signal. Signal assignment was supported by, HSQC (^{1}H / ^{13}C), HMBC (^{1}H / ^{13}C, ^{1}H / ^{31}P) correlation experiments.

IR spectra in solution were recorded in an argon filled glovebox on a Shimadzu IRSpirit with QATR-S module. Measurement and processing details for individual spectra can be extracted from the corresponding tables in the supporting information.

IR spectra with solvents were recorded on a Thermo Nicolet iS5 FT-IR in transmission mode with a Specac "Omni-cell" with KBr plates and a 0.1 mm spacer at 22 °C

Elemental analyses were performed on an Elementar vario MICRO-cube elemental analyzer.

HRMS-ESI: An LTQ Orbitrap Velos (Thermo Fisher Scientific, Bremen, Germany) was used for direct infusion via a syringe pump. The heated desolvation capillary was set to 200°C and a spray voltage of 1.8 kV was supplied. In the tune file the LTQ Orbitrap was set to the following parameters (R = 30,000; IT = 500 ms; AGC Target = 1,000,000). ESI mass spectra were recorded in the positive ion mode. Therefore, the [M+H] ions are recorded.

For details about the single-crystal Xray diffraction analyses, see chapter 3.

1.2 Synthesis of compounds 2

Synthesis of compound 2-O

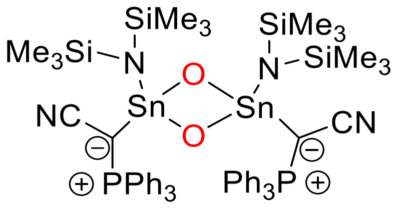

The freshly prepared THF-d_8 (0.5 ml) solution of **1b** (50 mg, 0.041 mmol) in a J-young NMR tube was exposed to nitrous oxide (~ 99.9 %) under inert conditions. Within 5 minutes, the color of the solution changed from yellow to pale yellow. After 20 min. the ^{31}P NMR spectrum of the reaction mixture confirmed the complete consumption of **1b**, and the formation of both **2-anti** (39%) and **2-syn** (61%), along with **Y_{CN}-H**. The removal of all the volatiles from the resulting solution under reduced pressure led to the formation of a pale yellow solid. This solid was washed thrice with the mixture of cold hexane:toluene (3:1). Colorless single crystals for XRD analysis were grown from a saturated C_6D_6 solution of **2-O**. (20 mg, 0.016 mmol, 39%).

^{31}P{^{1}H}-NMR (162.1 MHz, Benzene-d_6): δ = 25.93 (s, <u>P</u>Ph3) ppm.

^{1}H-NMR (400 MHz, Benzene-d_6): δ = 0.29 (s, 18H, SnN(SiC<u>H</u>3)2, 7.20–7.22 (m, 9H,C<u>H</u>$_{PPh3,meta,para}$), 7.62–7.67 (m, 6H, C<u>H</u>$_{PPh3,ortho}$) ppm.

^{13}C{1H}-NMR (100.6 MHz, Benzene-d_6): δ = 4.82 (SnN(Si<u>C</u>H3)2), 127.08 (s, C<u>C</u>N), 129.20 (<u>C</u>$_{PPh3,ipso}$), 129.39 (d, $^3J_{CP}$=12.1 Hz, <u>C</u>H$_{PPh3,meta}$), 132.90 (d, $^4J_{CP}$=2.7 Hz, <u>C</u>H$_{PPh3,para}$), 134.15 (d, $^2J_{CP}$=9.9 Hz, <u>C</u>H$_{PPh3,ortho}$) ppm. P<u>C</u>CN was not observed due to poor solubility.

^{119}Sn{1H}-NMR (149.2 MHz, Benzene-d_6): δ = −46.44 (d, $^2J_{SnP}$=221.9 Hz, [Y_{CN}<u>*Sn*</u>N(SiMe$_3$)$_2$O]$_2$ ppm.

Melting point. 116.6 °C.

Synthesis of compound 2-S

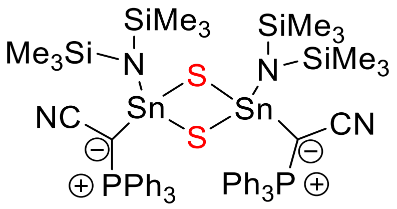

A J-Young NMR tube was charged with **1b** (50 mg, 0.043 mmol, 1 eq.) and elemental sulfur (4.15 mg, 0.129 mmol, 3 eq.). THF-d_8 (0.5 ml) was added to this mixture, and the reaction mixture was shaken for 6h, resulting in a yellow solution. The ^{31}P NMR spectrum of the reaction mixture confirmed the complete consumption of **1b**, and the selective formation of a product. Removal of all the volatiles from the resulting yellow solution under reduced pressure led to the formation of a yellow solid. This solid was washed twice with cold hexane (0.3 ml) and dried to get an analytically pure sample of compound **2-S**, which was

isolated as a pale yellow solid (31.2 mg, 0.025 mmol, 59 % yield). Single crystals for XRD analysis were grown by diffusion of hexane into a C_6D_6 solution of **2-S**.

^{31}P{^{1}H}-NMR (162.1 MHz, THF-*d*$_8$): δ = 26.13 (s, $\underline{P}Ph_3$) ppm.

^{1}H-NMR (400 MHz, THF-*d*$_8$): δ = 0.18 (s, 18H, $SnN(SiC\underline{H}_3)_2$, 7.49–7.54 (m, 9H,$C\underline{H}_{PPh3,meta,para}$), 7.68–7.74 (m, 6H, $C\underline{H}_{PPh3,ortho}$) ppm.

^{13}C{1H}-NMR (100.6 MHz, THF-*d*$_8$): δ [ppm] = 6.19 (s, $SnN(Si\underline{C}H_3)_2$), 127.66 (s, $C\underline{C}N$), 128.56 (s, $\underline{C}_{PPh3,ipso}$), 130.17 (d, $^3J_{CP}$=12.5 Hz, $\underline{C}H_{PPh3,meta}$), 133.81 (d, $^4J_{CP}$=2.9 Hz, $\underline{C}H_{PPh3,para}$), 135.05 (d, $^2J_{CP}$=9.9 Hz, $\underline{C}H_{PPh3,ortho}$) ppm. $P\underline{C}CN$ and $\underline{C}_{PPh3,ipso}$ were not observed due to poor solubility.

^{119}Sn{1H}-NMR (149.2 MHz, THF-*d*$_8$): δ = −16.06 (d, $^2J_{SnP}$=197.9 Hz, $[Y_{CN}\underline{Sn}N(SiMe_3)_2S]_2$ ppm.

Melting point. 105.2 °C

1.3 Synthesis of compounds 3

Synthesis of 3c-Pre

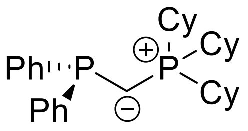

5 g Methyltriphenylphosphonium iodide (11.8 mmol) was added to a round bottom flask, which was then purged with argon. Subsequently, 100 ml THF were added. The resulting suspension was cooled in an ice bath and *^{n}*BuLi (1.6 M in hexane, 7.8 ml, 12.4 mmol) was added dropwise with rigorously stirring until the mixture became clear. Chlorodiphenylphosphine (1.2 ml, 6.5 mmol) was added and the mixture warmed to room temperature. The reaction was stirred at RT for 2 h. After the solvent was removed in vacuo, 100 ml of toluene were added and the mixture again stirred for 2 h. The solution was filtered off. After removing the solvent in vacuo, the resulting solid was ultrasonically agitated with 15 mL of acetonitrile and the mixture was then stirred overnight. Two additional washes with 10 mL of acetonitrile each, followed by drying, yielded the product as a yellow powder (1.8 g, 5.9 mmol, 63 %).

^{1}H-NMR (400 MHz, THF-*d*$_8$): δ = 0.30 (dd, $^2J_{HP}$ = 8.5 + 3.7 Hz, 1H; $PC\mathit{H}P$), 1.11 (qt, $^5J_{HP}$ = 12.8 + 3.4 Hz, 3H; $PCy_3\mathit{H}_{Cy,para}$), 1.20 - 1.33 (m, 6H; $PCy_3\mathit{H}_{Cy,meta}$), 1.43 (qt, $^4J_{HP}$ = 12.6 + 3.8 Hz, 6H; $PCy_3\mathit{H}_{Cy,meta}$), 1.66 (d, $^5J_{HP}$ = 12.9 Hz, 3H; $PCy_3\mathit{H}_{Cy,para}$), 1.76 (dd, $^3J_{HP}$ = 12.3 + 3.8 Hz, 6H; $PCy_3\mathit{H}_{Cy,ortho}$), 1.92 (d, $^3J_{HP}$ = 13.1 Hz, 6H; $PCy_3\mathit{H}_{Cy,ortho}$), 2.19 (qt, $^{2,4}J_{HP}$ = 12.3 + 3.1 Hz, 3H; $PCy_3\mathit{H}_{Cy,ipso}$), 7.03 (t, $^5J_{HP}$ = 7.3 Hz, 2H; $PPh_2\mathit{H}_{Ph,para}$), 7.13 (t, $^3J_{HP}$ = 7.4 Hz, 4H; $PPh_2\mathit{H}_{Ph,ortho}$), 7.49 (t, $^4J_{HP}$ = 7.1 Hz, 4H; $PPh_2\mathit{H}_{Ph,meta}$) ppm.

$^{31}P\{^1H\}$-NMR (162 MHz, THF-d_8): δ = –18.33 (d, $^2J_{PP}$ = 115.3 Hz; PPh_2), 34.03 (d, $^2J_{PP}$ = 115.3 Hz; PCy_3), ppm.

$^{13}C\{^1H\}$-NMR (101 MHz, THF-d_8): δ = –2.39 (dd, $^{1,1}J_{CP}$ = 107.0 + 9.0 Hz, P*C*P), 27.30 (d, $^4J_{CP}$ = 1.5 Hz, $PCy_3C_{Cy,para}$), 28.00 (t, $^{2,4}J_{CP}$ = 2.3 Hz, $PCy_3C_{Cy,ortho}$), 28.19 (d, $^3J_{CP}$ = 11.6 Hz, $PCy_3C_{Cy,meta}$), 34.14 (dd, $^{1,3}J_{CP}$ = 51.1 + 5.6 Hz, $PCy_3C_{Cy,ipso}$), 126.67 (s, $PPh_2C_{Ph,para}$), 128.06 (d, $^2J_{CP}$ = 6.0 Hz, $PPh_2C_{Ph,ortho}$), 132.56 (d, $^3J_{CP}$ = 18.2 Hz, $PPh_2C_{Ph,meta}$), 150.15 (dd, $^{1,3}J_{CP}$ = 11.5 + 7.9 Hz, $PPh_2C_{Ph,ipso}$) ppm.

HRMS-ESI (m/z): $[M+H]^+$ calcd for M = $C_{31}H_{44}P_2$, 479.2991; found, 479.2987.

Melting point. 201.8 °C.

Synthesis of compound 3c

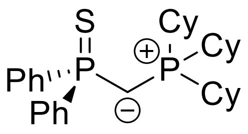

0.5 g of **3c-Pre** (1.04 mmol) and dried sulfur (33 mg, 1.03 mmol) were dissolved in 5 ml THF. The mixture was stirred for 2 h before 75 ml pentane were added. After stirring for 48 h, the liquid was filtered off and the remaining solid dried *in vacuo*. The product was obtained as an off-white powder (380 mg, 0.74 mmol, 71 %).

^1H-NMR (400 MHz, THF-d_8): δ = 0.56 (t, $^{2,2}J_{HP}$ = 2.5 Hz, 1H, PC*H*P), 1.08 (qt, $^{5,7}J_{PH}$ = 13.0 + 3.6 Hz, 3H, $PCy_3H_{Cy,para}$), 1.27 (qt, $^4J_{PH}$ = 12.8 + 3.4 Hz, 6H, $PCy_3H_{Cy,meta}$), 1.45 (qt, $^4J_{PH}$ = 12.6 + 3.8 Hz, 6H, $PCy_3H_{Cy,meta}$), 1.64 (d, $^5J_{PH}$ = 13.0 Hz, 3H, $PCy_3H_{Cy,para}$), 1.74 (dd, $^{3,5}J_{PH}$ = 12.9 + 3.5 Hz, 6H, $PCy_3H_{Cy,ortho}$), 1.92 - 2.01 (m, 6H, $PCy_3H_{Cy,ortho}$), 2.53 (qt, $^{2,4}J_{PH}$ = 12.2 + 2.9 Hz, 3H, $PCy_3H_{Cy,ipso}$), 7.25 (dt, $^{3,5}J_{PH}$ = 6.5 + 2.1 Hz, 6H, $PPh_2H_{Ph,ortho,para}$), 7.84 - 7.97 (m, 4H, $PPh_2SH_{Ph,meta}$), ppm.

$^{31}P\{^1H\}$-NMR (162 MHz, THF-d_8): δ = 34.95 (d, $^2J_{PP}$ = 23.1 Hz, *P*Cy_3), 35.44 (d, $^2J_{PP}$ = 22.9 Hz, *P*Ph_2S) ppm.

$^{13}C\{^1H\}$-NMR (101 MHz, THF-d_8): δ = 1.63 (dd, $^{1,1}J_{CP}$ = 113.5 + 105.9 Hz, P*C*P), 27.17 (d, $^4J_{CP}$ = 1.6 Hz, $PCy_3C_{Cy,para}$), 28.13 (d, $^2J_{CP}$ = 11.8 Hz, $PCy_3C_{Cy,ortho}$), 28.56 (d, $^3J_{CP}$ = 2.8 Hz, $PCy_3C_{Cy,meta}$), 34.67 (dd, $^{1,3}J_{CP}$ = 50.0 + 3.1 Hz, $PCy_3C_{Cy,ipso}$), 128.09 (d, $^2J_{CP}$ = 11.6 Hz, $PPh_2SC_{Ph,ortho}$), 129.51 (d, $^4J_{CP}$ = 2.9 Hz, $PPh_2SC_{Ph,para}$), 131.65 (d, $^3J_{CP}$ = 10.2 Hz, $PPh_2SC_{Ph,meta}$), 145.30 (dd, $^{1,3}J_{CP}$ = 82.6 + 6.3 Hz, $PPh_2SC_{Ph,ipso}$) ppm.

HRMS-ESI (m/z): $[M+H]^+$ calcd for M = $C_{31}H_{44}SP_2$, 511.2712; found, 511.2705.

Synthesis of compound 3e-Pre

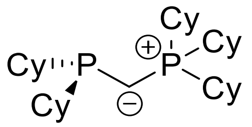

5 g (11.8 mmol) of methyltricyclohexylphosphonium iodide were dissolved in 100 ml THF and cooled to 0 °C. At this temperature, 7.6 ml (11.8 mmol) ^{n}BuLi (1.55 M in hexane) were added slowly and the clear, yellow mixture was stirred for 30 min at 0 °C. 1.4 ml (6.5 mmol) chlorodicyclohexylphosphine were added, stirred for 1 h at 0 °C and slowly warmed to ambient temperature. Removal of the solvent afforded a yellow oil which was dissolved in 200 ml toluene and stirred for 1 h. After filtration and evaporation of the solvent, 50 ml acetonitrile were added and the reaction flask was put in an ultrasonic bath for 30 min. After one hour of stirring and subsquent filtration, a light-yellow solid could be obtained. The light-yellow solid was washed with acetonitrile (3 x 20 ml) and dried *in vacuo*. The product was afforded as a light-yellow solid (2.48 g, 85.5 mmol, 86 %).

^{1}H-NMR (400.3 MHz, C_6D_6): δ = –0.15 (dd, $^2J_{HP}$ = 8.27 + 2.81 Hz, 1H; PC*H*P), 1.05 - 1.23 (br, 9H), 1.27-1.84 (m, 30H), 1.86 - 2.03 (m, 12H), 2.10 - 2.30 (m, 4H) ppm.

^{31}P{^{1}H}-NMR (162.1 MHz, C_6D_6): δ = –10.39 (d, $^2J_{PP}$ = 105.2 Hz; *P*Cy_2), 30.38 (d, $^2J_{PP}$ = 105.2 Hz; *P*Cy_3) ppm.

^{13}C{^{1}H}-NMR (100.7 MHz, C_6D_6): δ = –8.97 (dd, $^{1,1}J_{CP}$ = 111.0, 20.1 Hz; P*C*P), 26.77 (s; PCy_3*C*$H_{Ph,\textit{para}}$), 27.53 (s,), 27.60 - 27.81 (m), 28.57 (d, $^4J_{CP}$ = 2.8 Hz; PCy_2*C*$H_{Ph,\textit{para}}$), 28.60 (d, $^3J_{CP}$ = 16.1 Hz; PCy_3*C*$H_{Ph,\textit{meta}}$), 30.25 (d, $^3J_{CP}$ = 5.5 Hz; PCy_2*C*$H_{Ph,\textit{meta}}$), 32.40 (d, $^2J_{CP}$ = 18.1 Hz; PCy_2*C*$H_{Ph,\textit{ortho}}$), 34.71 (dd, $^{1,3}J_{CP}$ = 50.6 + 3.6, PCy_3*C*$H_{Ph,\textit{ipso}}$), 38.69 (dd, $^{1,3}J_{CP}$ = 11.1 + 6.5, PCy_2*C*$H_{Ph,\textit{ipso}}$) ppm.

HRMS-ESI (m/z): $[M-H]^+$ calcd for M = $C_{31}H_{56}P_2$, 491.3930; found, 491.3924.

Synthesis of compound 3e

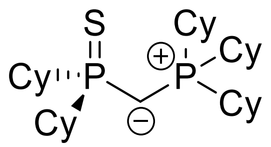

1.00 g (2.04 mmol) of compound **3e-Pre** was dissolved in 10 ml THF. 65 mg (2.04 mmol) elemental sulfur were added and the solution was stirred for 5 min. After evaporation of the solvent the product was afforded as a light-yellow solid (1.05 g, 2.04 mmol, 99 %).

^{1}H-NMR (400.3 MHz, C_6D_6): δ = –0.45 (d, $^2J_{HP}$ = 2.2 Hz, 1H, PC*H*P) ppm.

Due to the multitude of overlapping signals, the signals of the cyclohexyl groups could not be definitively assigned. The signals corresponding to the hydrogen atoms of the cyclohexyl groups appeared between 1.00 and 2.50 ppm.

$^{31}P\{^1H\}$-NMR (162.1 MHz, C_6D_6): δ = 33.44 (d, $^2J_{PP}$ = 14.4 Hz; *P*Ph_3), 53.68 (d, $^2J_{PP}$ = 13.8 Hz; S*P*Ph_2) ppm.

$^{13}C\{^1H\}$-NMR (100.7 MHz, C_6D_6): δ = –6.46 (dd, $^1J_{CP}$ = 114.5 + 100.3 Hz; P*C*P), 26.63 (s, PCy_3*C*$H_{Cy,para}$), 27.06 (s; $SPCy_2$*C*$H_{Cy,para}$), 27.46 (d, $^2J_{CP}$ = 11.5 Hz; PCy_3*C*$H_{Cy,ortho}$), 27.55 - 27.85 (m; $SPCy_2$*C*$H_{Cy,ortho,meta}$, PCy_3*C*$H_{Cy,meta}$), 34.09 (d, $^1J_{CP}$ = 51.0; PCy_3*C*$H_{Cy,ipso}$), 43.66 (dd, $^{1,3}J_{CP}$ = 56.1 + 2.2; $SPCy_2$*C*$H_{Cy,ipso}$) ppm.

HRMS-ESI (m/z): $[M-H]^+$ calcd for M = $C_{31}H_{56}P_2S$, 523.3651; found, 523.3652.

Melting point. 127.3 °C.

1.4 Synthesis of compounds 4

Synthesis of compound 4b

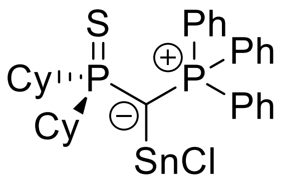

20 mg (0.04 mmol) of compound **3b** and 5.4 mg (0.04 mmol) benzyl potassium were dissolved in 1 ml benzene and stirred for 5 min. Tin dichloride (8.0 mg, 0.041 mmol) was added. THF was added dropwise under stirring until a color change began, then the solution was stirred for 1 h. After filtration, the mixture was left for slow evaporation of the solvent overnight and brown crystals of the product could be recovered. Washing with pentane and subsequent drying yielded deep brown crystals of the product (23.3 mg, 0.035 mmol, 88%).

^1H-NMR (400 MHz, THF-d_8): δ = 0.75 - 2.12 (m, 22H, PCy2S*H*$_{Cy}$), 7.51 - 7.65 (m, 9H,PPh_3*H*$_{Ph,ortho,para}$), 7.76 - 7.87 (m, 6H, PPh_3*H*$_{Ph,meta}$) ppm.

$^{31}P\{^1H\}$-NMR (162 MHz, THF-d_8): δ = 7.48 (d, $^2J_{PP}$ = 5.8 Hz), 66.70 (d, $^2J_{PP}$ = 5.0 Hz) ppm.

$^{13}C\{^1H\}$-NMR (101 MHz, THF-d_8): δ = 15.02 (dd, $^{1,1}J_{CP}$ = 88.6 + 55.6 Hz, P*C*P), 26.76 (d, $^3J_{CP}$ = 39.6 Hz, PCy_2S*C*$_{Cy,meta}$), 27.00 (d, $^4J_{CP}$ = 2.0 Hz, PCy_2S*C*$_{Cy,para}$), 27.53 (dd, $^{2,4}J_{CP}$ = 18.5 + 13.6 Hz, PCy_2S*C*$_{Cy,ortho}$), 42.76 (br s, PCy_2S*C*$_{Cy,ipso}$), 129.84 (d, $^3J_{CP}$ = 11.7 Hz, PPh_2*C*$_{Ph,para}$), 131.27 (dd, $^{1,3}J_{CP}$ = 89.5 + 4.6 Hz, PPh_3*C*$_{Ph,ipso}$), 132.93 (d, $^4J_{CP}$ = 2.9 Hz, PPh_3*C*$_{Ph,para}$), 134.66 (d, $^2J_{CP}$ = 10.0 Hz, PPh_3*C*$_{Ph,ortho}$) ppm.

$^{119}Sn\{^1H\}$-NMR (149 MHz, THF-d_8): δ = 137.08 (dd, $^{2,2}J_{SnP}$ = 234.8 + 108.6 Hz) ppm.

Synthesis of compound 4c

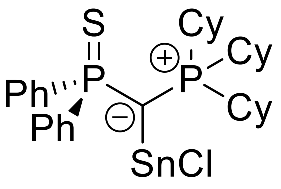

20 mg (0.04 mmol) of compound **3c** and 5.4 mg (0.04 mmol) benzyl potassium were dissolved in 1 ml THF and stirred for 5 min. Tin dichloride (8.0 mg, 0.041 mmol) was added and the solution was stirred for 5 min. After filtration and slow evaporation of the solvent overnight, brown crystals of the product could be recovered. Washing with pentane and subsequent drying yielded deep brown crystals of the product (22.0 mg, 0.033 mmol, 83%).

^{1}H-NMR (400 MHz, THF-d_8): δ = 7.99 - 8.09 (m, 4H, $PPh_2SH_{Ph,meta}$), 7.05 - 7.15 (m, 6H, $PPh_2SH_{Ph,ortho,para}$), 1.02 - 1.93 (m, 33H, PCy_3H_{Cy}) ppm.

^{31}P{^{1}H}-NMR (162 MHz, THF-d_8): δ = 20.54 (d, $^2J_{PP}$ = 6.9 Hz, PCy_3), 37.75 (d, $^2J_{PP}$ = 6.8 Hz, PPh_2S) ppm.

^{13}C{^{1}H}-NMR (101 MHz, THF-d_8): δ = 17.23 (dd, $^{1,1}J_{CP}$ = 71.7 + 66.0 Hz, PCP), 26.91 (s, $PCy_3C_{Cy,para}$), 28.05 (d, $^2J_{CP}$ = 11.8 Hz, $PCy_3C_{Cy,ortho}$), 28.46 (s, $PCy_3C_{Cy,meta}$), 36.80 (dd, $^{1,3}J_{CP}$ = 50.9 + 5.4 Hz, $PCy_3C_{Cy,ipso}$), 128.73 - 129.37 (m, $PPh_2SC_{Ph,ortho,para}$), 132.51 (d, $^3J_{CP}$ = 11.6 Hz, $PPh_2SC_{Ph,meta}$), 141.77 (d, $^1J_{CP}$ = 74.4 Hz, $PPh_2SC_{Ph,ipso}$) ppm.

^{119}Sn{^{1}H}-NMR (149 MHz, THF-d_8): δ = 189.97 (d, $^2J_{SnP}$ = 299.4 Hz) ppm.

Synthesis of compound 4d

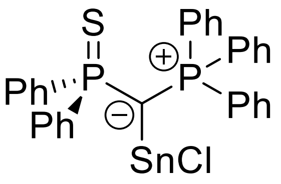

20 mg (0.04 mmol) of compound **3d** and 5.4 mg (0.04 mmol) benzyl potassium were dissolved in 1 ml THF and stirred for 5 min. Tin dichloride (8.0 mg, 0.041 mmol) was added and the solution was stirred for 5 min. After filtration and slow evaporation of the solvent overnight, brown crystals of the product could be recovered. Washing with pentane and subsequent drying yielded deep brown crystals of the product (23.8 mg, 0.037 mmol, 92%).

^{1}H-NMR (400 MHz, CD_2Cl_2): δ = 7.58 - 7.68 (m, 4H, $PPh_2SH_{Ph,meta}$), 7.27 - 7.57 (m, 18H), 7.20 - 7.26 (m, 3H, $PPh_3H_{Ph,para}$) ppm.

^{31}P{^{1}H}-NMR (162 MHz, CD_2Cl_2): δ = 10.98 (s, PPh_3), 40.21 (s, PPh_2S) ppm.

^{13}C{^{1}H}-NMR (101 MHz, CD_2Cl_2): δ = 38.22 (d, J = 77.2 Hz), 128.18 (d, J = 5.8 Hz), 128.41 (d, J = 12.5 Hz), 128.93 (d, J = 12.5 Hz), 129.15 (d, J = 12.0 Hz), 131.24, 131.38 (d, J = 12.0 Hz), 131.91 - 132.67 (m), 133.54 (d, J = 10.3 Hz), 133.74 ppm.

^{119}Sn{^{1}H}-NMR (149 MHz, CD_2Cl_2): δ = 122.86 (dd, $^2J_{SnP}$ = 251.9 + 102.0 Hz) ppm.

1.5 Synthesis of compound 5

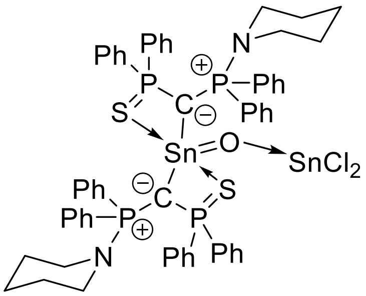

40 mg (0.080 mmol) **3a** and 12 mg benzyl potassium (0.092 mmol) were dissolved in 0.7 mL toluene in a J-Young NMR tube and were stirred for 5 min. After filtration, 15.5 mg (0.080 mmol) $SnCl_2$ and 1.3 mL THF were added, and the solution was stirred for 2 min. The resulting solution was split into two J. Young NMR tubes, and the atmosphere was changed to N_2O. After three days of shaking, **5** crystallized on the walls of the NMR tube as a colorless solid. The crystals were suitable for X-ray diffraction analysis and were obtained in a yield of 49.2 mg (48.9 mg, 0.037 mmol, 93 %).

^{1}H-NMR (400.3 MHz, $CDCl_3$): δ = 1.38 - 1.52 (m, 4H; C$H_{2,\text{Pip},3}$), 1.52 - 1.71 (m, 8H; C$H_{2,\text{Pip},2,4}$), 2.79 - 2.99 (m, 4H; C$H_{2,\text{Pip},1,5}$), 3.46 - 3.64 (m, 4H; C$H_{2,\text{Pip},1,5}$), 6.83 - 7.02 (m, 8H; NPC$H_{\text{Ph},ortho}$), 7.03 - 7.16 (m, 8H; NPC$H_{\text{Ph},meta,\text{para}}$), 7.20 - 7.30 (m, 4H; NPC$H_{\text{Ph},meta}$), 7.40 - 7.51 (m, 4H; SPC$H_{\text{Ph},para}$), 7.51 - 7.74 (m, 10H; SPC$H_{\text{Ph,ortho},meta}$), 7.75 - 7.96 (m, 6H; SPC$H_{\text{Ph,ortho},meta}$) ppm.

^{31}P{^{1}H}-NMR (162.1 MHz, $CDCl_3$): δ = 29.8 (d, $^2J_{PP}$ = 3.7 Hz; S*P*Ph_2), 41.3 (d, $^2J_{PP}$ = 3.3 Hz; N*P*Ph_2) ppm.

^{13}C{^{1}H}-NMR (100.7 MHz, $CDCl_3$): δ = 24.4 (s; N*C*$H_{\text{Pip},3}$), 26.3 (d, $^3J_{CP}$ = 5.6 Hz; N*C*$H_{\text{Pip},2,4}$), 47.7 (s; N*C*$H_{\text{Pip},1,5}$), 128.4 (d, $^2J_{CP}$ = 13.0 Hz; NP*C*$H_{\text{Ph},ortho}$), 128.8 (d, $^2J_{CP}$ = 13.2 Hz; NP*C*$H_{\text{Ph},ortho}$), 129.1 (d, $^3J_{CP}$ = 12.9 Hz; NP*C*$H_{\text{Ph},meta}$), 129.3 (d, $^3J_{CP}$ = 12.9 Hz; NP*C*$H_{\text{Ph},meta}$), 130.9 (s; NP*C*$H_{\text{Ph},para}$), 131.0 (d, $^2J_{CP}$ = 11.5 Hz; SP*C*$H_{\text{Ph},ortho}$), 132.0 (d, $^2J_{CP}$ = 11.8 Hz; SP*C*$H_{\text{Ph},ortho}$), 132.4 (d, $^3J_{CP}$ = 10.7 Hz; SP*C*$H_{\text{Ph},meta}$), 132.8 (s; SP*C*$H_{\text{Ph},para}$), 133.2 (s; SP*C*$H_{\text{Ph},para}$), 133.6 (d, $^3J_{CP}$ = 10.4 Hz; SP*C*$H_{\text{Ph},meta}$) ppm.

Due to the very poor solubility of **5**, even after 16,000 scans, the ^{13}C{^{1}H}-NMR signals of the ipso carbons of the phenyl rings, as well as that of the bridging carbons in the ylides, could not be detected. Attempts to record a ^{119}Sn NMR spectrum were unsuccessful due to the low solubility.

Single crystals were used for elemental analysis. Since the crystals contained one molecule of THF, reference values for **5** also include one equivalent of THF.

Anal. Calcd for $C_{64}H_{68}Cl_2Sn_2N_2P_4S_2O_2$: C, 55.16; H, 4.92; S, 4.60; N, 2.01. Found: C, 54.96; H, 4.68; S, 4.34; N, 2.21.

Melting point. 215.8 °C (decomposition).

It is crucial to note that while the reaction is reproducible, it proved impossible to scale up. When performed in a Schlenk tube instead of a J-Young NMR tube, the reaction consistently resulted in significant decomposition.

1.6 Synthesis of compounds 6

Synthesis of compound 6-BF_4

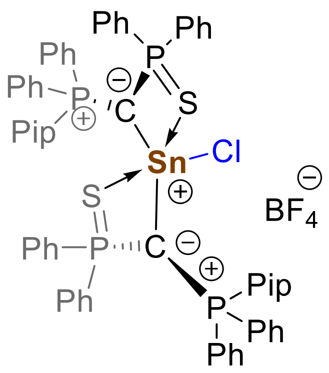

26 mg (0.02 mmol) of **5** were dissolved in 0.7 ml CD_2Cl_2 and the resulting solution was cooled to –30 °C. 2 eq. of $AgBF_4$ were added and the solution was stirred for 15 min. The product was confirmed by ^{1}H-NMR and ^{31}P{^{1}H}-NMR spectroscopy. Single crystals suitable for X-ray diffraction analyses were grown by slow vapor diffusion of pentane into a saturated solution of compound **6-BF4** in CD_2Cl_2. Attempts to purify the compound failed due to repeated ylide formation.

^{1}H-NMR (400.3 MHz, CD_2Cl_2): δ = 1.40 - 1.67 (m, 8H; C$H_{2,\text{Pip}}$), 2.03 - 2.24 (br, 4H; C$H_{2,\text{Pip}}$), 2.87 - 3.02 (br, 2H; C$H_{2,\text{Pip}}$), 3.40 - 3.53 (br, 2H; C$H_{2,\text{Pip}}$), 4.10 - 4.48 (br, 4H; C$H_{2,\text{Pip}}$), 6.74 - 8.02 (m, 40H; PCH_{Ph}) ppm.

^{31}P{^{1}H}-NMR (162.1 MHz, CD_2Cl_2): δ = 30.4 (br, S*P*Ph_2), 41.9 (br, N*P*Ph_2) ppm.

Synthesis of compound 6-$SnCl_6$

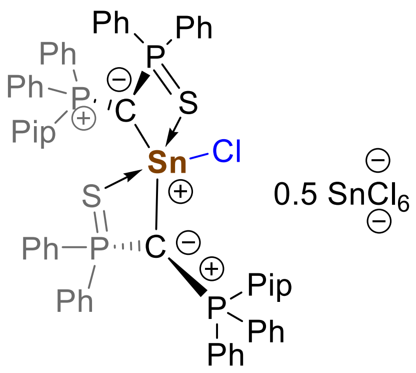

26 mg (0.02 mmol) of **5** were dissolved in 0.7 ml $CDCl_3$. 2 drops of benzaldehyde (<u>not dried)</u> were added to the reaction mixture and the solution was stirred for 15 min. The formation of cation **6** was confirmed by ^{1}H-NMR and ^{31}P{^{1}H}-NMR spectroscopy. Single crystals suitable for X-ray diffraction analyses were grown by slow vapor diffusion of pentane into a saturated solution of compound **6-$SnCl_6$** in $CDCl_3$. Attempts to purify the compound failed due to repeated ylide formation.

^{1}H-NMR (400.3 MHz, $CDCl_3$): δ = 1.48 - 1.68 (m, 12H; C$H_{2,\text{Pip}}$), 3.02 - 3.14 (br, 8H; C$H_{2,\text{Pip}}$), 7.32 - 7.47 (m, 26H; PCH_{Ph}), 8.01 - 8.12 (m, 6H; PCH_{Ph}), 8.22 - 8.30 (m, 8H; PCH_{Ph}) ppm.

^{31}P{^{1}H}-NMR (162.1 MHz, $CDCl_3$): δ = 32.6 (d, $^{2}J_{PP}$ = 6.5 Hz, S*P*Ph_2), 44.5 (d, $^{2}J_{PP}$ = 6.7 Hz, N*P*Ph_2) ppm.

1.7 List of substrates tested in reactivity studies with stannanone 5

Below is a list of additional reactants used in reactivity studies with stannanone **5**. These substrates either showed no reactivity or reacted in a highly unselective manner. The CAS numbers of the respective compounds are provided together with their names or formula.

Table S1: List of additional reactants used in reactivity studies with stannanone **5**.

N2	7727-37-9
H2	1333-74-0
Potassium tert-butylate	865-47-4
CO	630-08-0
CO2	124-38-9
NH3	7664-41-7
CS2	75-15-0
AgSbF6	26042-64-8
p-Dimethylaminopyridine	1122-58-3
ClMgPh	100-59-4
TMSCl	75-77-4
TMSN3	4648-54-8
HBpin	25015-63-8
ZnF2	7783-49-5
Phenylsilane	694-53-1
Diphenylsilane	775-12-2
Phenylacetylene	536-74-3
Phenyl disulfide	882-33-7
1-Ethynyl-4-dimethylaniline	17573-94-3
Bis(trimethylsilyl)acetylene	14630-40-1
4-Ethynylanisole	768-60-5
Trimethylsilylacetylene	1066-54-2
SO2	7446-09-5
3,5-Di-tert-butyl-o-benzochinon	3383-21-9

2. NMR spectra of all compounds

2.1 NMR spectra of compounds 2

NMR spectra of compound 2-O

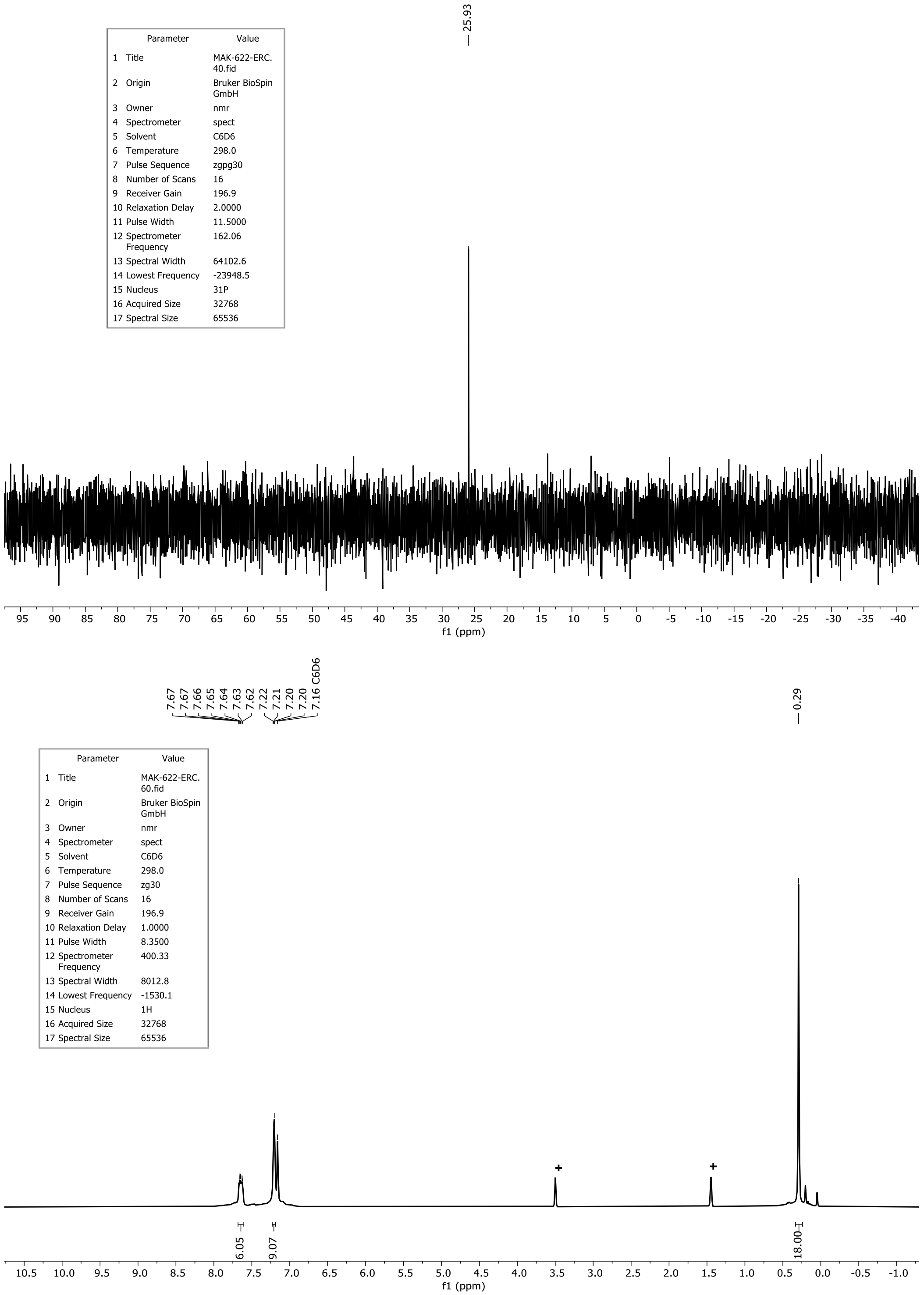

Figure S1: $^{31}P\{^1H\}$ NMR and ^1H NMR spectrum of compound **2-O** in Benzne-d_6 with a residual THF-d_8 (δ = 3.49 and 1.44 ppm) is assigned with **+**.

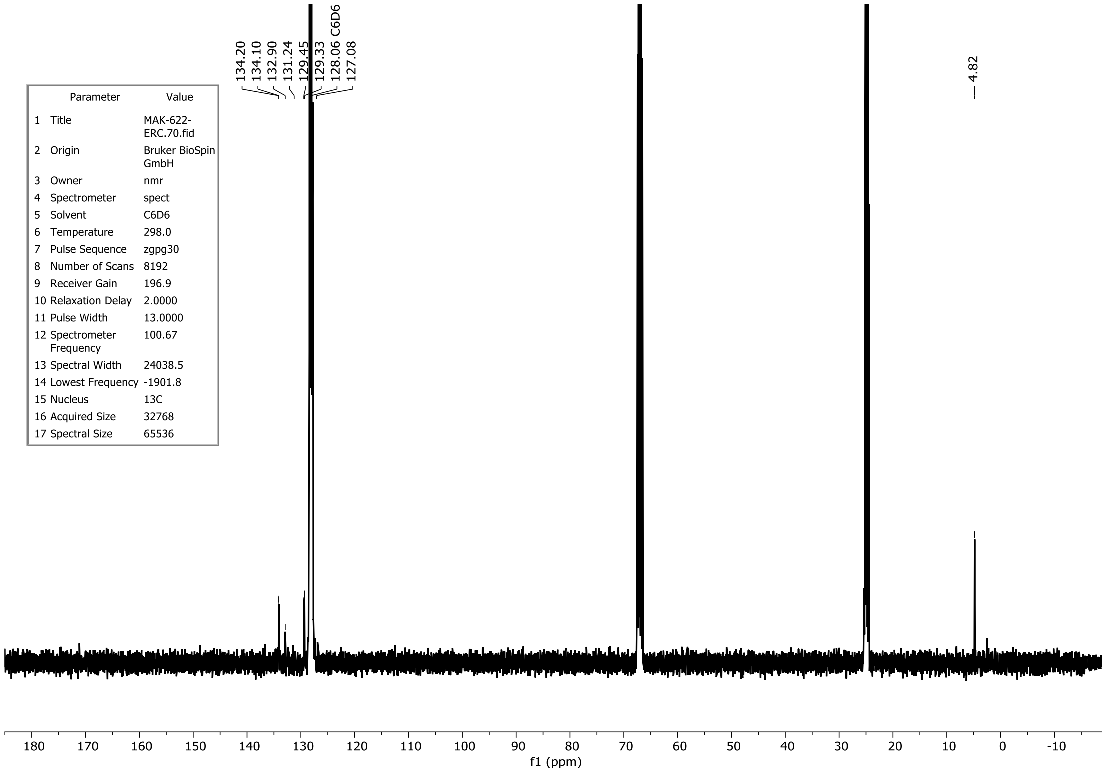

Figure S2: $^{13}C\{^{1}H\}$ NMR of compound **2-O** in Benzne-d_6.

"}

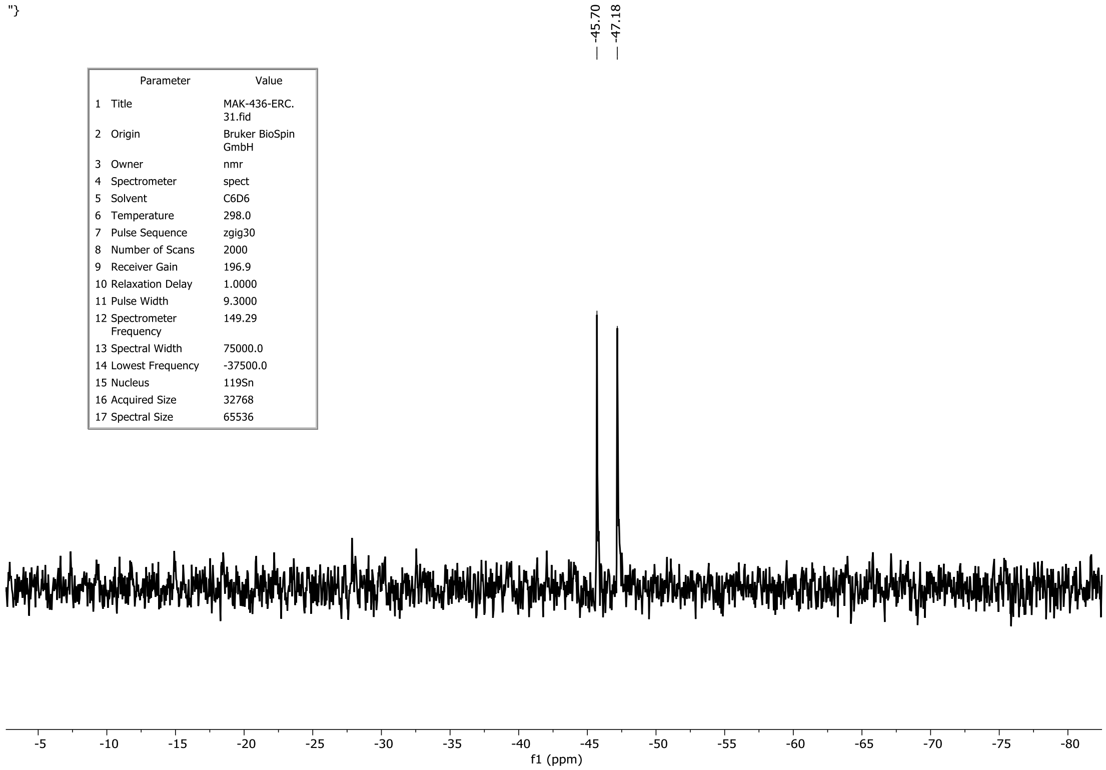

Figure S3: $^{119}Sn\{^{1}H\}$ NMR spectrum of **2-O** in Benzene-d_6.

NMR spectra of compound 2-S

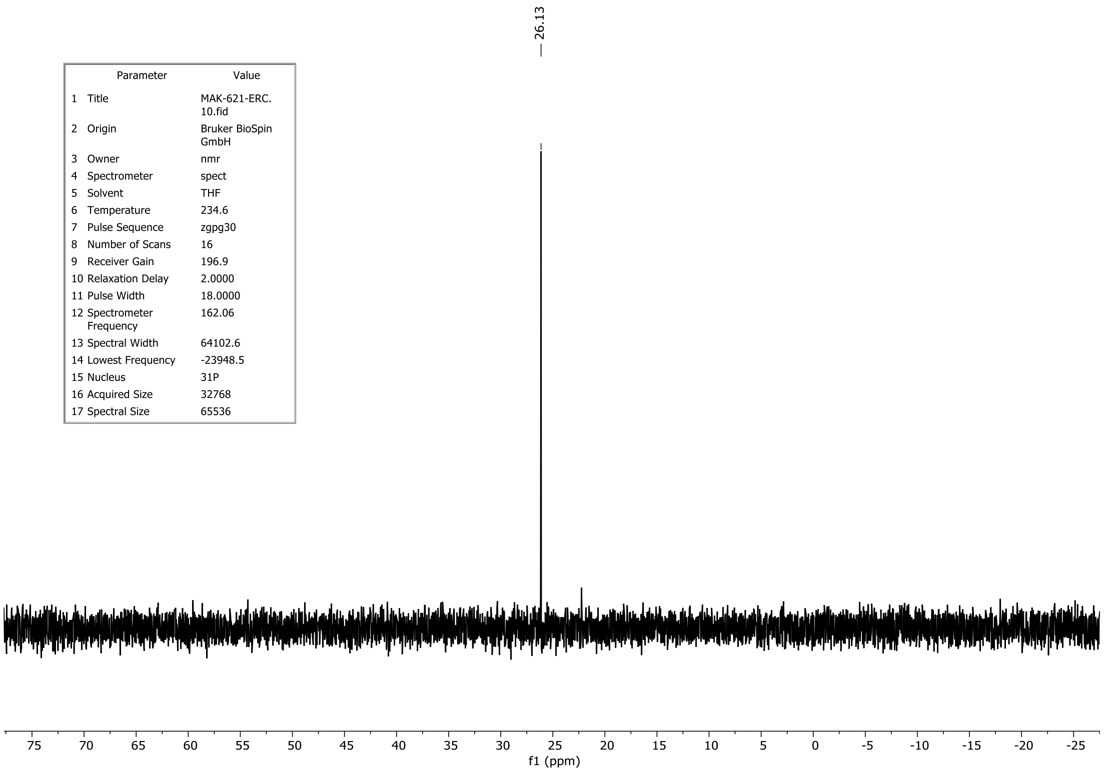

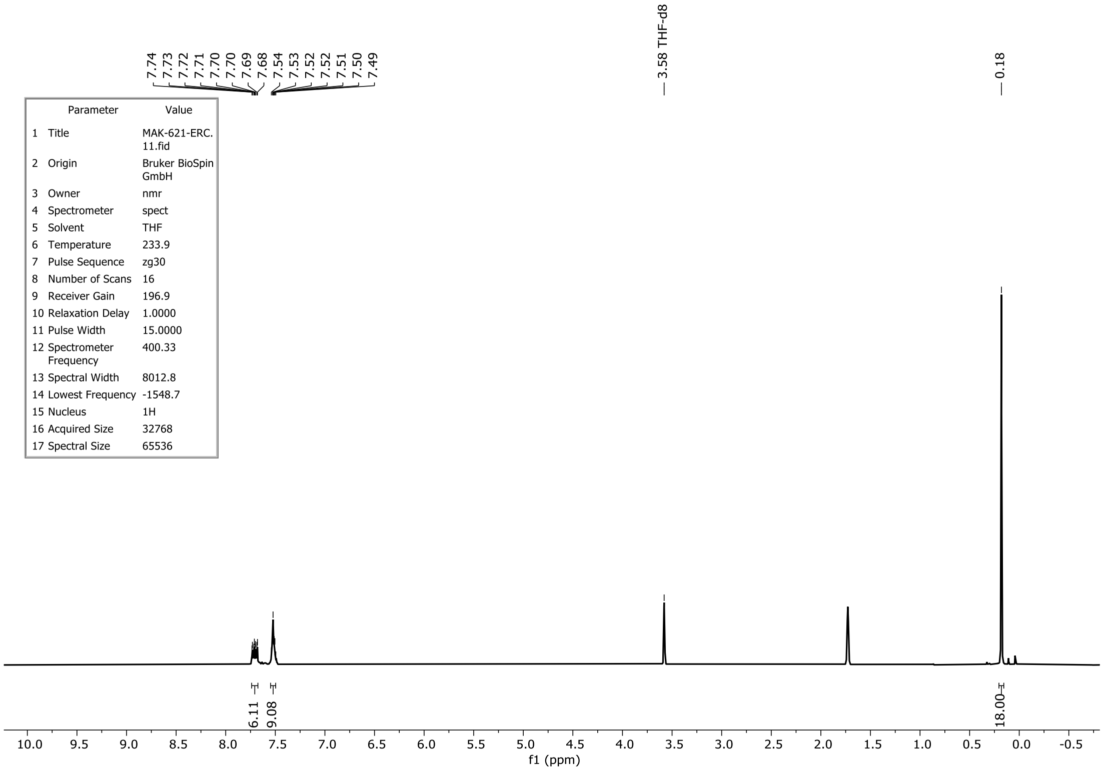

Figure S4: $^{31}P\{^1H\}$ NMR and ^1H NMR spectrum of compound **2-S** in THF-d_8.

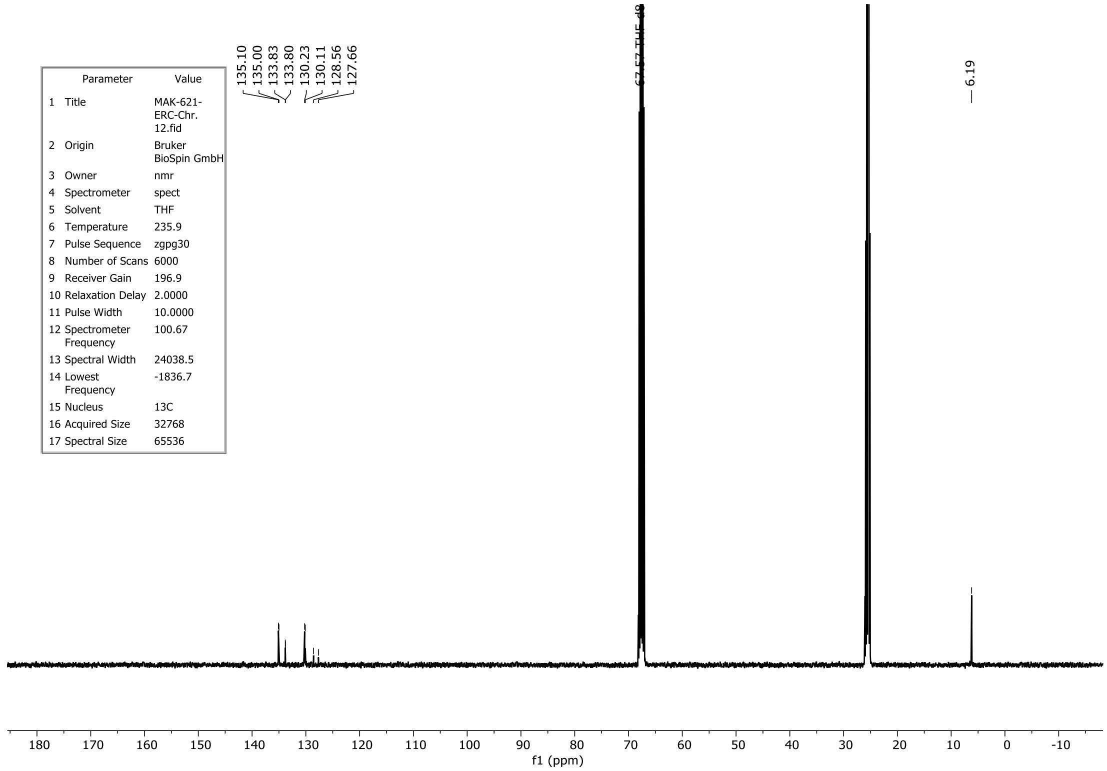

Figure S5: $^{13}C\{^{1}H\}$ NMR of compound **2-S** in THF-d_8.

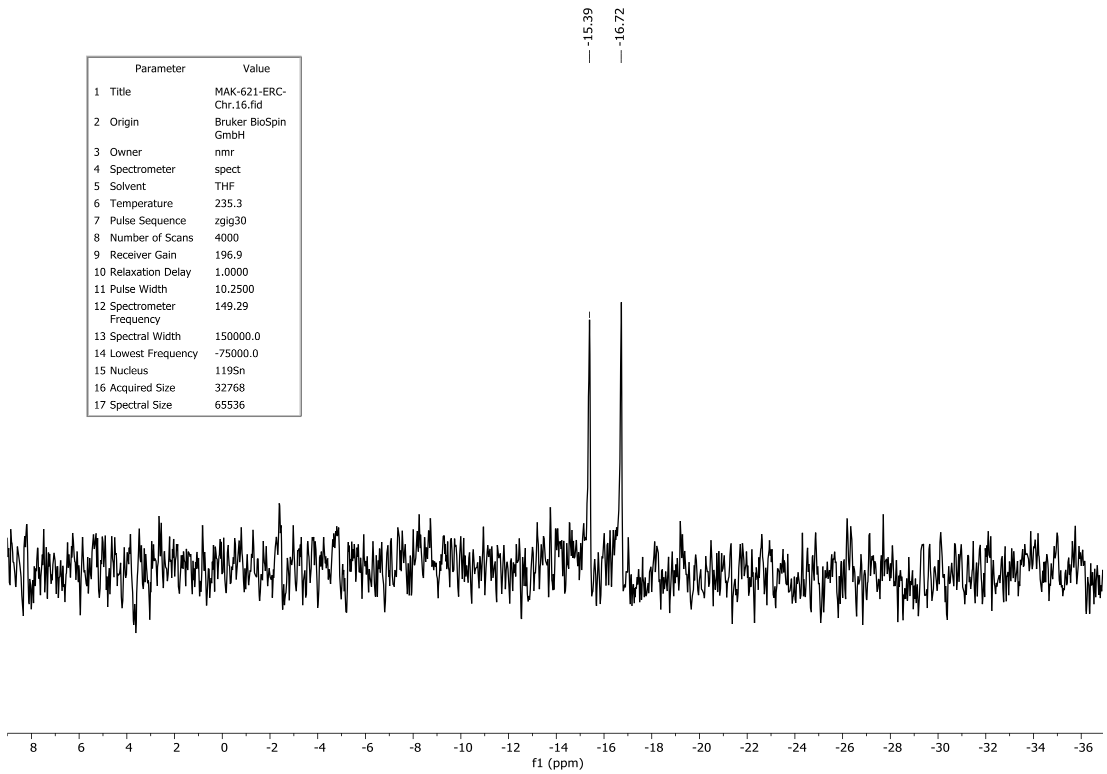

Figure S6: $^{119}Sn\{^{1}H\}$ NMR spectrum of **2-S** in THF-d_8.

2.2 NMR spectra of compounds 3

NMR spectra of compound 3c-Pre

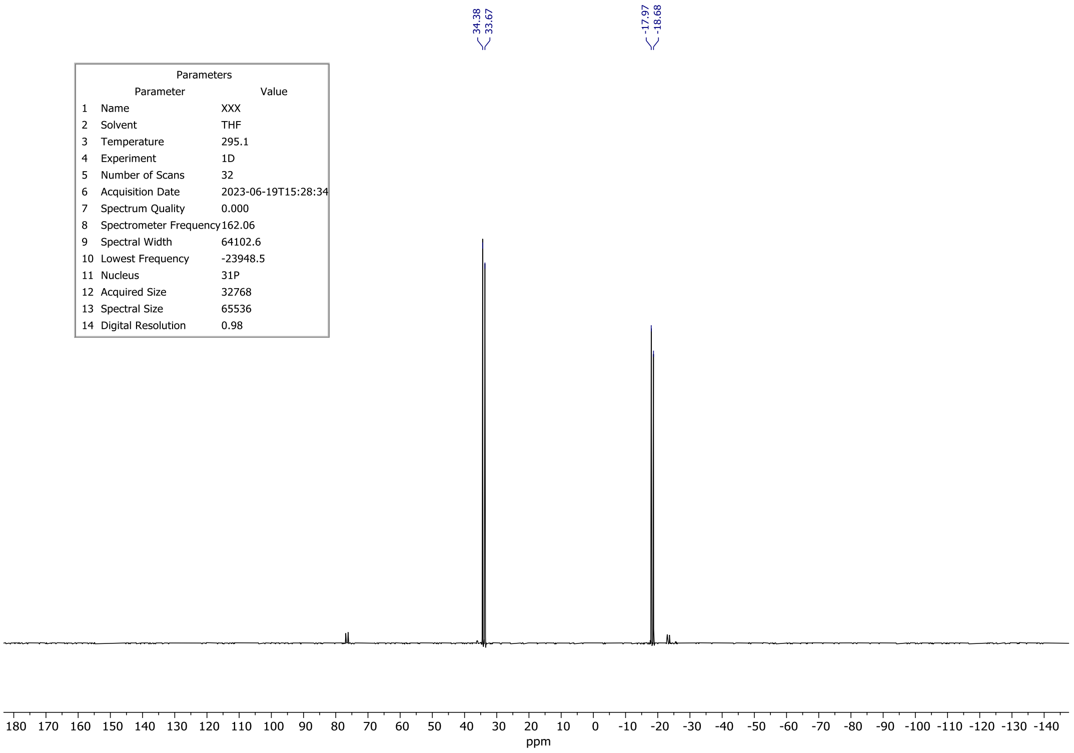

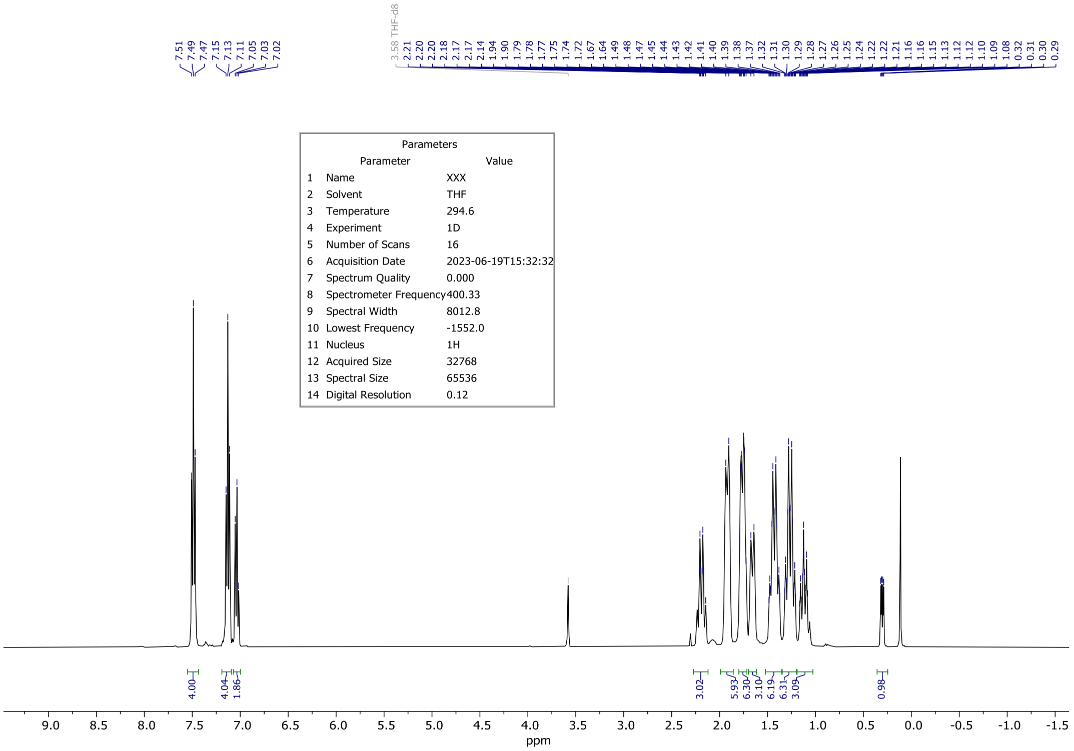

Figure S7: $^{31}P\{^{1}H\}$ NMR and ^{1}H NMR spectrum of compound **3c-Pre** in THF-d_8. ^{1}H NMR spectrum shows small amount of grease.

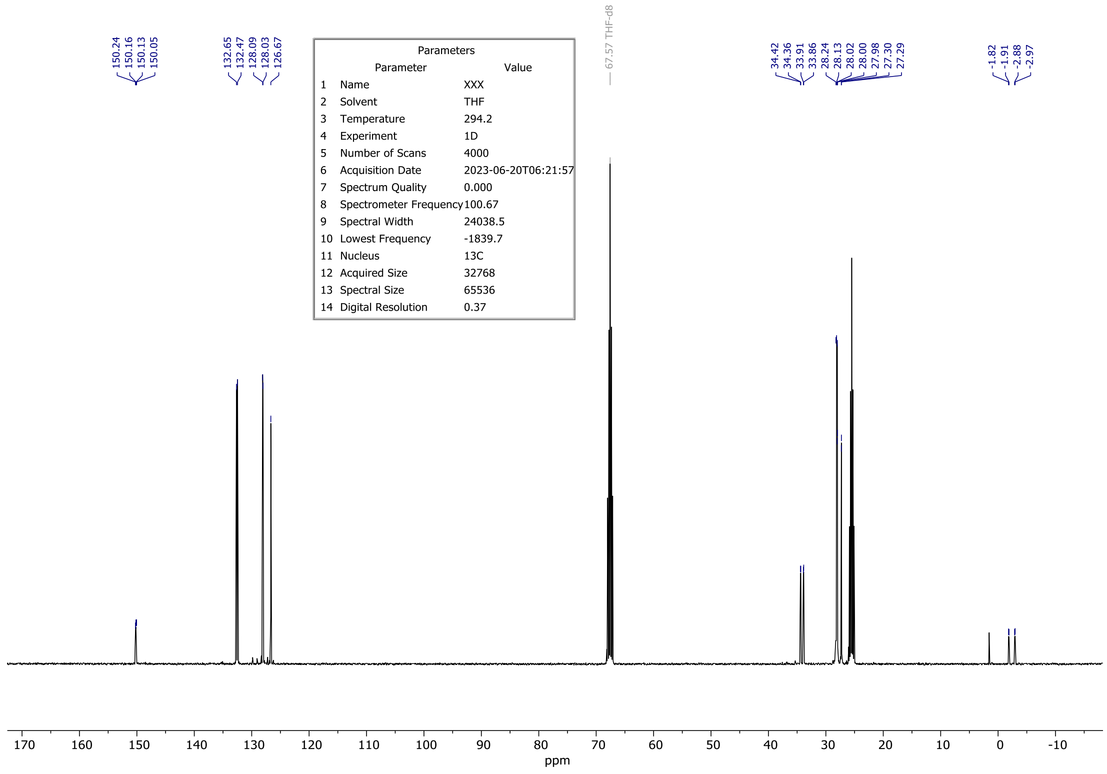

Figure S8: $^{13}C\{^{1}H\}$ NMR spectrum of compound **3c-Pre** in THF-d_8.

NMR spectra of compound 3c

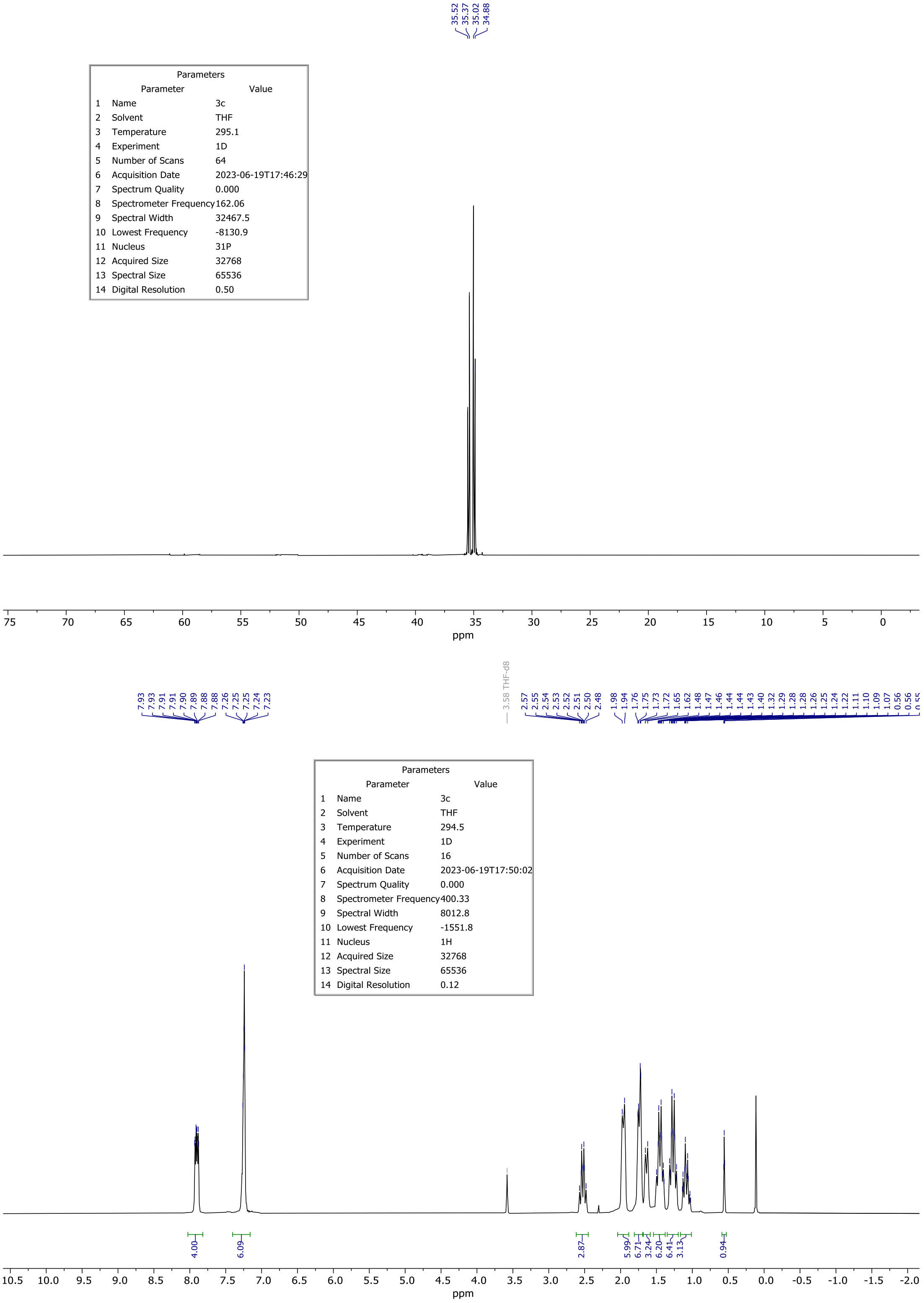

Figure S9: $^{31}P\{^{1}H\}$ NMR and ^{1}H NMR spectrum of compound **3c** in THF-d_8. ^{1}H NMR spectrum shows residual grease (peak at 0.1 ppm).

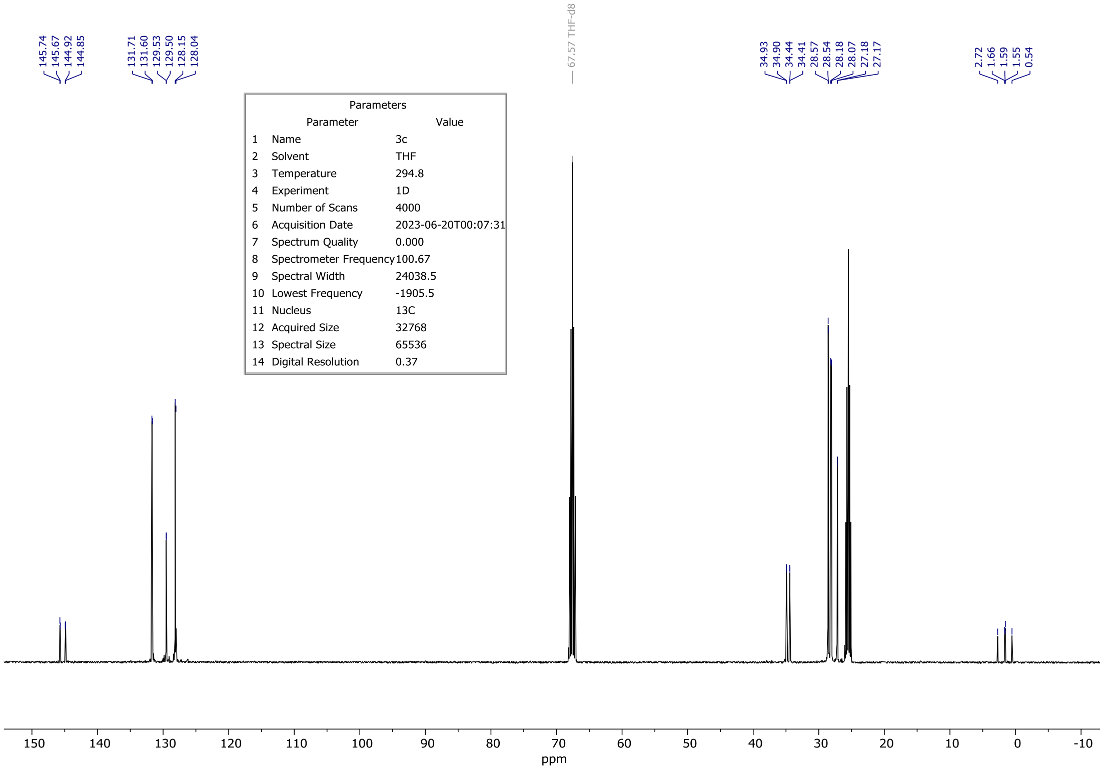

Figure S10: $^{13}C\{^{1}H\}$ NMR spectrum of compound **3c** in THF-d_8.

NMR spectra of compound 3e-S

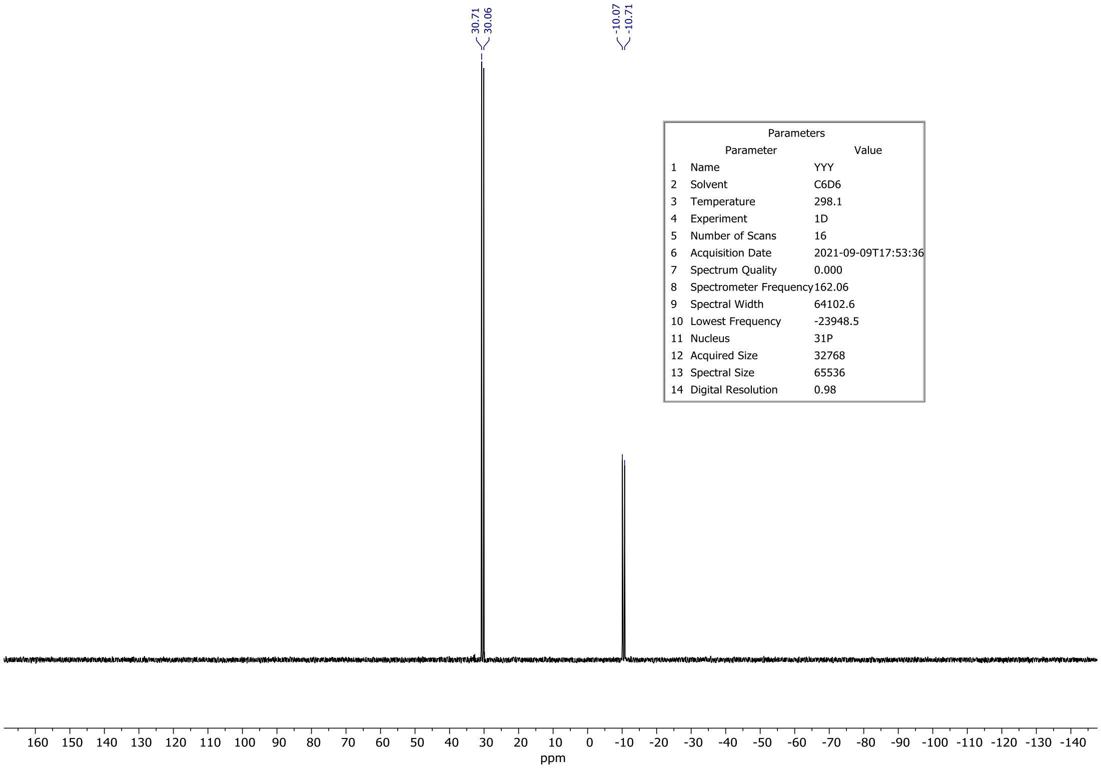

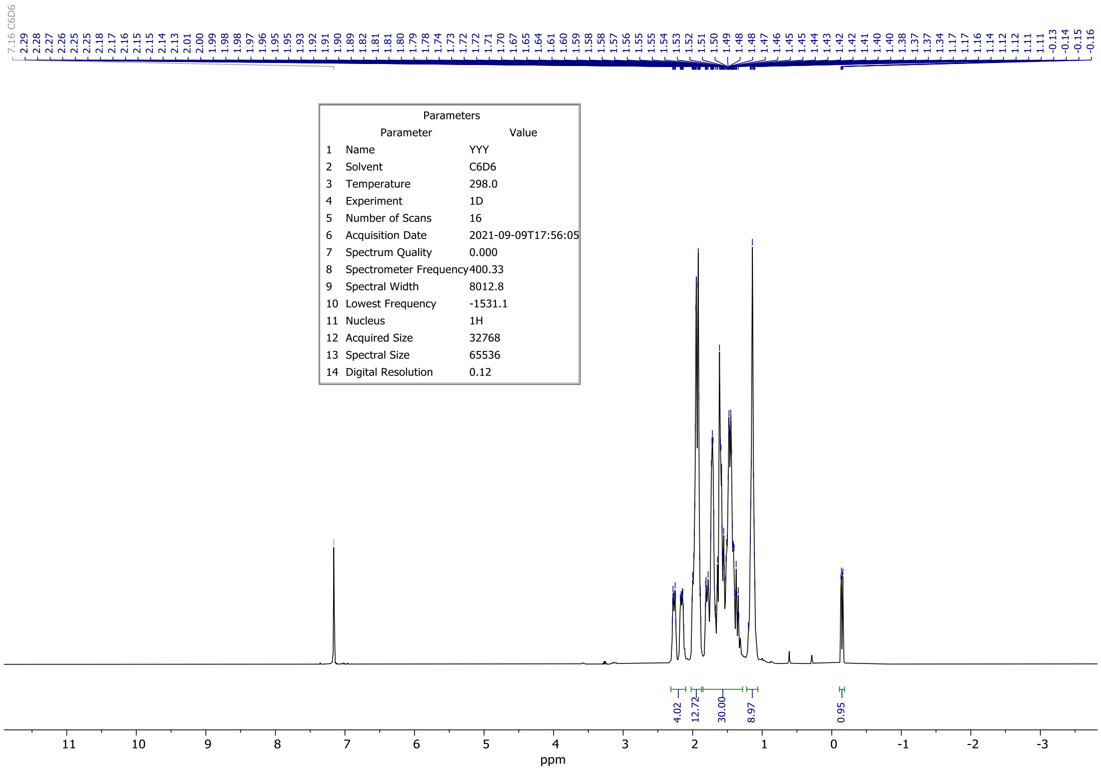

Figure S11: $^{31}P\{^{1}H\}$ NMR and ^{1}H NMR spectrum of compound **3e-Pre** in C_6D_6.

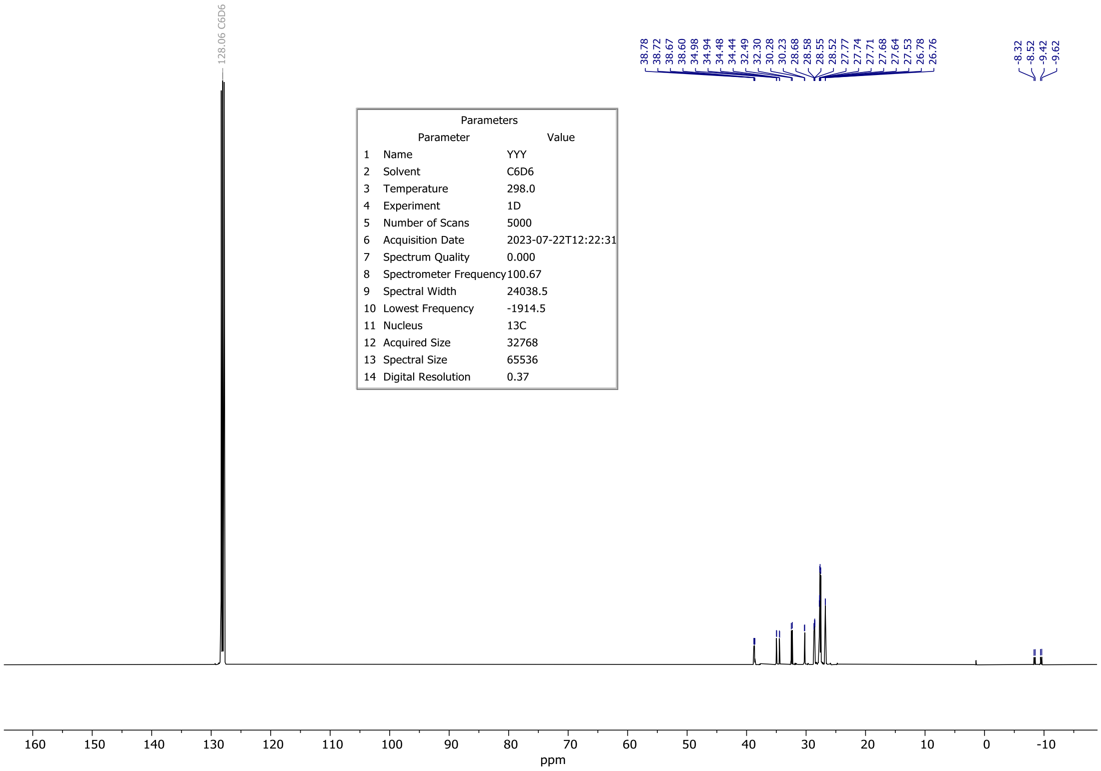

Figure S12: $^{13}C\{^{1}H\}$ NMR spectrum of compound **3e-Pre** in C_6D_6.

NMR spectra of compound 3e

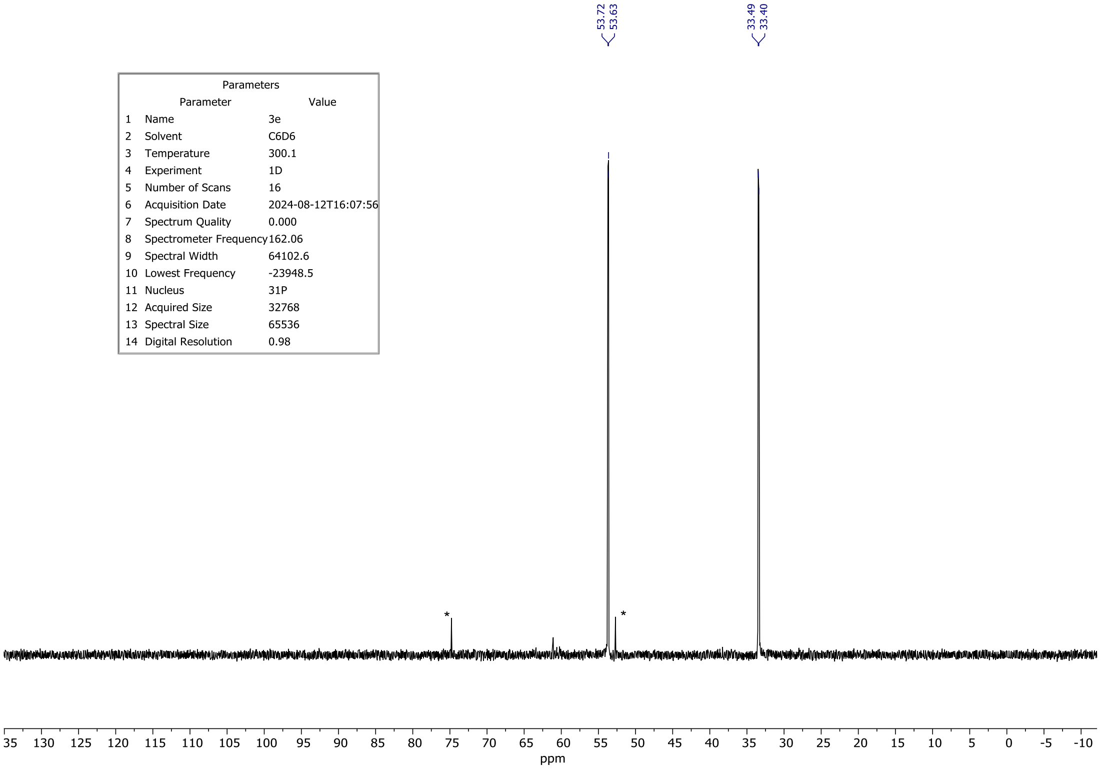

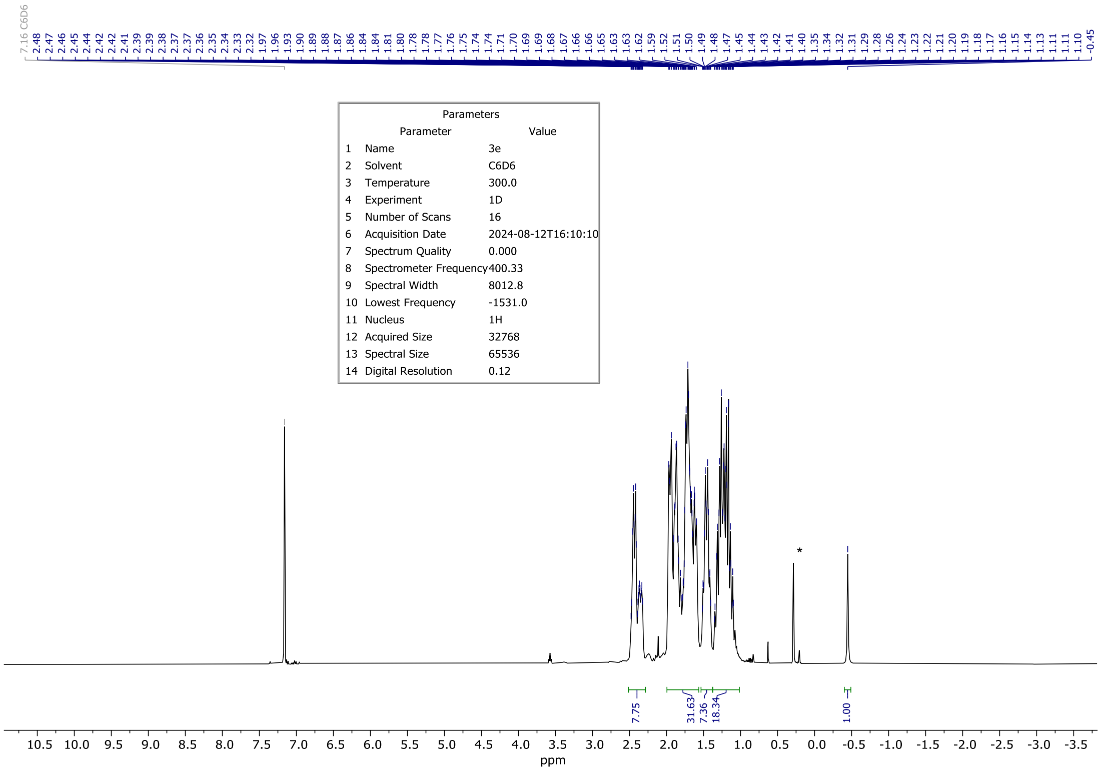

Figure S13: $^{31}P\{^{1}H\}$ NMR and ^{1}H NMR spectrum of compound **3e** in C_6D_6. ^{1}H NMR spectrum shows small amount of residual grease.

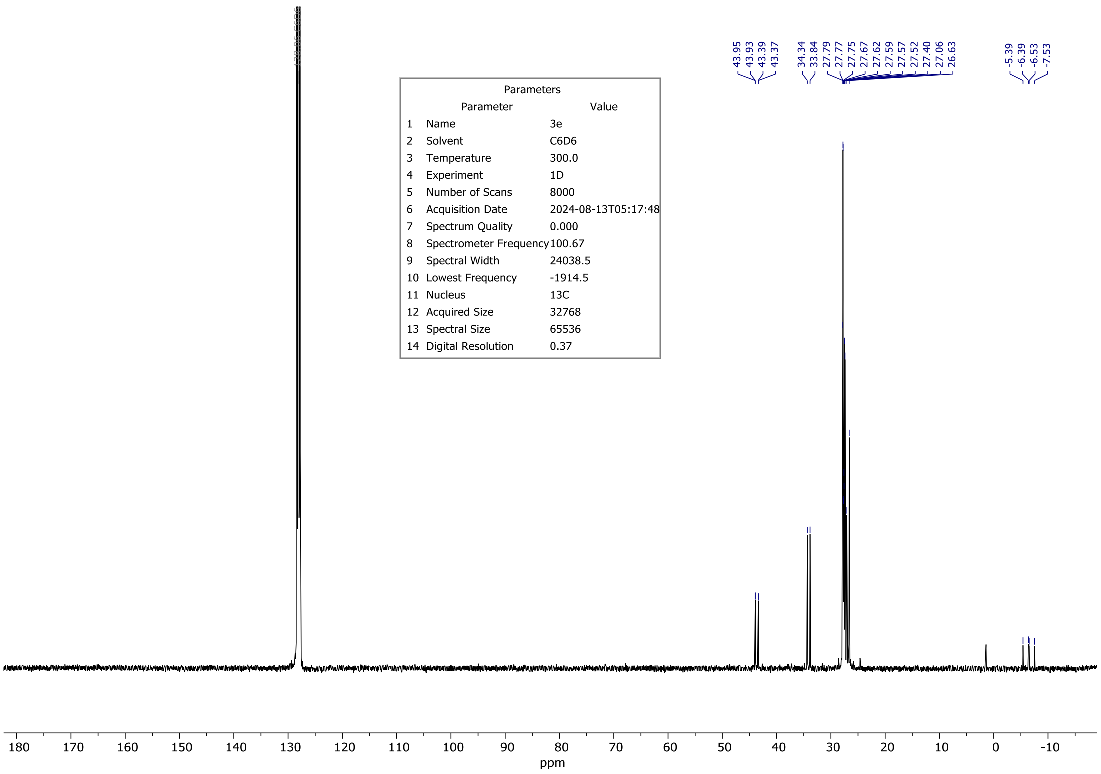

Figure S14: $^{13}C\{^{1}H\}$ NMR spectrum of compound **3e** in C_6D_6.

2.3 NMR spectra of compounds 4

NMR spectra of compound 4b

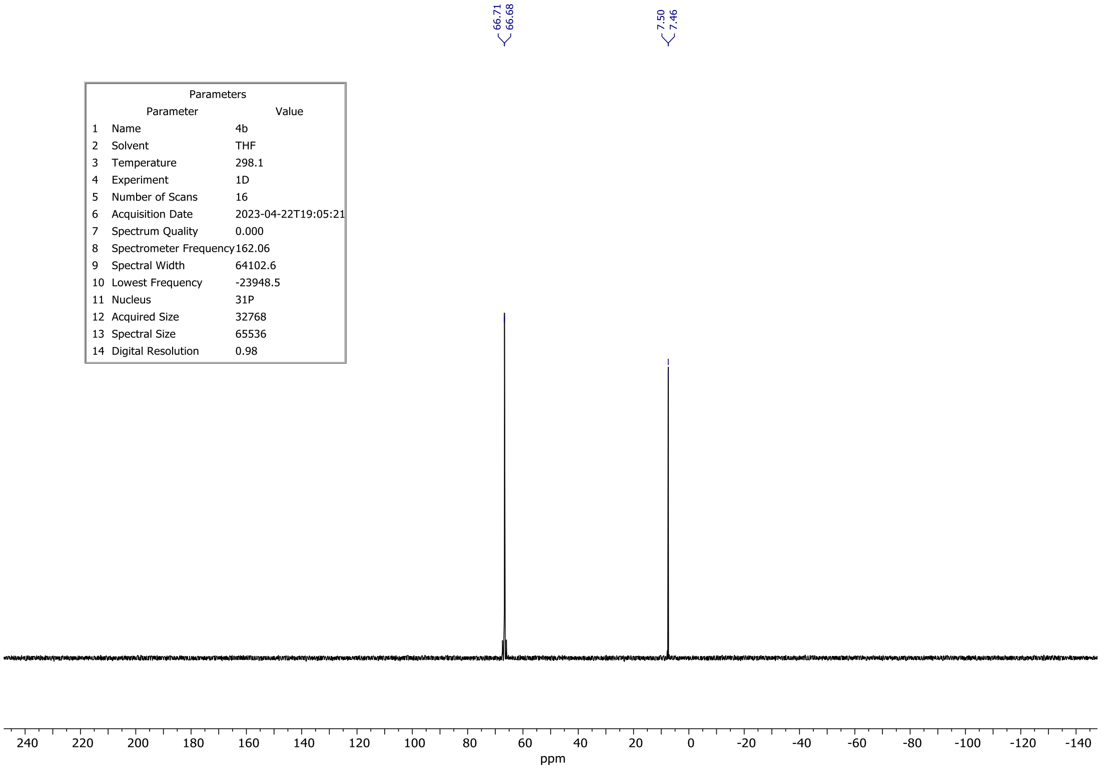

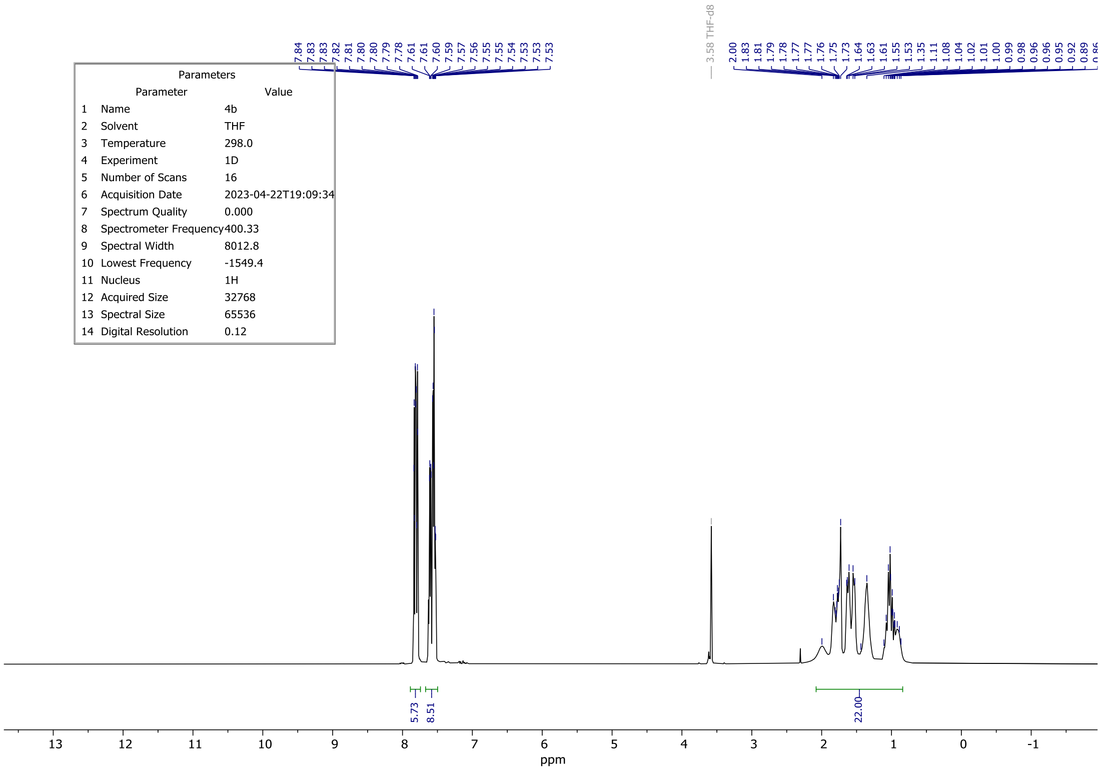

Figure S15: $^{31}P\{^{1}H\}$ NMR and ^{1}H NMR spectrum of compound **4b** in THF-d_8.

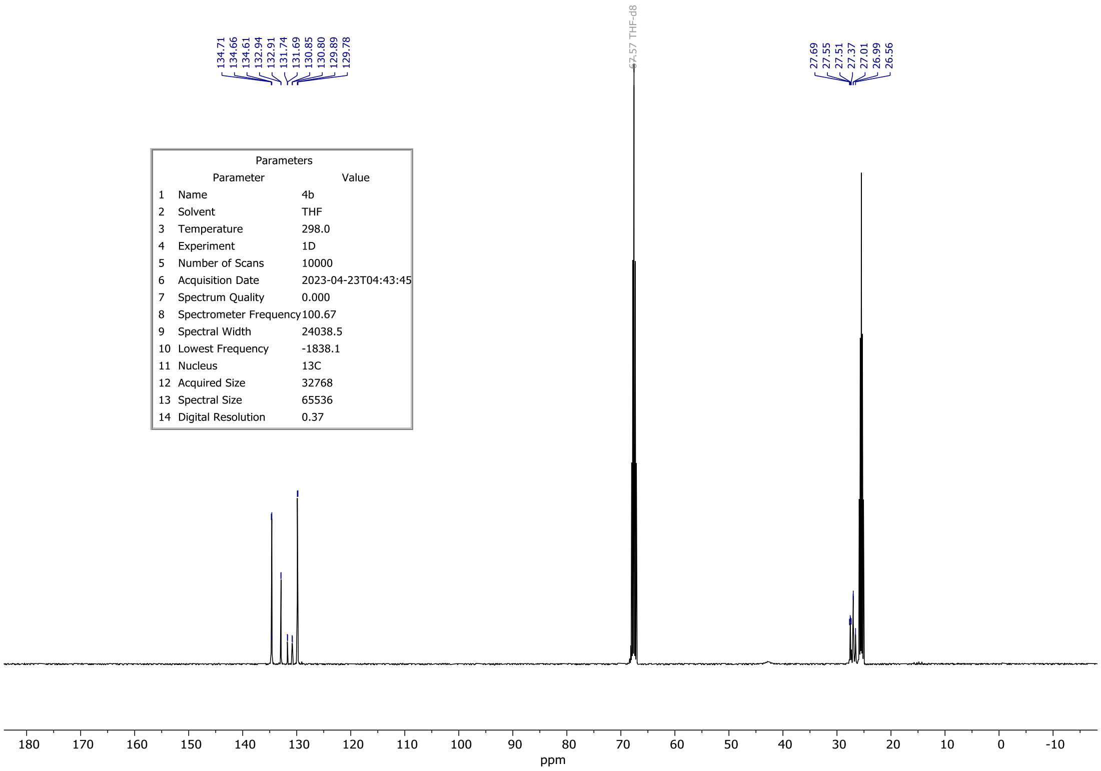

Figure S16: $^{13}C\{^1H\}$ NMR spectrum of compound **4b** in THF-d_8.

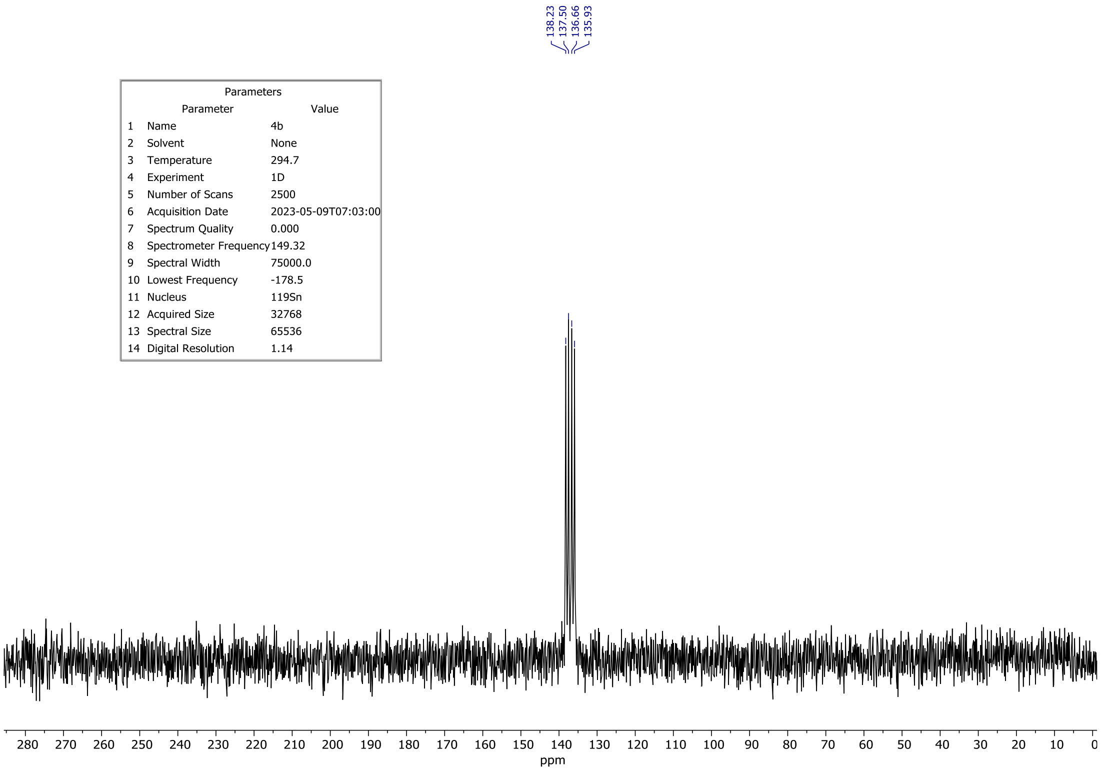

Figure S17: $^{119}Sn\{^1H\}$ NMR spectrum of compound **4b** in THF.

NMR spectra of compound 4c

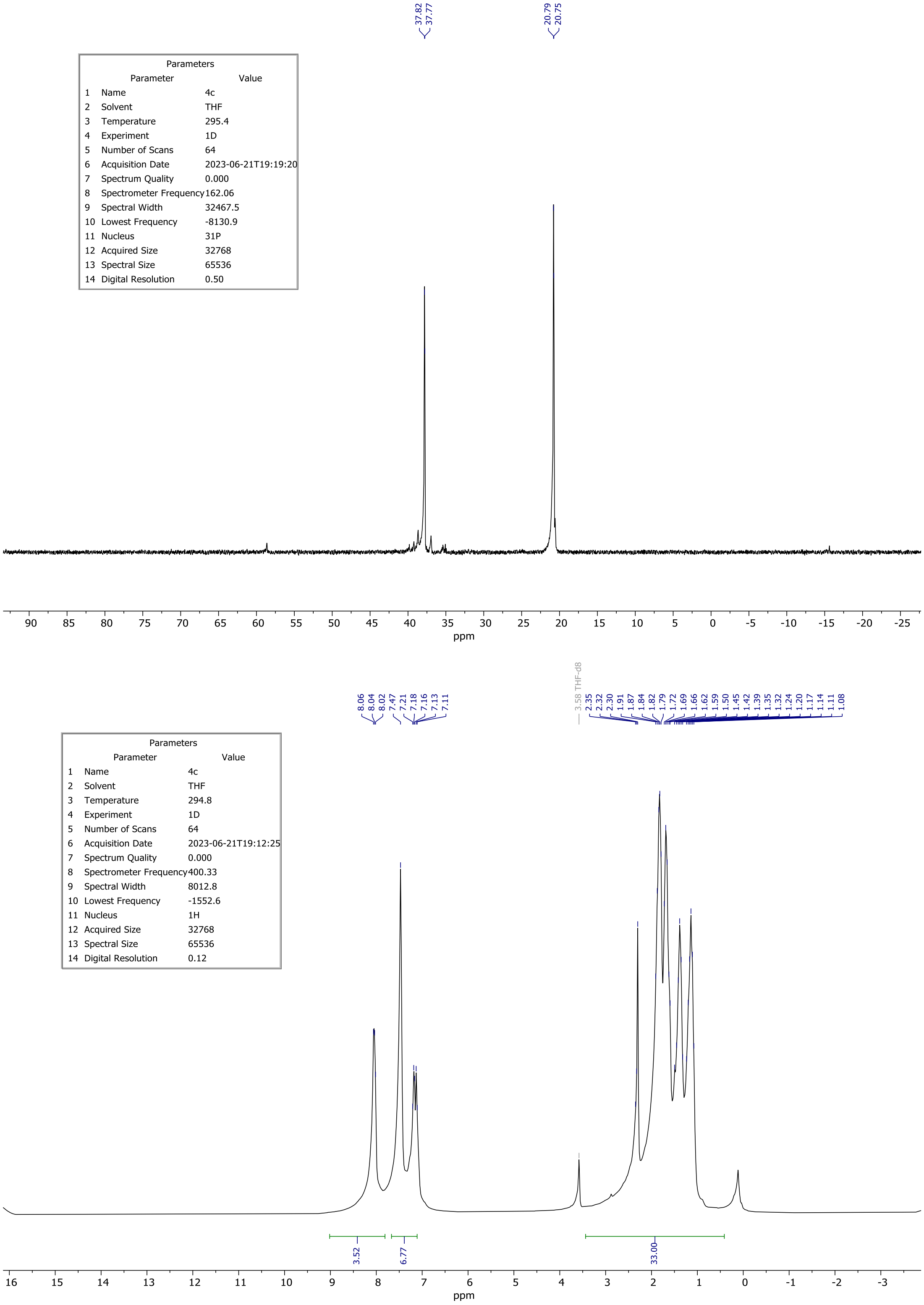

Figure S18: $^{31}P\{^1H\}$ NMR and ^1H NMR spectrum of compound **4c** in THF-d_8. ^1H NMR spectrum shows small amount of grease.

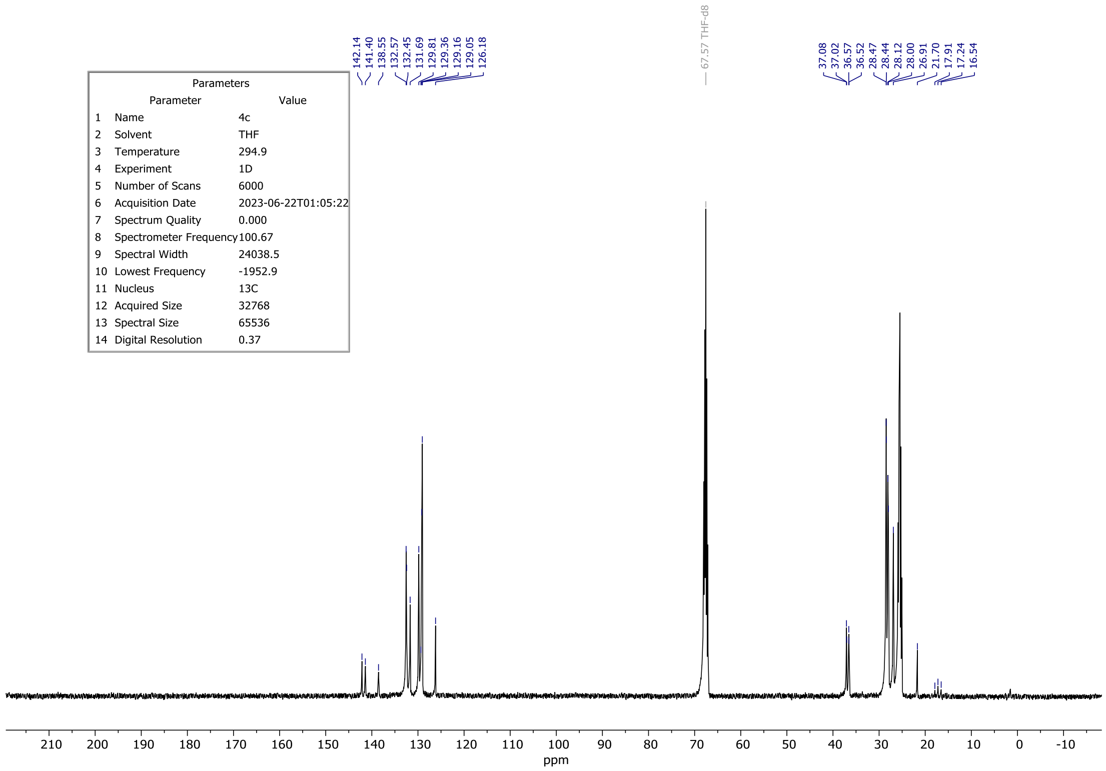

Figure S19: $^{13}C\{^1H\}$ NMR spectrum of compound **4c** in THF-d_8.

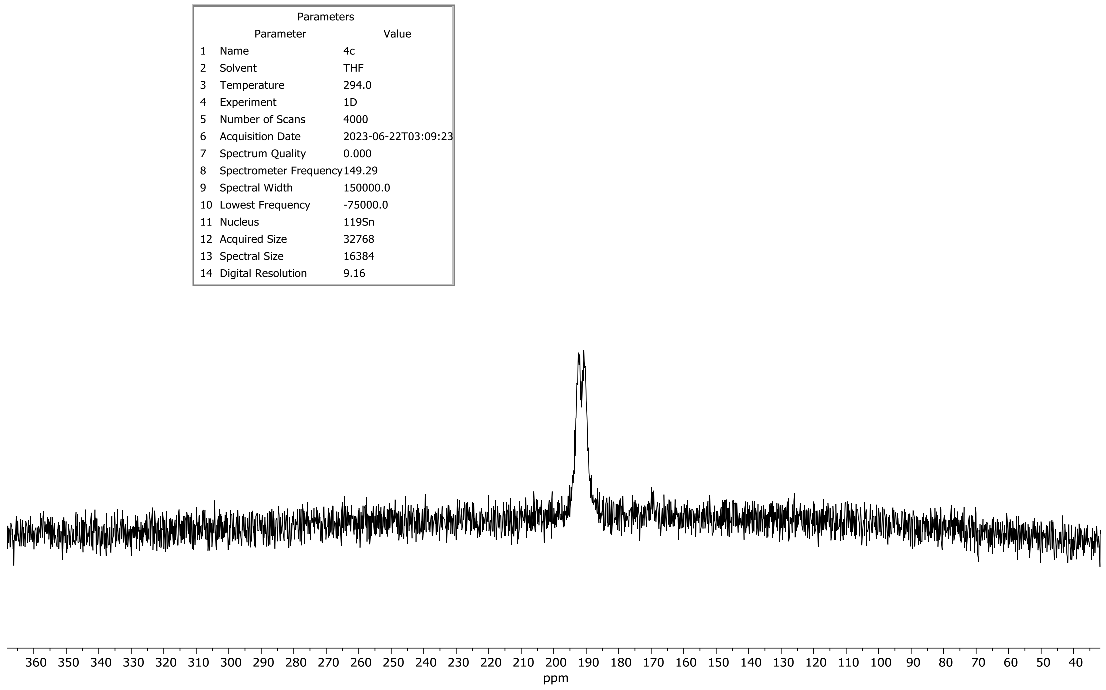

Figure S20: $^{119}Sn\{^1H\}$ NMR spectrum of compound **4c** in THF.

NMR spectra of compound 4d

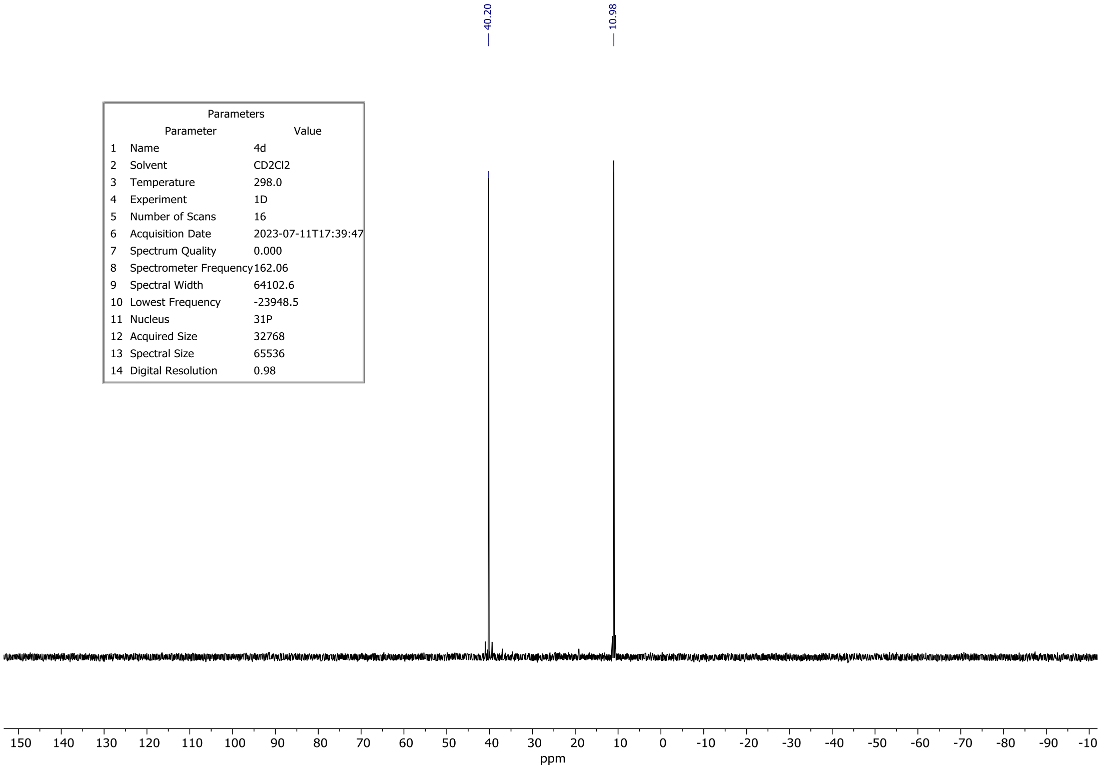

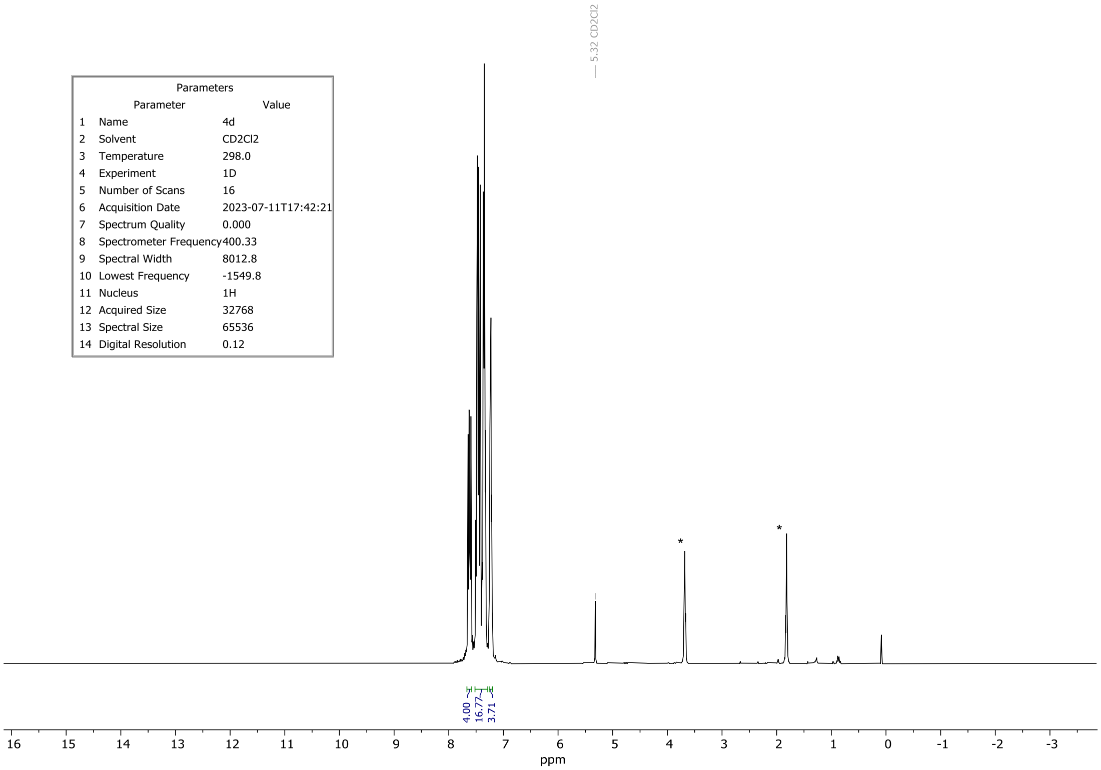

Figure S21: $^{31}P\{^{1}H\}$ NMR and ^{1}H NMR spectrum of compound **4d** in CD_2Cl_2. ^{1}H NMR spectrum shows residues of THF (*) and small amounts of grease.

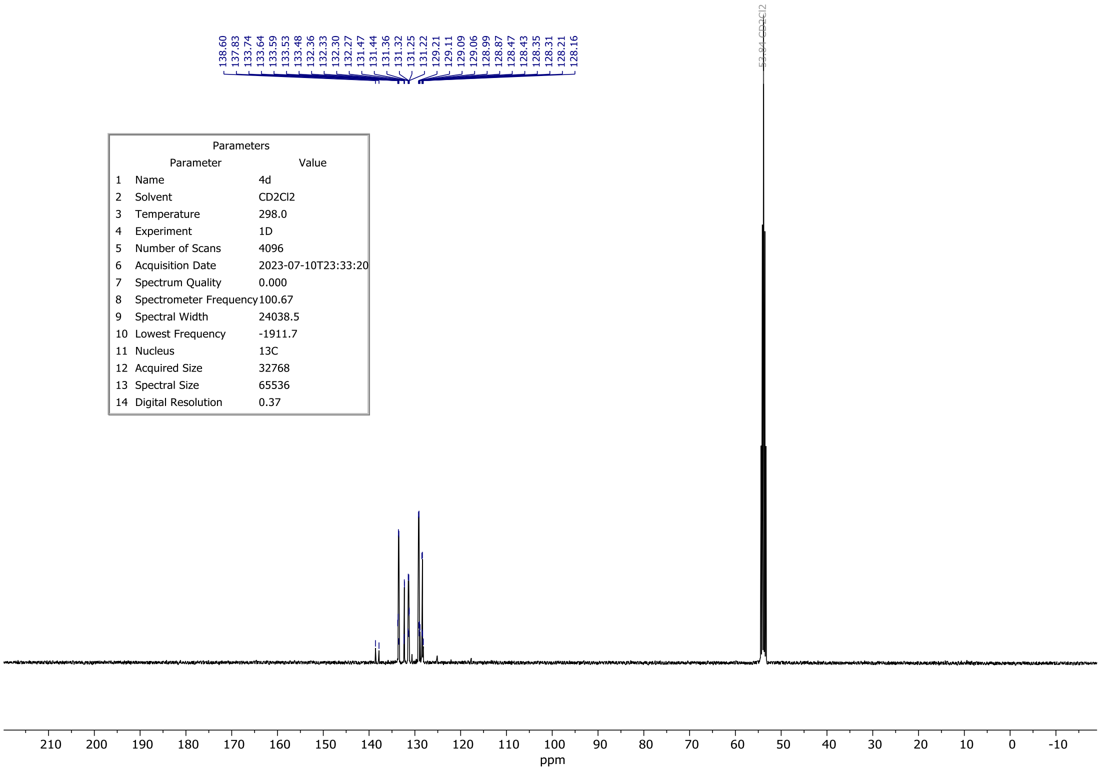

Figure S22: $^{13}C\{^1H\}$ NMR spectrum of compound **4d** in CD_2Cl_2.

Parameters		
	Parameter	Value
1	Name	4d
2	Solvent	CD2Cl2
3	Temperature	298.0
4	Experiment	1D
5	Number of Scans	1500
6	Acquisition Date	2023-07-11T19:37:39
7	Spectrum Quality	0.000
8	Spectrometer Frequency	149.29
9	Spectral Width	150000.0
10	Lowest Frequency	-75000.0
11	Nucleus	119Sn
12	Acquired Size	32768
13	Spectral Size	65536
14	Digital Resolution	2.29

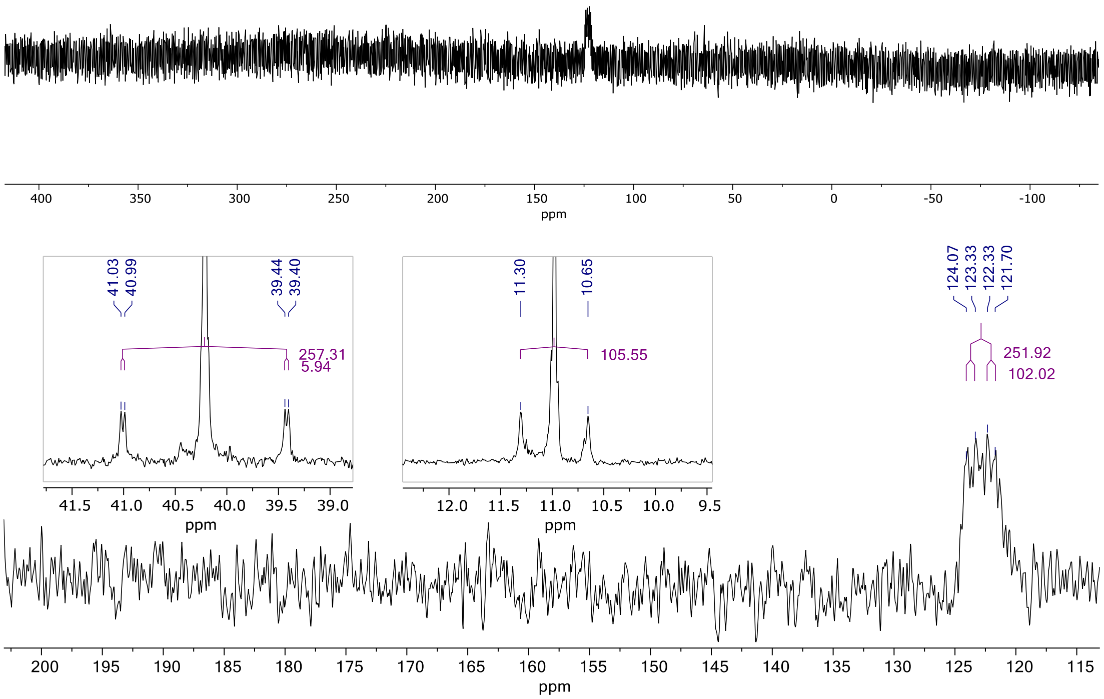

Figure S23: $^{119}Sn\{^{1}H\}$ NMR spectrum of compound **4d** in CD_2Cl_2.

2.4 NMR spectra of compounds 5

NMR spectra of compound 5

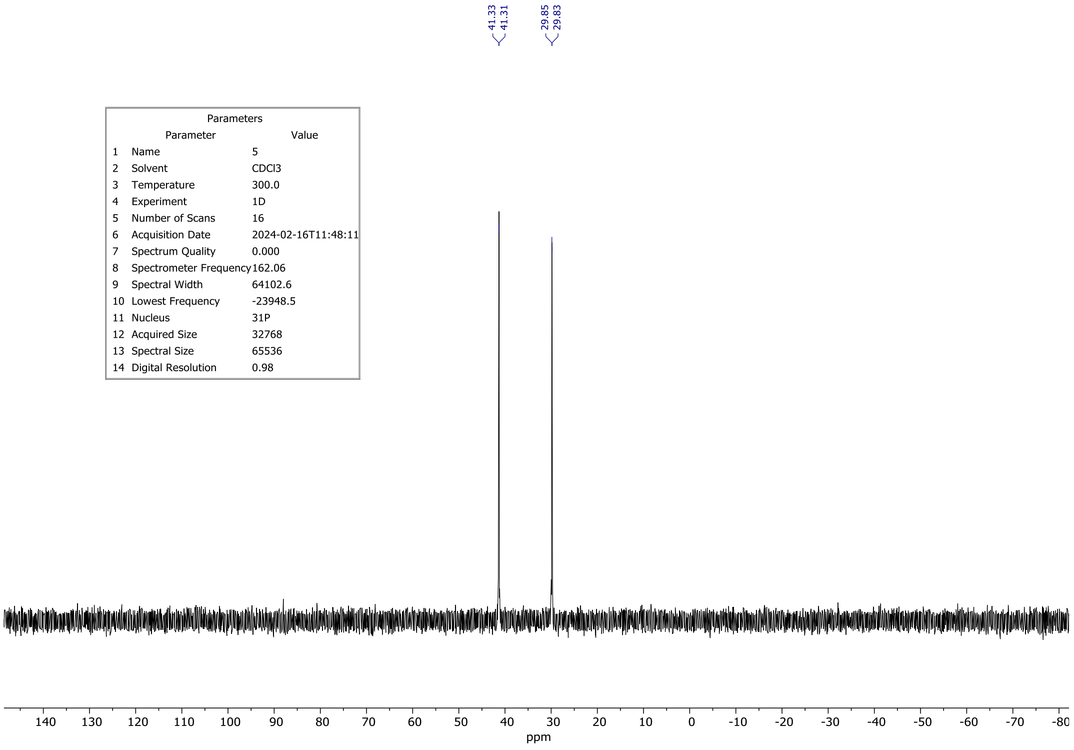

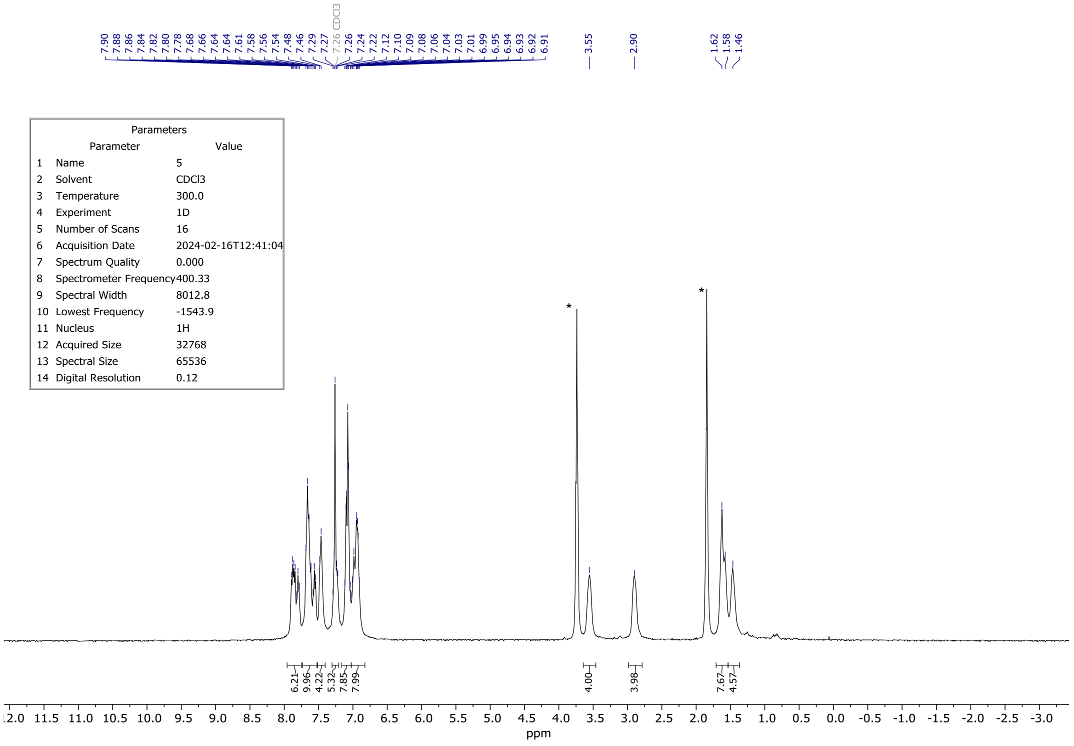

Figure S24: $^{31}P\{^1H\}$ NMR and ^1H NMR spectrum of compound **5** in $CDCl_3$. ^1H NMR spectrum shows residues of THF (*).

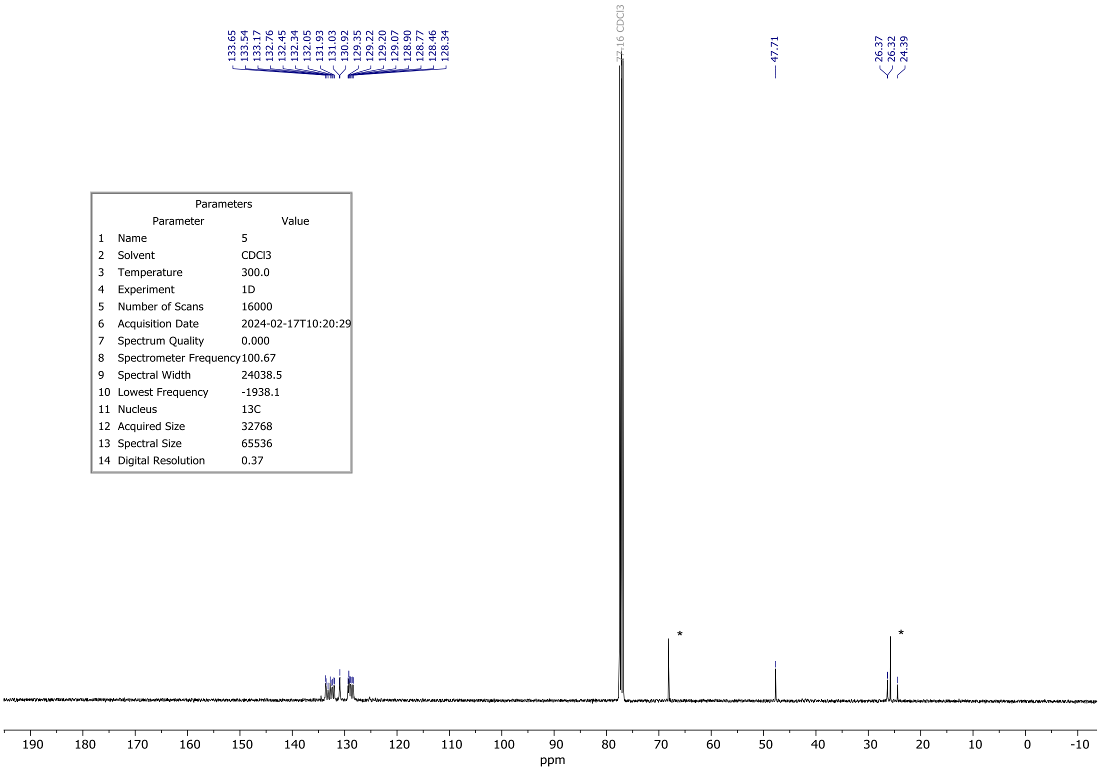

Figure S25: $^{13}C\{^{1}H\}$ NMR spectrum of compound **5** in $CDCl_3$. Spectrum shows residues of THF (*).

NMR spectra of compound 4a-$SnCl_3$

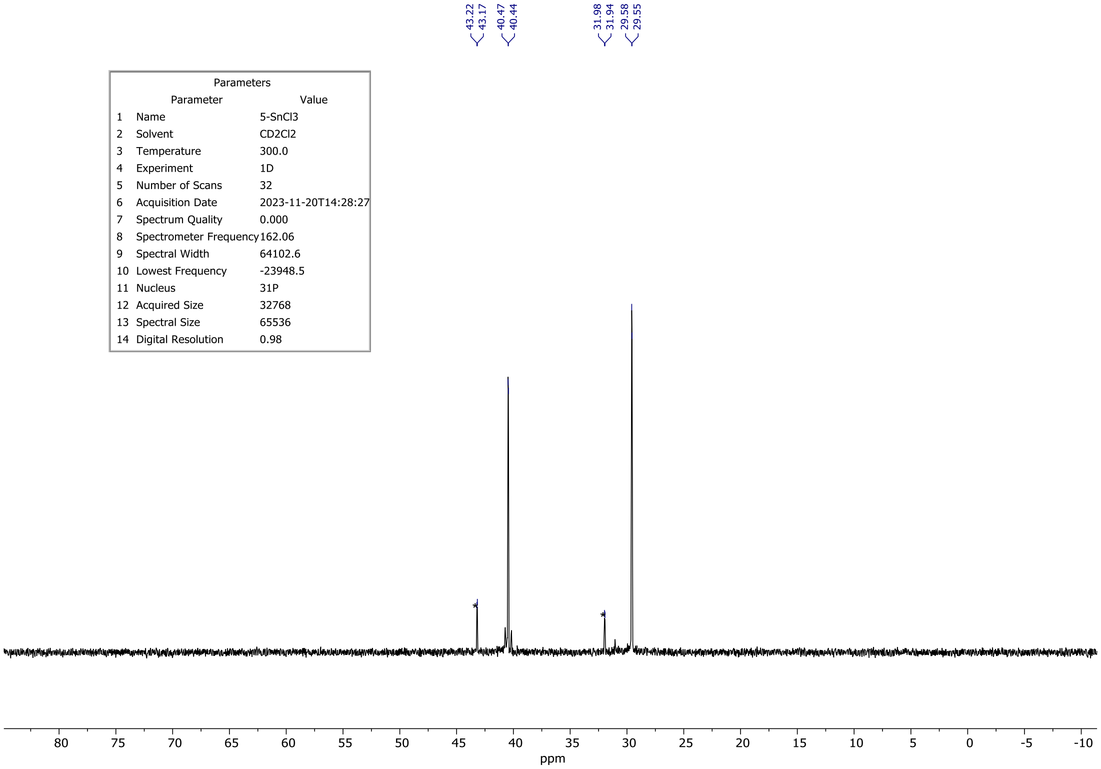

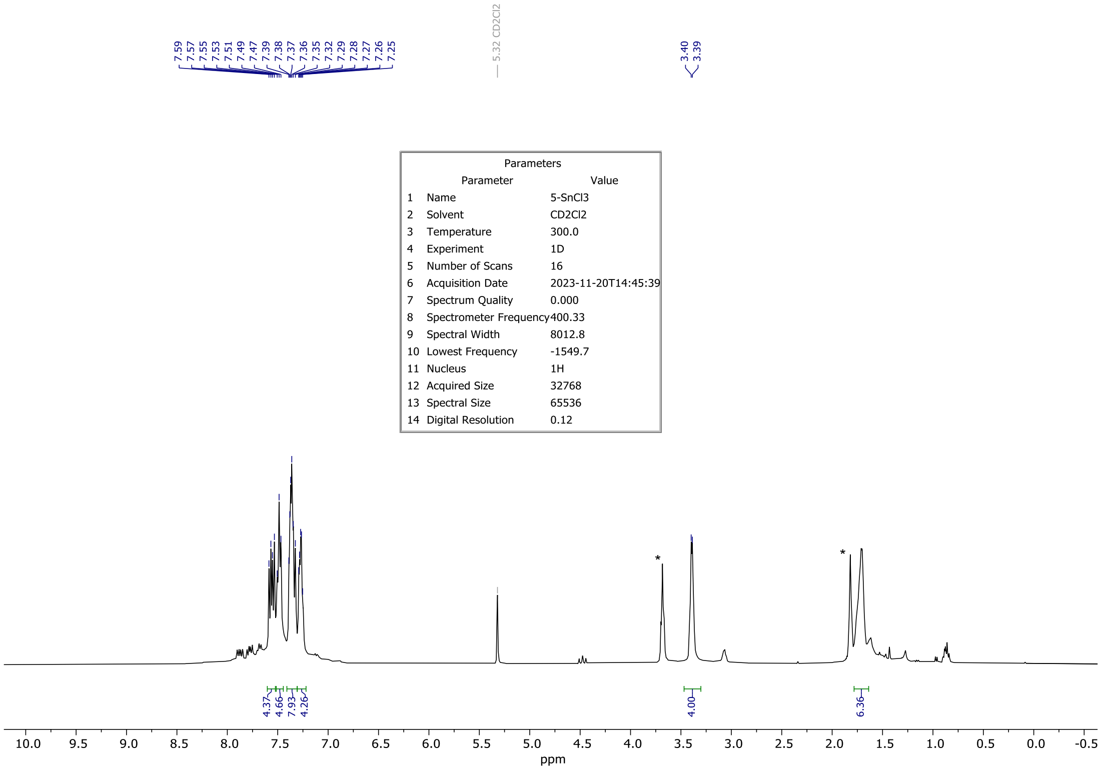

Figure S26: $^{31}P\{^{1}H\}$ NMR and ^{1}H NMR spectrum of compound **4a-$SnCl_3$** in CD_2Cl_2. ^{31}P NMR spectrum shows **6** as byproduct (*).^{1}H NMR spectrum shows residues of THF (*).

2.5 NMR spectra of compounds 6

NMR spectra of compound 6-BF_4

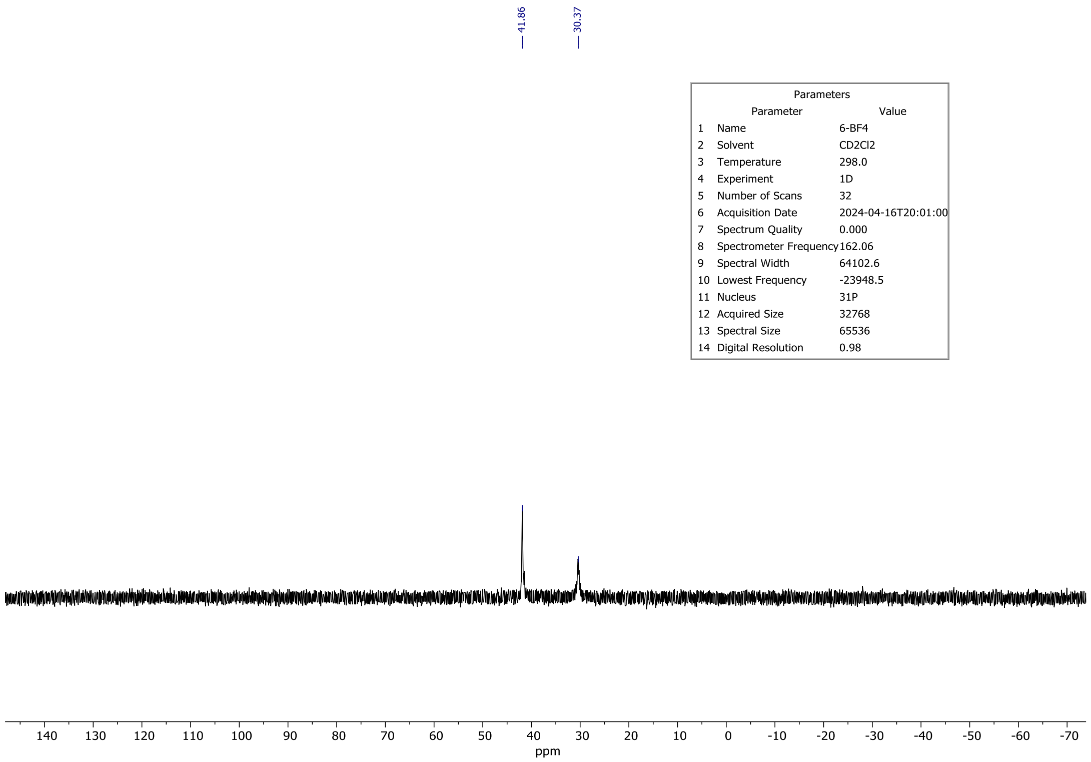

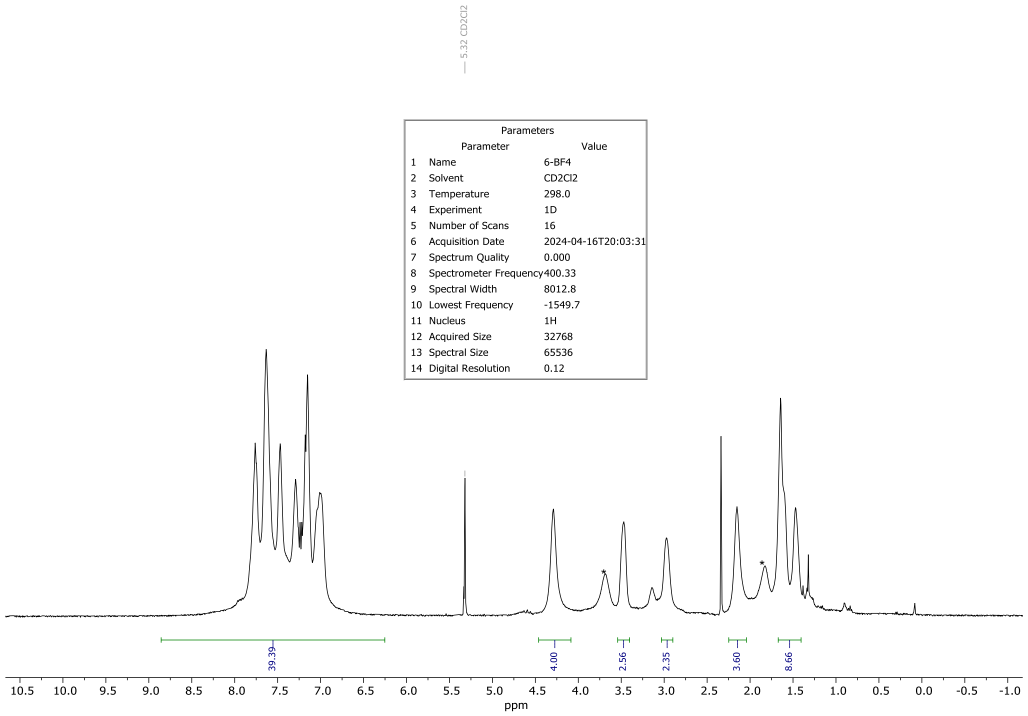

Figure S27: $^{31}P\{^{1}H\}$ NMR and ^{1}H NMR spectrum of compound **6-BF_4** in CD_2Cl_2. ^{1}H NMR spectrum shows residues of THF (*).

NMR spectra of compound 6-$SnCl_6$

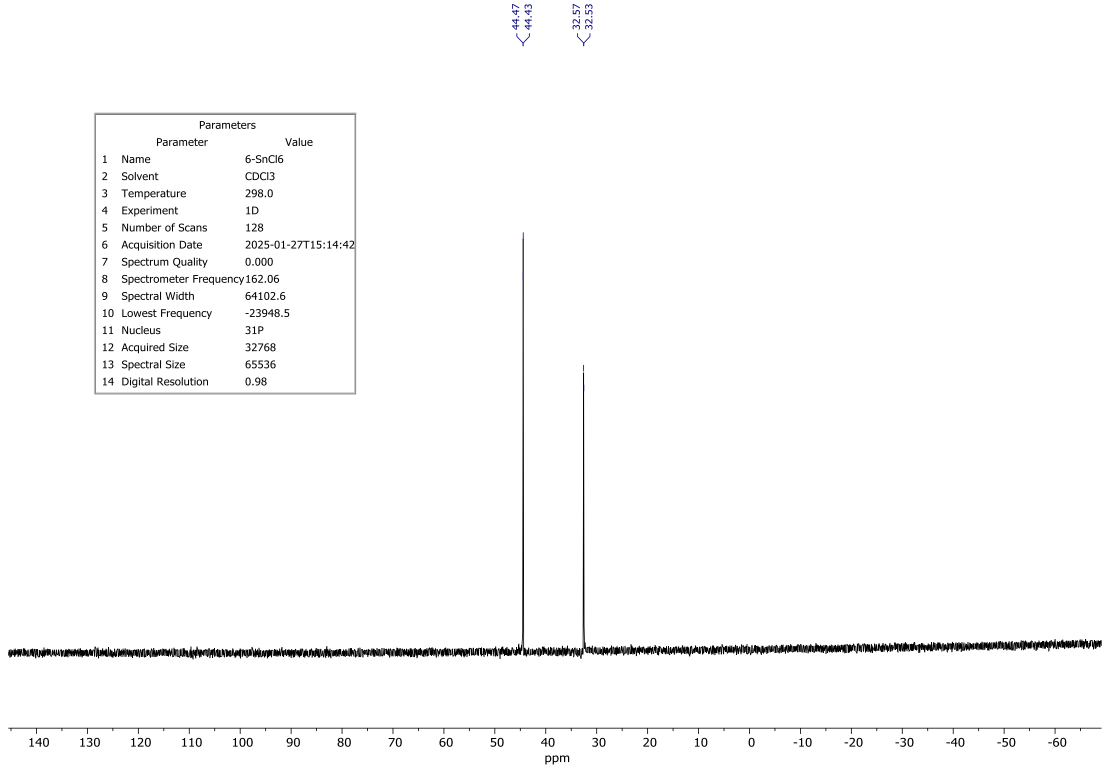

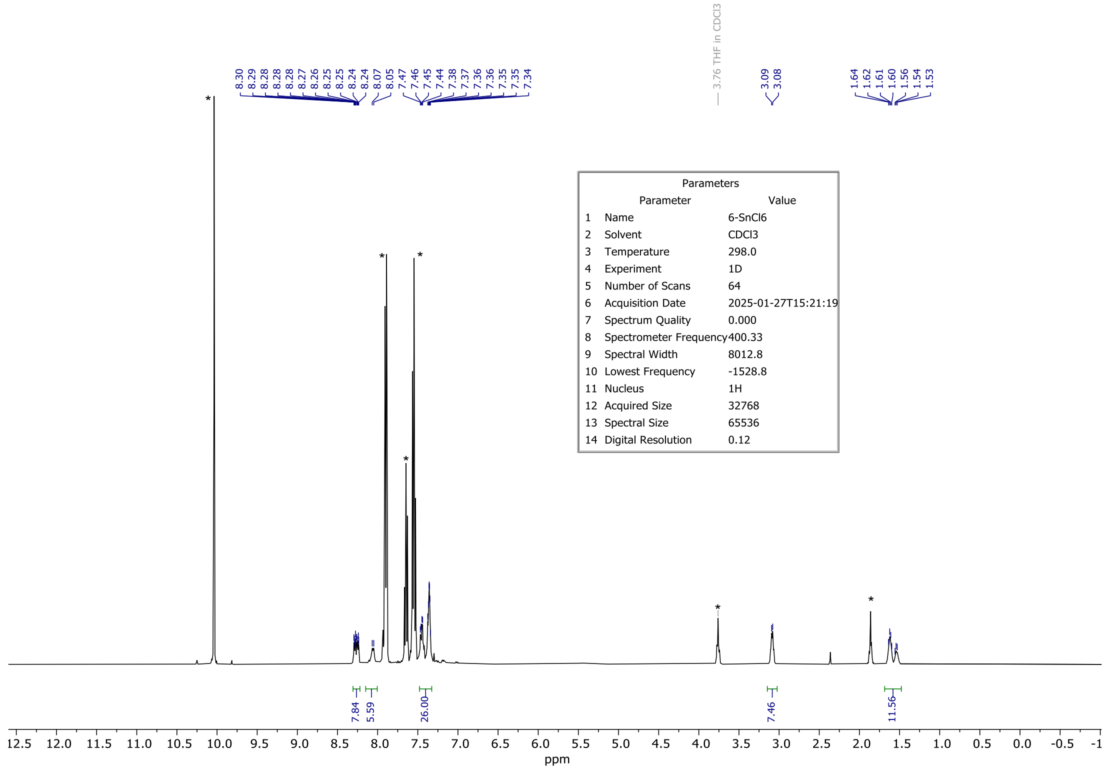

Figure S28: $^{31}P\{^{1}H\}$ NMR and ^{1}H NMR spectrum of compound **6-$SnCl_6$** in $CDCl_3$. ^{1}H NMR spectrum shows residues of THF (*) and benzealdehyde (*).

3. Crystal structure determination.

3.1 General information

Good quality single crystals were hand-picked under polarized optical microscopy and then mounted on the diffractometer. Data collection of the compounds was conducted with an Oxford SuperNova or Rigaku Synergy (For compounds 2, 3 and 4 of chapter 3.4: a Bruker SMART diffractometer with an Apex II area detector (Bruker AXS LLC, USA) using Mo Kα radiation from a sealed source with focusing optics)**.** The structures were solved using dual space FT and direct methods, refined with the Shelx software package and expanded using Fourier techniques.[6] The crystals of all compounds were mounted in an inert oil (perfluoropolyalkylether). Crystal structure determination were affected at 100 K. Crystallographic data of published molecular structures (including structure factors) have been deposited with the Cambridge Crystallographic Data Centre as supplementary publication no. CCDC (listed in the following tables). Copies of the data can be obtained free of charge on application to Cambridge Crystallographic Data Centre, 12 Union Road, Cambridge CB2 1EZ, UK; [fax: (+44) 1223-336-033; email: deposit@ccdc.cam.ac.uk].

Table S2: Data collection and structure refinement details for compounds **2-O** and **2-S**.

**Compound**	**2-O**	**2-S**
Formula	$C_{58}H_{72}N_4O_2P_2Si_4Sn_2$	$C_{58}H_{72}N_4P_2S_2Si_4Sn_2$
CCDC	-	2481154
Formula weight	1268.87	1300.99
Temperature [K]	100(2)	100(2)
Wavelength [Å]	1.54184	1.54184
Crystal system	Monoclinic	Monoclinic
Space group	$P2_1/c$	$P2_1/c$
a [Å]	19.8007(4)	19.8970(3)
b [Å]	17.5658(2)	17.93040(10)
c [Å]	19.0320(4)	19.0955(3)
α[°]	90	90
β [°]	113.996	113.580(2)
γ [°]	90	90
Volume [Å^3]	6047.5(2)	6243.70(17)
Z	4	4
Calc. density [Mg·m^{-3}]	1.394	1.384
μ ($Mo_{K\alpha}$) [mm^{-1}]	8.162	8.504
F(000)	2600	2664
Crystal dimensions [mm]	0.232 x 0.150 x 0.074	0.112 x 0.068 x 0.049
Theta range θ [°]	2.443 to 77.191	3.457 to 67.989
Index ranges	-23<=h<=25	-23 <= h <= 23
	-22<=k<=22	-17 <= k< = 21
	-23<=l<=18	-22 <= l< = 22
Reflections collected	85123	83310
Independent reflections	12369 [R_{int} = 0.0830]	11380 [Rint = 0.0481]
Data/Restraints/Parameter	12369 / 559 / 1060	11380 / 0 / 661
Goodness-of-fit on F^2	1.078	1.016
Final R indices [I>2sigma(I)]	R1 = 0.0667, wR2 = 0.1416	R1 = 0.0256, wR2 = 0.0645
Largest diff. peak and hole [e·Å-3]	1.064 and -0.974	1.052 and -0.586

Table S3: Data collection and structure refinement details for compounds **4b+N$_2$O**, **4c+N$_2$O** and 2-Se.

**Compound**	**4a-SnCl$_3$**	**4c+N$_2$O**	**5**
Formula	$C_{30}H_{30}Cl_3NP_2SSn$	$C_{31}H_{43}Cl_3P_2SSn$	$C_{60}H_{60}Cl_2N_2OP_4S_2Sn_2$
CCDC	2481150	2481151	2481155
Formula weight	723.59	734.69	1321.38
Temperature [K]	100(2)	105(2)	100(2)
Wave lenght [Å]	1.54184	1.54184	1.54184
Crystal system	Monoclinic	monoclinic	monoclinic
Space group	$P2_1/c$	$P2_1/c$	$C2_1/c$
a [Å]	9.23420(10)	9.9416(3)	28.0096(3)
b [Å]	31.6515(4)	20.0598(3)	12.29950(10)
c [Å]	10.31760(10)	19.7546(5)	18.7039(2)
α[°]	90	90	90
β [°]	99.7860(10)	124.943(4)	94.7420(10)
γ [°]	90	90	90
Volumen [Å^3]	2971.71(6)	3229.37(19)	6421.51(11)
Z	4	4	4
Calc. density [Mg·m^{-3}]	1.617	1.511	1.367
μ ($Mo_{K\alpha}$) [mm^{-1}]	11.164	10.264	8.797
F(000)	1456	1504	2672
Crystal dimensions [mm]	0.809 x 0.379 x 0.125	0.100 x 0.060 x 0.030	0.223 x 0.185 x 0.046
Theta range θ [°]	2.792 to 67.043	3.508 to 67.997	3.166 to 67.999
Index ranges	-9 ≤ h ≤ 11	-11 ≤ h ≤ 11	-32 ≤ h ≤ 33
	-37 ≤ k ≤ 37	-24 ≤ k ≤ 24	-14 ≤ k ≤ 14
	-12 ≤ l ≤ 11	-23 ≤ l ≤ 19	-21 ≤ l ≤ 22
Reflections collected	37275	42002	20768
Independent reflections	5298 [R(int) = 0.0963]	5861 [R(int) = 0.0941]	5816 [R(int) = 0.0298]
Data/Restraints/Parameter	5298/0/343	5861/0/343	5816/0/349
Goodness-of-fit on F^2	1.046	1.062	1.046
Final R indices [I>2sigma(I)]	R1 = 0.0522, wR2 = 0.1353	R1 = 0.0386, wR2 = 0.0955	R1 = 0.0341, wR2 = 0.0917
Largest diff. peak and hole [e·Å-3]	2.231 and -1.892	1.243 and -1.612	0.599 and -1.053

Table S4: Data collection and structure refinement details for compounds **6-BF$_4$** and **6-SnCl$_6$**.

**Compound**	**6-BF$_4$**	**6-SnCl$_6$**
Formula	$C_{61}H_{62}BCl_3F_4N_2P_4S_2Sn$	$C_{122}H_{124}Cl_{12}N_4P_8S_4Sn_3$
CCDC	2481153	2481152
Formula weight	1322.97	2803.71
Temperature [K]	100(2)	100(2)
Wave lenght [Å]	1.54184	1.54184
Crystal system	monoclinic	triclinic
Space group	$P2_1/c$	P-1
a [Å]	19.76730(10)	11.0011(5)
b [Å]	15.74790(10)	14.2240(7)
c [Å]	19.82460(10)	21.9070(7)
α[°]	90	102.299(3)
β [°]	91.6490(10)	94.481(2)
γ [°]	90	110.011(4)
Volumen [Å^3]	6168.71(6)	3104.2(2)
Z	4	1
Calc. density [Mg·m^{-3}]	1.425	1.500
μ ($Mo_{K\alpha}$) [mm^{-1}]	6.529	9.128
F(000)	2704	1422
Crystal dimensions [mm]	0.320 x 0.250 x 0.230	0.163 x 0.107 x 0.039
Theta range θ [°]	3.585 to 76.757	3.421 to 76.704
Index ranges	-24 ≤ h ≤ 24	-13 ≤ h ≤ 10
	-19 ≤ k ≤ 15	-17 ≤ k ≤ 17
	-24 ≤ l ≤ 24	-26 ≤ l ≤ 27
Reflections collected	86526	13288
Independent reflections	12728 [R(int) = 0.0408]	13288 [R(int) = 0.0614]
Data/Restraints/Parameter	12728/40/718	13288/0/692
Goodness-of-fit on F^2	1.036	1.045
Final R indices [I>2sigma(I)]	R1 = 0.0402, wR2 = 0.1021	R1 = 0.0622, wR2 = 0.1646
Largest diff. peak and hole [e·Å-3]	1.884 and -1.933	3.913 and -1.882

3.2 ORTEP plots of all crystal structures

Ortep plot of 2-O

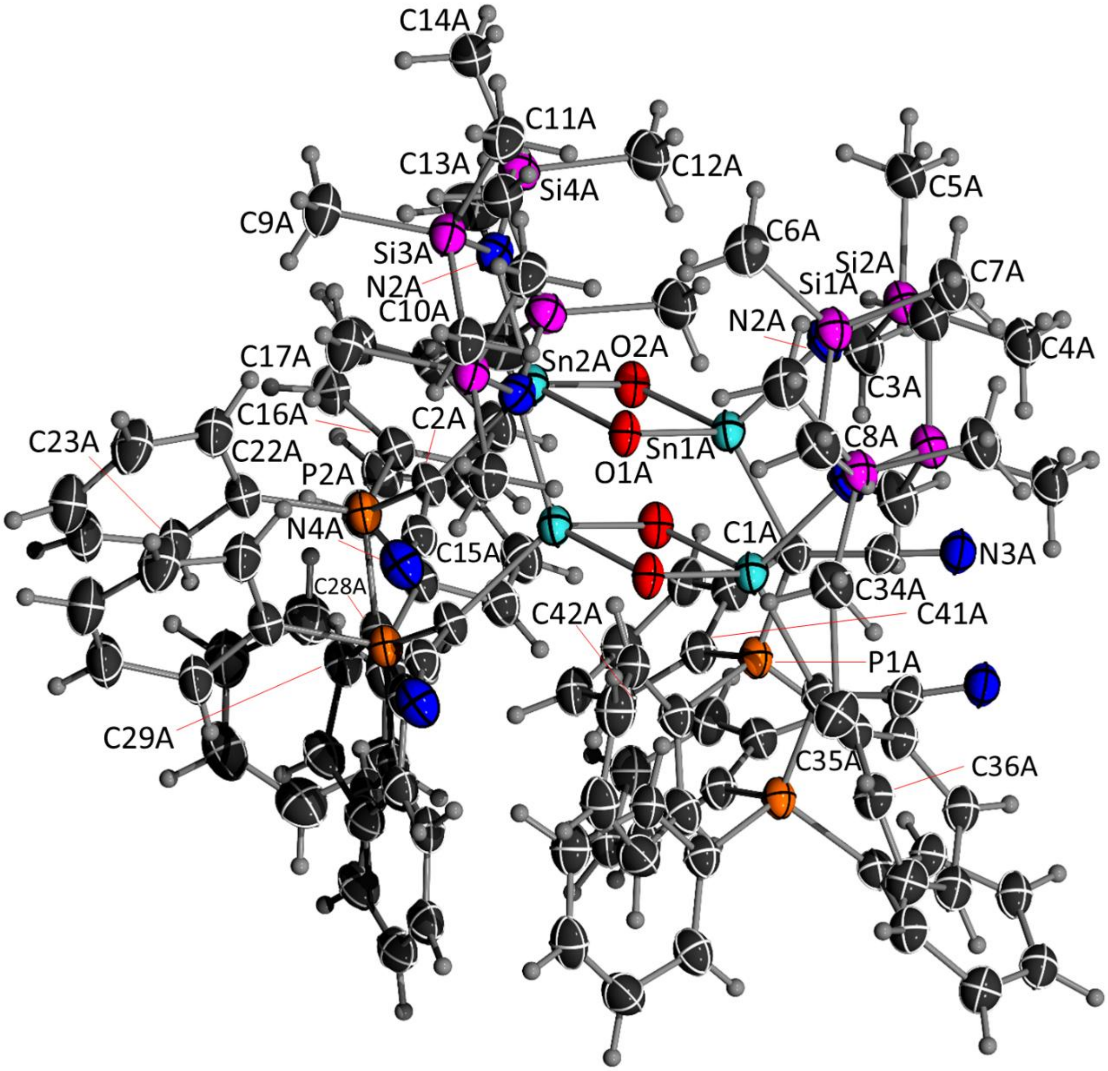

Figure S29: Molecular structure of compound **2-O**. Thermal ellipsoids at 50% probability level. Due to the complete disorder in the crystal, only one part (Part A) is highlighted. Selected bond lengths [Å] and angles [°]: P1A-C1A 1.701(18), P2A-C2A 1.707(18), C1A-C34A 1.42(2), C2A-C15A 1.40(2), C34A-N3A 1.15(2), C15A-N4A 1.16(2) Sn1A-C1A 2.078(17), Sn2A-C2A 2.097(17), Sn1-N1A 2.044(17), Sn2A-N2A 2.042(17), Sn1A-O1A 2.014(18), Sn1A-O2A 2.008(18), Sn2A-O1A 1.992(18), Sn2-O2A 1.995(18), P1A-C1A-C34A 115(2), P2A-C2A-C15A 118(2), C1A-C34A-N3A 171(10), C2A-C15A-N4A 168(10), Sn1A-O1A-Sn2A 95.3(9), Sn1A-O2A-Sn2A 95.4(9).

Ortep plot of 2a-S

Additional information regarding the structure refinement: All hydrogen atoms were placed on ideal positions.

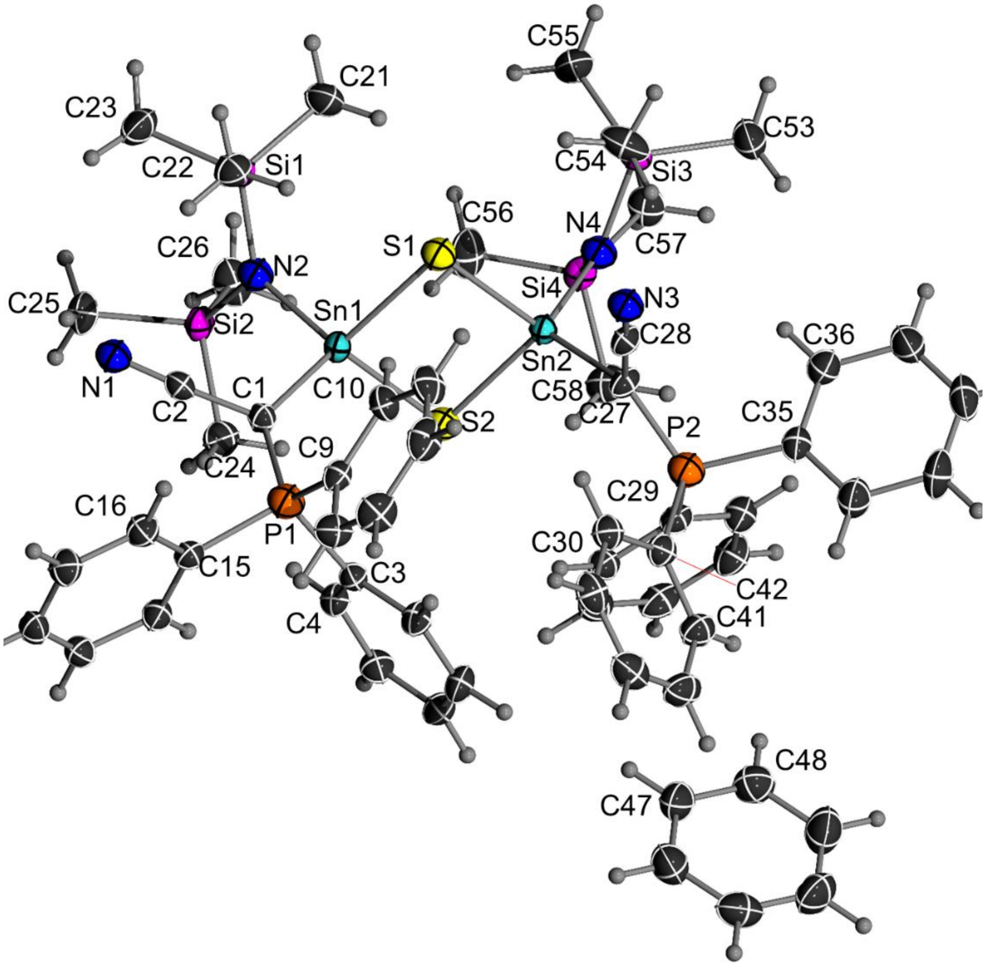

Figure S30: Molecular structure of compound **2-S**. Thermal ellipsoids at 50% probability level. Due to the complete disorder in the crystal, only one part (Part A) is highlighted. Selected bond lengths [Å] and angles [°]: P1-C1 1.701(2), P2-C27 1.705(2), C2-C1 1.401(3), C27-C28 1.405(3), C2-N1 1.163(3), C28-N3 1.164(3) Sn1-C1 2.089(2), Sn2-C27 2.105(2), Sn1-N2 2.042(2), Sn1-N4 2.056(2), Sn1-S1 2.421(6), Sn1-S1 2.424(5), Sn2-S1 2.410(5), Sn2-S2 2.419(5), P1-C1-C2 117.6(2), P2-C27-C28 116.5(2), C1-C2-N1 175.3(2), C21-C22-N2 179.4(3), Sn1-S1-Sn2 86.1(2), Sn1-S2-Sn2 85.9(2).

Ortep plot of ylide-substituted $SnCl_3$ obtained from 4c and N_2O

Additional information regarding the structure refinement: All hydrogen atoms were placed on ideal positions.

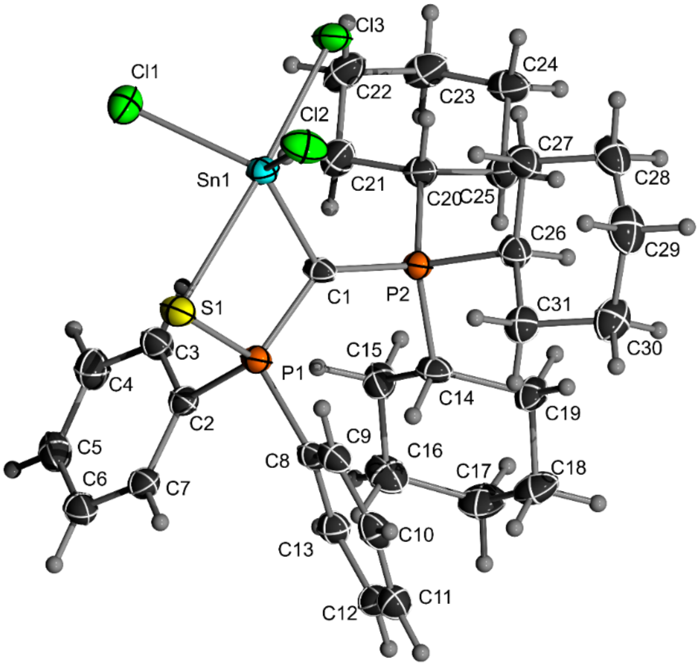

Figure S31: Molecular structure of compound **4c+N_2O**. Thermal ellipsoids at 50% probability level. Selected bond lengths [Å] and angles [°]: P1–C1 1.740(3), P2–C1 1.741(3), Sn1–C1 2.117(3), P2–C1–P1 130.59(19).

ORTEP plot of 5

Additional information regarding the structure refinement: All hydrogen atoms were placed on ideal positions. The disorder of the O-$SnCl_2$ moiety on a symmetry center was modeled using the PART-1 instruction and setting the occupancy to 50%. A disordered solvent molecule was removed using the squeeze function of PLATON and the ABIN instruction.

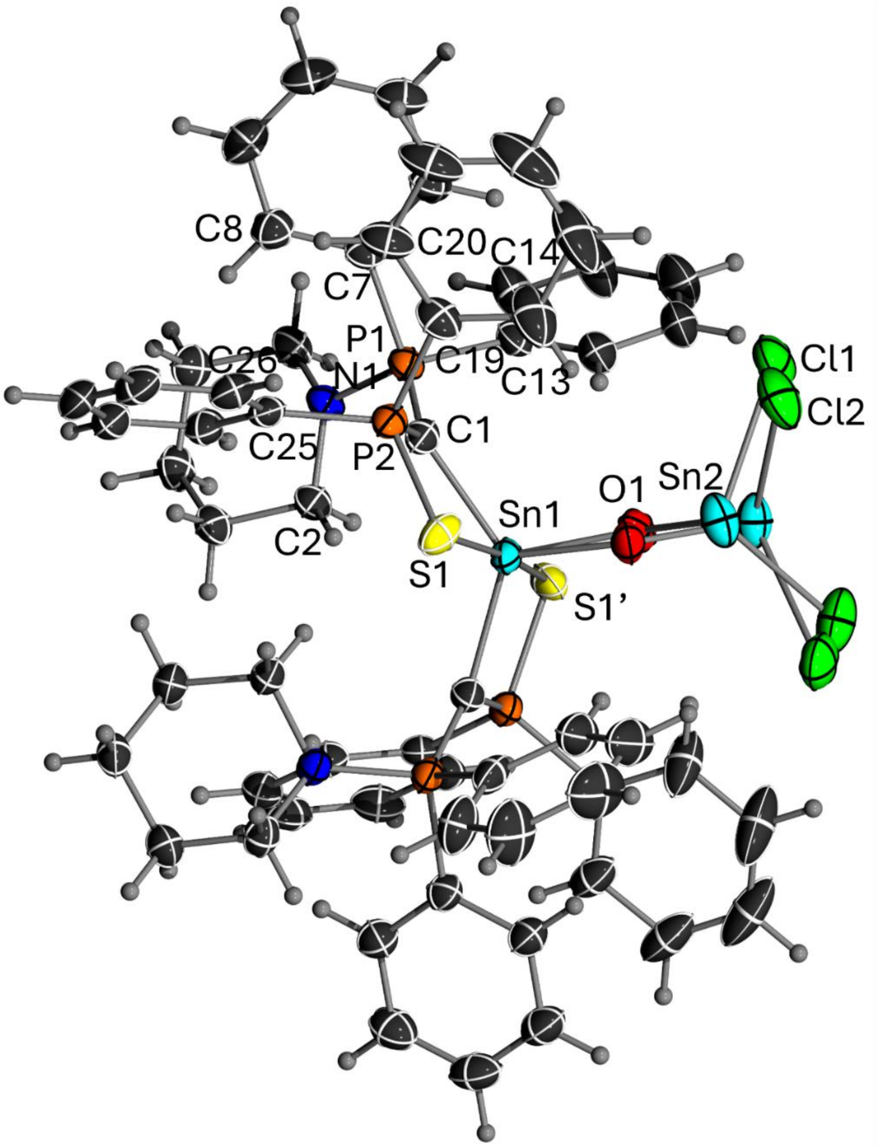

Figure S32: Molecular structure of compound **5**. Thermal ellipsoids at 50% probability level. Selected bond lengths [Å] and angles [°]: Sn1–O1 1.954(3), O1–Sn2 1.984(4), C1–Sn1 2.126(3), C1–P1 1.700(3), C1–P2 1.723(3), Sn1–O1–Sn2 134.9(2), P–C–P 128.5(2).

ORTEP plot of 4a-SnCl$_3$

Additional information regarding the structure refinement: All hydrogen atoms were placed on ideal positions.

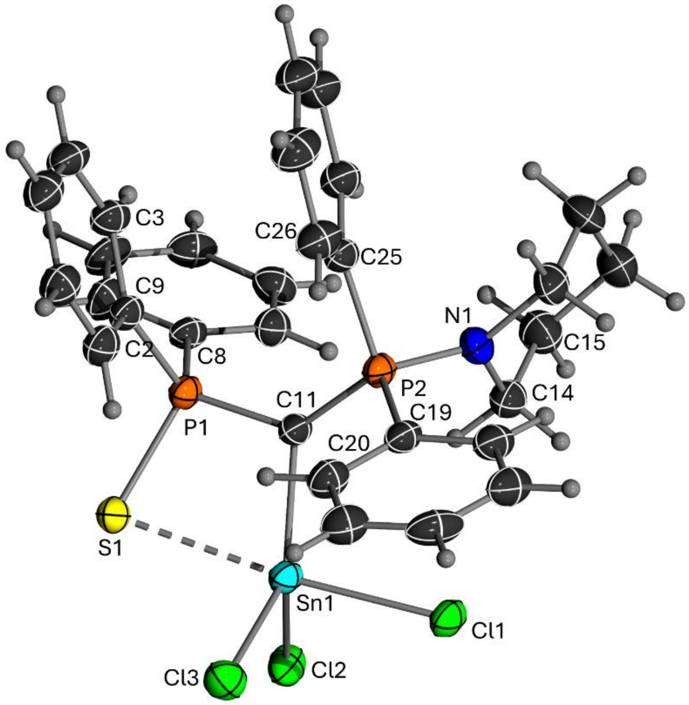

Figure S33: Molecular structure of compound **4a-SnCl$_3$**. Thermal ellipsoids at 50% probability level. Selected bond lengths [Å] and angles [°]: C1–Sn1 2.118(5), C1–P1 1.698(5), C1–P2 1.729(5), P–C–P 131.6(3).

ORTEP plot of 6-BF_4

Additional information regarding the structure refinement: All hydrogen atoms were placed on ideal positions. The structure has been solved as a two component twin using the MERG and BASF instructions refining to a ratio of 60% and 40% (twin law: 0.9895 -0.0192 0.0008 0.0373 1.0074 -0.0061 -0.0094 0.0158 1.0023). A disordered solvent molecule (DCM) was modelled using the EADP restraint and refined using the PART instructions and free variable, which optimized to occupancies of 66% and 34%. The disordered BF_4 moiety was modelled using the DFIX, EADP and DELU restraints and refined using the PART instructions and free variable, which optimized to occupancies of 53% and 47%. A disordered solvent molecule was removed using the squeeze function of PLATON and the ABIN instruction.

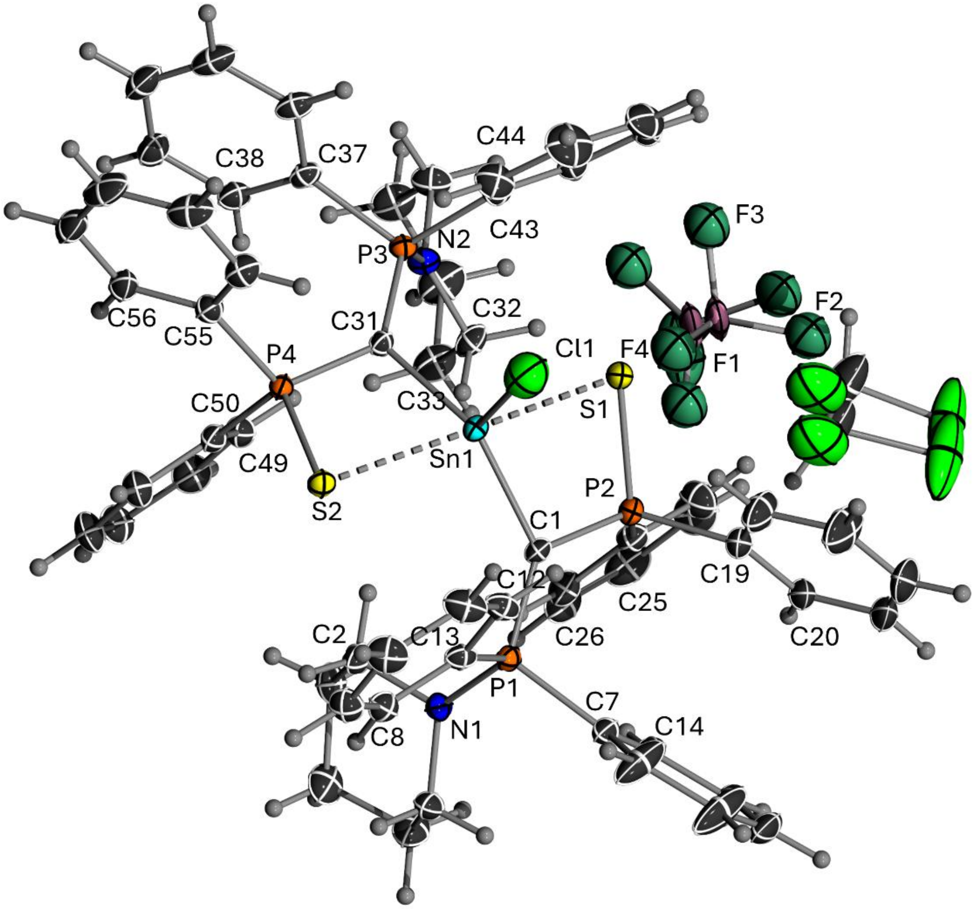

Figure S34: Molecular structure of compound **6-BF_4**. Thermal ellipsoids at 50% probability level. Selected bond lengths [Å] and angles [°]: C1–Sn1 2.116(2), C1–P1 1.710(3), C1–P2 1.729(3), P–C–P 129.87(15).

ORTEP plot of 6-SnCl$_6$

Additional information regarding the structure refinement: All hydrogen atoms were placed on ideal positions.

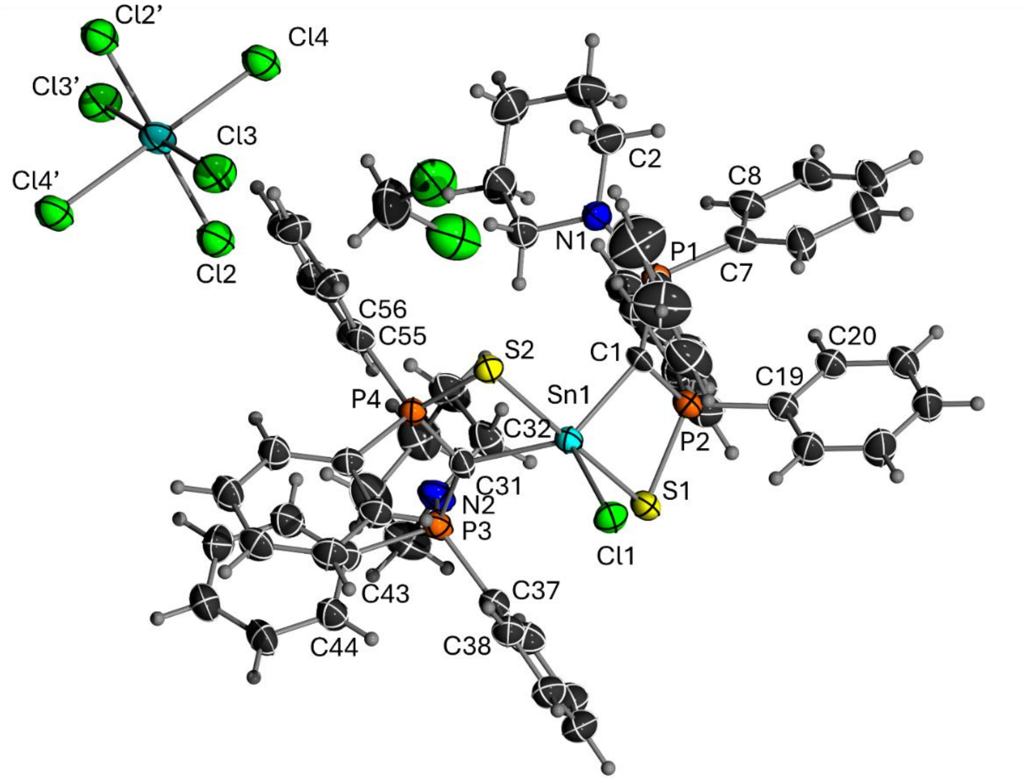

Figure S35: Molecular structure of compound **6-SnCl$_6$.** Thermal ellipsoids at 50% probability level. Selected bond lengths [Å] and angles [°]: Sn1–Cl1 2.373(1), C1–Sn1 2.108(5), C1–P1 1.711(6), C1–P2 1.721(6), P–C–P 129.6(3).

4. DFT

4.1 General remarks

All calculations were performed without symmetry restrictions. Starting coordinates were obtained with GaussView 6.1[7] or directly from the crystal structure analyses if available. The geometry optimizations were carried out with the ORCA 5.0.4/6.0.1[8] program package. The geometry optimizations were performed using Density Functional Theory (DFT)[9] with the B86[10] functional in combination with the def2svp[11] basis set and with Grimme's D3 dispersion correction[12] with Becke-Johnson damping[13]. Harmonic vibrational frequency analyses were performed at the same level of theory as the optimizations to determine the nature of the structure. The vibrational frequency analysis showed no imaginary frequencies. Single point energies were calculated using the def2tzvpp[11] basis set. The NBO analysis was performed with NBO Version 7.0[14] using the def2tzvpp basis set. The optimized structures were used for the quantum theory of atoms in molecules (QTAIM)[15] analysis using Multiwfn[16] to visualize the topological properties of the molecules. The results were visualized using the VMD 1.9.4a51[17], GaussView6.1[7] and CYLView softwares.[18]

Optimization of stannanone **5**

As the disorder in the crystal structure of **5** could be solved in two possible ways - one with an angle of the Sn-O-Sn linkage of 134° (**5)** and one with an angle of 166° (**5^{166}**) – we run calculations starting from both possible geometries. While energy optimization lead to two slightly different geometries, which mostly differed in the Sn-O-Sn angle, sturcutre 5 with the more acute angle was found to be thermodynamically more stable. A comparison of the structural data with the experimental data are given in Table S5, further results are shown below. Despite the slightly different structures, no significant differences in bonding situation (charges, orbitals) were observed. Initial geometry optimization of **5** resulted in small imaginary frequency due to numerical noise. To circumvent this a higher grid defgrid3 was used.

Table S5. Comparison of experimental data and computational data on bond lengths (Å) and angles (°).

	**5** (crystal)	**5** (calc)	**5^{166}** (calc)
Sn1-O1	1.954(3)	1.956	1.953
O1-Sn2	1.984(4)	2.103	2.065
C1-Sn1	2.126(3)	2.141	2.156
C1-P1	1.700(3)	1.706	1.705
C1-P2	1.723(3)	1.737	1.737
Sn1-O1-Sn2	134.9(2)	126.3	151.6
P1-C1-P2	128.5(2)	128.6	129.4

4.2 Molecular Orbitals

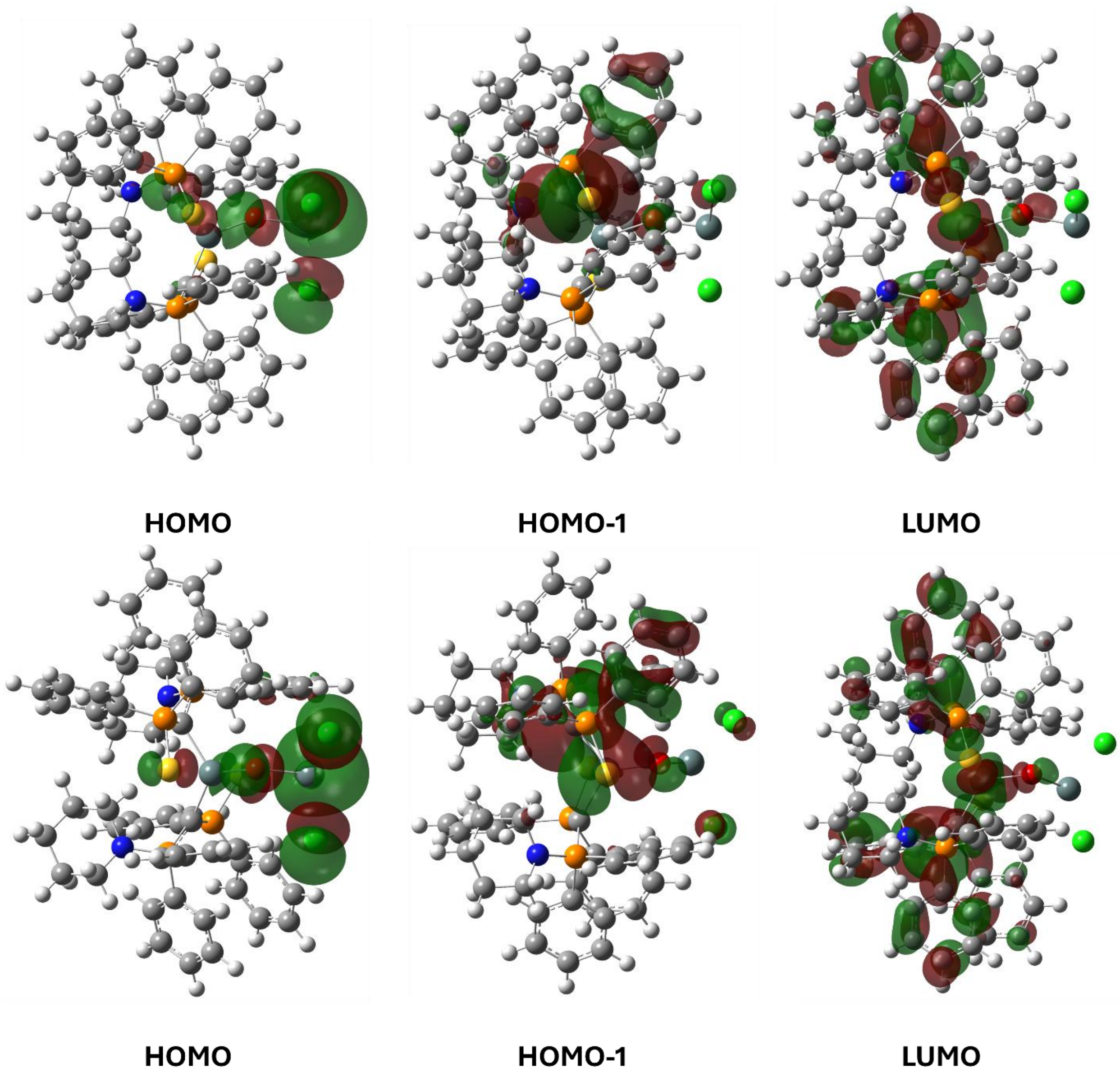

Figure S36. Frontier molecular orbitals of **5**[166] (top) and **5** (bottom).

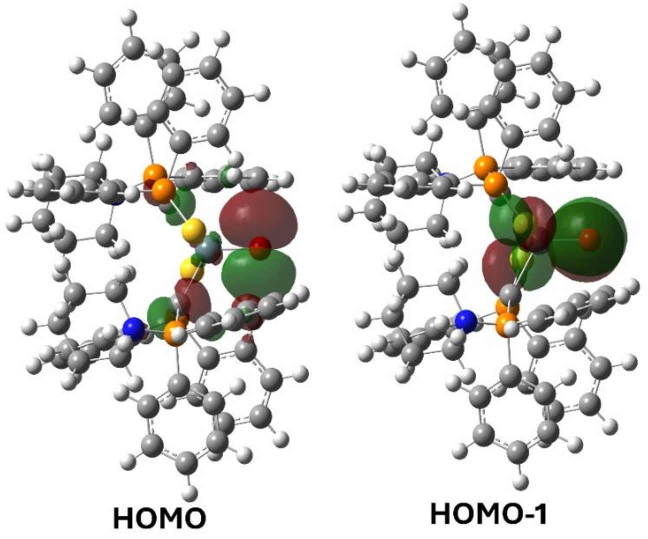

Figure S37. Frontier molecular orbitals of **5’**.

4.3 QTAIM bonding Analysis

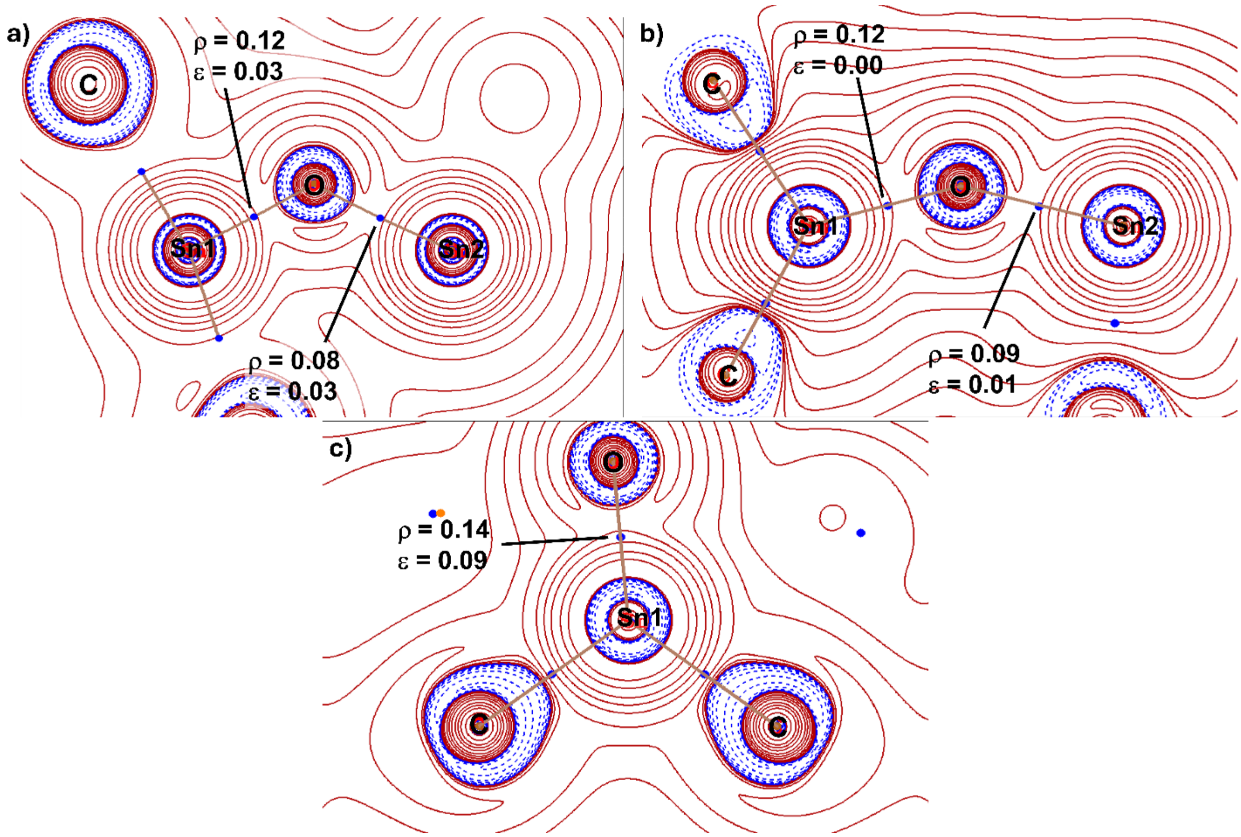

Figure S38. Topology analysis of **5** (a), **5**[166] (b), and **5’** (c). BCPs are indicated by blue dots. Electron density ρ and ellipticity ε are indicated for relevant BCPs.

4.4 NBO Analysis

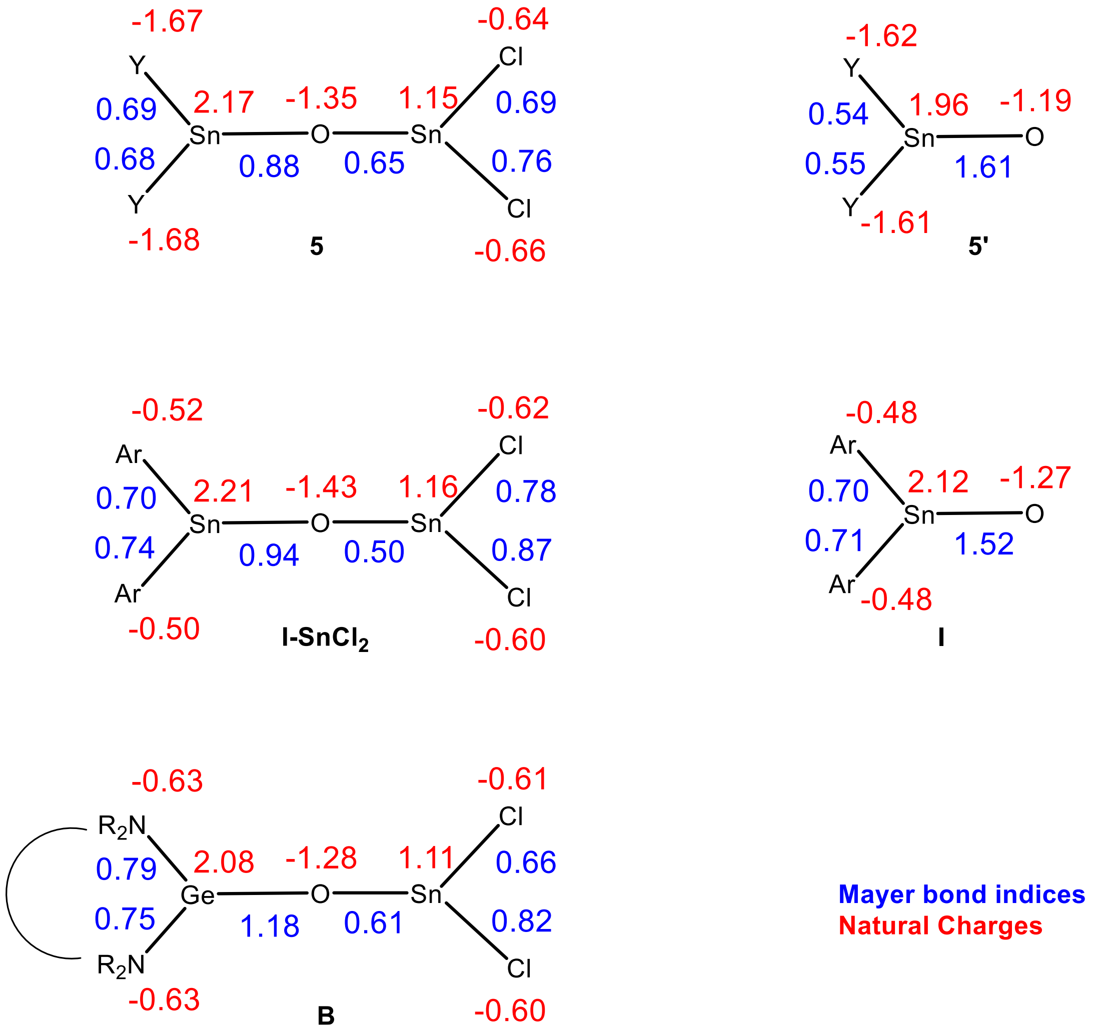

Figure S39. Mayer bond indices and natural charges obtained from NBO analysis.

Table S6. Second order perturbation theory analysis of **5**.

Donor (L) NBO	Acceptor (NL) NBO	*E*(2) (kcal/mol)	E(NL)-E(L) (a.u.)	F(L,NL) (a.u.)
within unit 1				
LP (3) S 1	LV (1)Sn 3	47.16	0.27	0.101
LP (3) S 7	LV (1)Sn 3	46.45	0.27	0.101
From unit 2 to unit 1				

LP (1) O 8	LV (2)Sn 3	26.68	0.59	0.112
LP (4) O 8	LV (2)Sn 3	48.88	0.43	0.130
LP (4) O 8	BD*(1)Sn 3- C 4	14.48	0.41	0.069
LP (4) O 8	BD*(1)Sn 3- C 9	12.44	0.40	0.063
From unit 2 to unit 3				
LP (3) O 8	LV (1)Sn 16	20.08	0.36	0.076
LP (3) O 8	LV (3)Sn 16	52.11	0.39	0.127

4.6 Coordinates of optimized structures

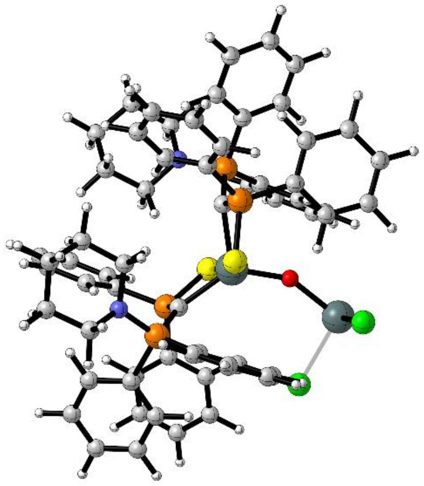	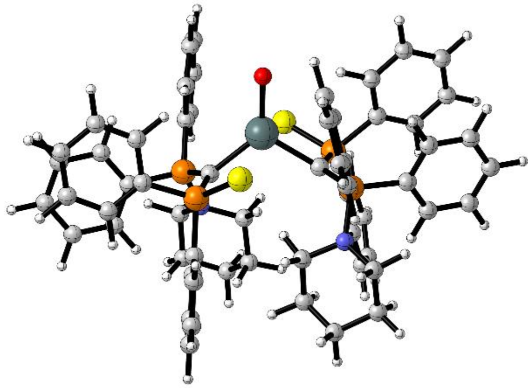
**5**[166]	**5’**
E = -6019.412723	E = -4884.078303
S 11.37607891825187 9.31188280791583 16.27377912447439<br>P 10.10716961802836 8.70795241611773 14.78273414979823<br>Sn 12.80540919934858 9.76740293239611 14.00479163697642<br>C 11.15003927046145 8.52286570157612 13.40628151999732<br>C 8.81214119496253 9.97757855592418 14.54176436061445<br>C 9.28487025735117 7.14295309591139 15.24471420693490<br>S 14.34482772098482 9.51971652531698 11.74943597778693<br>O 12.32309313661004 11.63486523855058 13.69960713889841	S 11.28653995284863 9.40010642270674 16.29390740701253<br>P 10.21523261225466 8.61278159300392 14.75406700719875<br>Sn 12.81444464232279 10.05301662128381 13.99230306607244<br>C 11.20929038374039 8.73696152562796 13.34389287036164<br>C 8.66194546194065 9.56530251003065 14.54772734760640<br>C 9.71044600634171 6.89825410393937 15.16710554012712<br>S 14.41446614879225 9.57430414070708 11.68006109909668<br>O 12.57516961626759 11.93595584223817 13.95924927486678

C	14.60216208016620	8.80646418415838	14.64058527527760
P	10.74749430498226	8.25473380703115	11.77138871213726
C	7.55550110613340	9.67891392612281	13.97562701443841
C	9.17740007355509	11.31889458364201	14.79413910800602
C	8.11807958531050	7.11327177050720	16.03260428672316
C	9.90128891727990	5.93739374780716	14.85417847706012
P	15.55119365806145	8.70311397136829	13.18820741264430
Sn	12.72067610574284	13.65684797118814	13.82619546438404
P	15.02265372062965	8.40957214178820	16.24715673764693
N	11.50535045329707	6.85849630093448	11.17994662931796
C	8.96700424677175	7.93860976787729	11.50314752889974
C	11.16954646319072	9.71043535098814	10.75983480959721
H	7.26827329241512	8.63735242214009	13.76945559957503
C	6.67034390836271	10.72342684373262	13.66270875132955
C	8.29100927293995	12.35434387425469	14.46741946912123
H	10.15368890621273	11.56608787438871	15.24060171033761
H	7.64735100751084	8.05539627127840	16.35162380650285
C	7.55887189412024	5.87903595456484	16.40232903107398
C	9.34418725274182	4.70737885173759	15.23050249414985
H	10.80997876614481	5.99331631776068	14.23406536621105
C	17.13077155830296	9.61065332941549	13.31453588421368
C	16.00975997839387	6.99802559498197	12.69375394763573
Cl	15.06494341503421	13.52717677809918	14.84275792601820
Cl	11.41054239777017	13.94687808942034	15.96966119268033
N	14.53445503649917	6.82577098232597	16.68735699247248
C	16.83734979116483	8.40901250323752	16.44324553470428
C	14.31488980997722	9.65213108024692	17.37162006216611
C	12.73236270176201	6.33202311099857	11.78470424116646
C	11.12345618208792	6.23689022725900	9.91096281322542
C	8.41633948786952	6.74031369682898	12.00703800076906
C	8.12879917597099	8.88670344217778	10.88439691491582
C	11.92492070797177	9.59775782189374	9.57535005671621
C	10.77704542170318	10.98017783960543	11.23501411946666
H	5.68943061544503	10.48742435879945	13.22223854234294
C	7.03746324739924	12.05931560943919	13.90141643398526
H	8.60205493497566	13.38962074273307	14.67577787806625
H	6.64159808612857	5.85579849456743	17.01039480248728
C	8.16739141020768	4.67688748799042	16.00102521333642
H	9.82436525522772	3.76769787141713	14.91562657451105
C	18.39158187921573	8.99811684421854	13.19293748906678

C	14.51529138517562	8.84817771037202	14.60319587470155
P	10.77325648755945	8.43577845705219	11.73165263085067
C	7.37786094614261	8.99572797266714	14.57816678534818
C	8.82452201774289	10.94859142444056	14.30339196350511
C	9.07398481338113	6.61710120451708	16.39391695574552
C	10.02414765974374	5.84951689366988	14.28570080080733
P	15.47561819757373	8.69654066178643	13.17118748266012
P	14.92147435533853	8.49703701852985	16.21335698558562
N	11.36395741701889	6.92179680770530	11.18410628940496
C	8.96412588429152	8.31432878283271	11.48467242224589
C	11.33577822567798	9.79397465557046	10.64385646152790
H	7.25638070150005	7.91603634108110	14.74919319339893
C	6.25010610535499	9.80790166611240	14.36513803346669
C	7.69460061311491	11.74810836479000	14.08917232731673
H	9.83796197072996	11.39100645793438	14.29343324264938
H	8.86664804825493	7.43775792226236	17.09842732198162
C	8.73328877063799	5.29530092912158	16.71784616556810
C	9.68133022341940	4.52688050083967	14.61160337423335
H	10.55391349278535	6.08095451590299	13.34995895378684
C	17.09826062468941	9.53135295081483	13.37023688804685
C	15.90619669960435	6.96823208302254	12.72348992729898
N	14.33505026777410	6.97407727892060	16.73259875183980
C	16.72898462686062	8.38450599835862	16.49062382855141
C	14.32175000088892	9.85925908520358	17.27729577818757
C	12.68412379301903	6.47327570267925	11.65477404030319
C	10.92998509370015	6.28379164501284	9.93851434645525
C	8.27582944418639	7.10413329704039	11.70814839047758
C	8.23553082898447	9.48618116559605	11.19154148748355
C	11.73444354400337	9.53051371036946	9.31626031465284
C	11.37561927266762	11.11596847702720	11.13844789790188
H	5.24506472853855	9.35896951761189	14.38103418838614
C	6.40580592677039	11.18074772612487	14.12199609535080
H	7.81989313246257	12.82561611266767	13.90116186067237
H	8.23649956467876	5.07721703120272	17.67582406396226
C	9.03252993540177	4.24805208654637	15.82562201714676
H	9.93336633919927	3.71077568114652	13.91623040006020
C	18.32985498171315	8.85386487235031	13.36246595633591
C	17.05968809235261	10.92791580776032	13.57551021382535
C	16.38049793561600	6.67202126664424	11.42910193486133
C	15.75464443438094	5.93741626436902	13.66578653620352

C	17.03140979539260	10.99307048523601	13.57617724503714
C	16.47211732004267	6.74954783225696	11.38435773506782
C	15.86388897339397	5.93365796665781	13.59906790575383
C	13.24576442111293	6.31301415641193	16.18761996849178
C	14.99174999689891	6.18037421428051	17.92461156928283
C	17.60540622185311	7.24311122048533	16.24695259968636
C	17.48170805883796	9.64760139594905	16.64937984865118
C	13.74723084273906	9.28346852614063	18.60999159658169
C	14.27631590350504	10.99943064237813	16.95469201432356
H	12.89826423812118	6.85658510388855	12.74557410833769
H	13.60670172298143	6.57090436978544	11.13769730670234
C	12.62275980431827	4.81921792029194	11.99383679400865
C	10.96985892460480	4.71953600732016	10.08276401127904
H	11.89841786669064	6.44141132329245	9.13104664726275
H	10.17785881292556	6.69077292626920	9.55107765247373
H	9.07142432893473	5.99349742659068	12.47440865871208
C	7.03695452720223	6.50826808082583	11.91933876805259
C	6.75052826119658	8.64123107947432	10.78226388354084
H	8.55390358781248	9.82118254771843	10.49147646664047
H	12.26766743851329	8.61125150643893	9.23405669650193
C	12.27892559387849	10.75294900567424	8.86318333924474
C	11.12570095541585	12.12890287032753	10.51040701004703
H	10.24250480075729	11.07433420572403	12.19149600520194
H	6.33964255372213	12.87337858973526	13.65033893502135
H	7.72429401789012	3.71226459549935	16.29255162124543
H	18.46865067806951	7.91687619399228	13.00730768187169
C	19.55391048884759	9.77633242577139	13.32846114468783
C	18.19461595957882	11.76010694711499	13.71334173746583
H	16.05182763243505	11.48742410504394	13.68425521998285
H	16.55274382294474	7.58336740068497	10.66968102098919
C	16.79960803474507	5.44248000808092	10.99516971546650
C	16.19695213394332	4.62619047590975	13.20742608001086
H	15.46766791118497	6.13303228985720	14.60532927988952
H	13.00887528337925	6.83873706628835	15.24134783458851
H	12.42536162875598	6.57350550184384	16.89606980347192
C	13.31119292402547	4.79673029855121	15.97378498608113
C	15.14228431355906	4.67005224865718	17.70998329282040
H	14.25481262015743	6.34417671707404	18.74901946216855
H	15.94516027948424	6.64174510575228	18.24815823525620
H	17.10889625798483	6.28071506690099	16.06522310433515

C	13.07067668908086	6.45619752859858	16.17965840659142
C	14.70603983092733	6.37757811163355	18.02025041641698
C	17.42170610313657	7.16500265185575	16.34689715472799
C	17.45393330040520	9.56986595884672	16.73575729585746
C	13.80000698832036	9.61541366829391	18.56456246568428
C	14.33167752909447	11.17521372912997	16.76594102037886
H	12.89786369821503	6.99187770941306	12.61029535484984
H	13.47510039664362	6.79406076127833	10.93810089484572
C	12.71323157367717	4.95267258778412	11.83496004975194
C	10.86844702705670	4.76149808799486	10.11726529858339
H	11.64961273812066	6.50797425551846	9.11151808893530
H	9.94691796797385	6.69532821110880	9.63623416893290
H	8.83755485423981	6.19226333223328	11.94908168381012
C	6.87606184299607	7.06368136042413	11.62170200228143
C	6.83705638870122	9.44127340413450	11.11438907243733
H	8.76731350521675	10.43465914615011	11.02918057573906
H	11.73122531939315	8.50287868933193	8.93063314235782
C	12.16837438998309	10.57718089141555	8.49109438268021
C	11.82597774615797	12.15530775645854	10.30920078700700
H	11.14667505251894	11.34946719921972	12.19124133997902
H	5.52080719841285	11.81422090152014	13.95485152416804
H	8.76695308145399	3.21170834656718	16.08531655998510
H	18.35896985853939	7.76452010003426	13.21451923186291
C	19.52204876928041	9.57200661524480	13.55964565545507
C	18.25142293811877	11.63719466004452	13.77106005437773
H	16.08860426665005	11.45010752665628	13.55881076533126
H	16.46028059134534	7.48086934373445	10.68583698443598
C	16.72503772531324	5.35347689136813	11.09570042881885
C	16.10681607501277	4.61889970262479	13.33259585782512
H	15.33686207852947	6.17285249340450	14.65519947450354
H	12.89508563711971	6.95129863273584	15.20345633741724
H	12.22053208946041	6.75330647330095	16.83492046364625
C	13.12264797272174	4.93279263662207	16.03203431833320
C	14.83305639841203	4.85452386242896	17.88762780743106
H	13.92605208543152	6.59790037741474	18.79081056366990
H	15.65269380668706	6.82933124475264	18.37777902431629
H	16.86491844264880	6.23947206645577	16.15080326181658
C	18.81969836049065	7.12971622270336	16.46193516087735
C	18.85040181286249	9.53036764268494	16.84229414633152
H	16.92139810901877	10.52554945781846	16.84313090888025

C	19.00641874818485	7.31272281490137	16.27997073745102	H	13.75727711945419	8.59302390886361	18.96104947586949
C	18.88106626959253	9.70997729450594	16.67302515224900	C	13.29109207458869	10.67579351789631	19.32726324176491
H	16.88912451067544	10.56452176221269	16.78170342863453	C	13.80686002095388	12.22934828255241	17.52793828665656
H	13.74493923153618	8.23485292057639	18.93371438435594	H	14.69697910307130	11.37545676580473	15.74849626633732
C	13.13568673238821	10.25857736567641	19.41115655433731	H	12.06987790106754	4.67183183648878	12.69655152172373
C	13.65836486291542	11.96821890757686	17.75777546227231	H	13.74573469739356	4.64854832421402	12.09749184634786
H	14.69736669629903	11.31853933191567	15.98847006733528	C	12.23302409623924	4.22364949047078	10.57268321885061
H	11.85008983769135	4.61530898738720	12.76845700125741	H	10.09161732354125	4.51895597171604	10.87598656270091
H	13.58786863427384	4.43964354097806	12.38671851863810	H	10.55147603191187	4.28872428217958	9.16362222014130
C	12.24304191301514	4.10800254238939	10.68718869775619	H	6.34774222227530	6.11356724961348	11.79401195446457
H	10.10477285611275	4.52689958700322	10.75389830656800	C	6.15484856197333	8.23063123397970	11.32243350351435
H	10.73257794345517	4.25708096466464	9.10146421093565	H	6.27613696576205	10.36213693634020	10.89746316279649
H	6.61413519397864	5.57923745108069	12.33035309198458	H	12.48907541177139	10.36230501188365	7.46024924183055
C	6.20106493304459	7.46002456286743	11.30706669355350	C	12.21495240481985	11.89228819025204	8.98719198190656
H	6.10116973256207	9.38661854625494	10.29921453073821	H	11.89635158537205	13.17059436783631	10.72641435883106
H	12.88896324469231	10.66767586323657	7.95137459740253	H	20.48476720029884	9.03831068932980	13.55978994437583
C	11.87632931679794	12.01710661582655	9.32888239857357	C	19.48592959083362	10.95993505381918	13.76153841494892
H	10.83838394816089	13.11891603283959	10.89472052627569	H	18.21936237932526	12.72678398768805	13.92495556462343
H	20.54120981419663	9.29771878766865	13.23917849084480	H	17.09268816332254	5.12278664482705	10.08398188284746
C	19.45778885809141	11.15395368267984	13.58433188526935	C	16.59664449008760	4.32580464226009	12.04920682731939
H	18.09487986903020	12.83525752069396	13.92610231155818	H	15.98323879135560	3.81572948436634	14.07598875739131
H	17.15655124158861	5.24933228369934	9.97194760210584	H	13.84426197239157	4.66170157123198	15.23238233487369
C	16.66605821435251	4.37862835461582	11.90716865378634	H	12.12665616339309	4.58026863035663	15.69705169346448
H	16.07903259469284	3.79589064586858	13.92135487413655	C	13.52810035926294	4.25307863274094	17.34697657678963
H	13.97990179791695	4.57516209178283	15.11585535269455	H	15.67150112964856	4.62159440678987	17.19439842375684
H	12.30079066617269	4.43553580177477	15.69309652006384	H	15.09628931561986	4.41880153339201	18.87458837084376
C	13.81550669125767	4.06719469927066	17.22662490009031	H	19.35057120018244	6.17164131684096	16.35329458778063
H	15.94061282814940	4.48621256236463	16.95685759577173	C	19.53606710830232	8.31095301719657	16.71065616262264
H	15.47373888962862	4.19393418085342	18.65664229691538	H	19.40713774746077	10.46152005795019	17.02277436077632
H	19.60046162703804	6.39840422253313	16.13034158534796	H	12.86969174036999	10.47629130439120	20.32423254987926
C	19.64488765785588	8.54436194472847	16.49376078079802	C	13.29009102576299	11.98284703897564	18.80873355092635
H	19.37669502687002	10.68037689359632	16.82007483761510	H	13.77552246522583	13.23827385863790	17.09208321770603
H	12.67311928557435	9.96379266462641	20.36543353762571	H	12.97339398397993	4.38345423376959	9.75576962191882
C	13.08333286473597	11.59645922102864	18.98273947448471	H	12.18779122355018	3.12717250371577	10.74302085792109
H	13.59562806957894	13.00060989785135	17.38628510999360	H	5.05632635642415	8.19976974485623	11.26069950068710
H	13.08024877956393	4.21713994228877	9.96053406385225	H	12.57416554646878	12.71005467398589	8.34341403627335
H	12.11252397527523	3.01781178730210	10.85262657704218	H	20.42216477980567	11.51891305968965	13.91359312584114
H	5.11799237505559	7.27692263129134	11.23623248931530	H	16.86705988754705	3.29223041325035	11.78375260171015
H	12.16812707018729	12.92348256705737	8.77670754226535	H	12.72107075636720	4.40378759212158	18.09977718409801
H	20.37271482605851	11.75765351157160	13.68911700780733	H	13.62761835052414	3.15541587875997	17.21098695279547

H	16.92126721983181	3.35356528161608	11.59765913908819
H	13.05834389656524	4.16739525427270	18.03720955779552
H	13.92412807174719	2.97952039238338	17.03199566254208
H	20.74388349530568	8.59967134393299	16.51008514396190
H	12.57145732578173	12.35254177793572	19.59710024080337
H	20.63312145326478	8.28385819191612	16.79503008354811
H	12.86542973365752	12.80823458219841	19.40039846098818

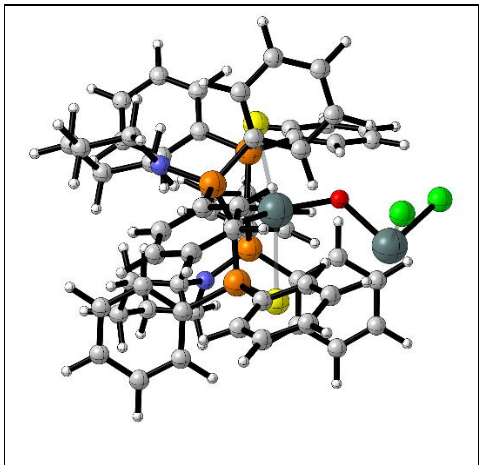

5[134]

E = -6019.415622

S	11.307513	9.230809	16.332115
P	10.067799	8.634594	14.809327
Sn	12.788536	9.674222	14.115516
C	11.134154	8.491609	13.445346
C	8.773733	9.887788	14.516233
C	9.285851	7.053754	15.273997
S	14.306468	9.535730	11.816526
O	12.469799	11.603211	14.158542
C	14.590492	8.689758	14.670760
P	10.735254	8.281155	11.800184
C	7.439040	9.574959	14.201151
C	9.230802	11.224844	14.445280
C	8.190420	6.989850	16.156430
C	9.886293	5.867373	14.811758
P	15.540698	8.693649	13.223358
Sn	13.380587	13.044352	12.927038
P	15.039786	8.358803	16.280236
N	11.459989	6.878315	11.180896
C	8.946611	8.017933	11.550910

C	11.185457	9.741878	10.813156
C	6.556471	10.603710	13.830941
C	8.349427	12.239275	14.050506
C	7.679393	5.740505	16.541701
C	9.378319	4.621387	15.204077
C	17.079038	9.656126	13.405063
C	16.039471	7.026973	12.661228
N	14.528802	6.805815	16.780415
C	16.851083	8.327968	16.467292
C	14.357272	9.668050	17.341492
C	12.678908	6.318946	11.774657
C	11.076559	6.305810	9.889242
C	8.385414	6.805592	12.003138
C	8.115047	9.016347	11.010535
C	11.915879	9.632873	9.614537
C	10.797730	11.009460	11.293569
C	7.009309	11.930318	13.750942
C	8.268002	4.556630	16.064208
C	18.366762	9.109179	13.269462
C	16.917579	11.015106	13.740191
C	16.586461	6.842612	11.375459
C	15.807768	5.918422	13.492006
C	13.251844	6.268544	16.279710
C	14.990406	6.198022	18.033740
C	17.578455	7.156261	16.178185
C	17.533304	9.525988	16.758898
C	13.729096	9.369856	18.568094
C	14.383116	10.996905	16.869634
C	12.548706	4.801183	11.929685
C	10.906991	4.785240	10.005622
C	7.000905	6.603191	11.934402
C	6.730940	8.802868	10.930377
C	12.244151	10.790975	8.893728
C	11.118935	12.163826	10.563789
C	19.489576	9.931834	13.461466
C	18.038035	11.829516	13.933302
C	16.912322	5.551829	10.935184
C	16.139536	4.627566	13.050560
C	13.336824	4.749686	16.100939
C	15.146431	4.682386	17.862069

C	18.980166	7.179909	16.197145
C	18.934111	9.544122	16.767813
C	13.129410	10.397736	19.308792
C	13.776602	12.019488	17.610994
C	12.170521	4.140807	10.596272
C	6.171370	7.602552	11.396368
C	11.840321	12.051601	9.363058
C	19.328082	11.287889	13.788554
C	16.691997	4.442693	11.772937
C	13.828469	4.058316	17.380194
C	19.658460	8.372955	16.491994
C	13.149370	11.719544	18.829363
Cl	11.310693	14.465611	13.293147
Cl	14.975853	13.778328	14.808775
H	7.091593	8.531589	14.210386
H	10.286476	11.480387	14.654667
H	7.741195	7.915767	16.545464
H	10.751320	5.948610	14.135209
H	5.512299	10.358126	13.583984
H	8.740971	13.264937	13.962300
H	6.817413	5.690613	17.224340
H	9.846734	3.696365	14.833463
H	12.839662	6.807470	12.755473
H	13.562340	6.571675	11.144154
H	11.856675	6.527919	9.119650
H	10.137630	6.784243	9.544263
H	9.038491	6.022682	12.410376
H	8.547132	9.965012	10.662818
H	12.256579	8.649305	9.264434
H	10.275037	11.105973	12.255461
H	6.315937	12.729363	13.445684
H	7.862883	3.579669	16.369169
H	18.494991	8.043954	13.028764
H	15.919069	11.463105	13.858885
H	16.736085	7.711520	10.716186
H	15.348166	6.075267	14.478676
H	13.021748	6.768878	15.318386
H	12.420213	6.534411	16.972631
H	14.257938	6.388463	18.856553
H	15.946425	6.669141	18.336565

H	17.044645	6.226604	15.939777
H	16.969067	10.444918	16.971939
H	13.672806	8.333665	18.926531
H	14.838094	11.263521	15.904999
H	11.766913	4.581484	12.690446
H	13.505675	4.394967	12.315539
H	10.033550	4.573893	10.660283
H	10.674851	4.361391	9.005894
H	6.569732	5.659671	12.301417
H	6.085440	9.588522	10.510832
H	12.833788	10.708049	7.968346
H	10.837405	13.146791	10.973321
H	20.498775	9.504309	13.360227
H	17.874240	12.885330	14.197154
H	17.335927	5.406897	9.929656
H	15.956951	3.761855	13.706076
H	14.025480	4.516098	15.260522
H	12.335565	4.371661	15.808324
H	15.952983	4.483950	17.122090
H	15.470776	4.233674	18.824357
H	19.543790	6.261419	15.974221
H	19.461848	10.484304	16.983213
H	12.620303	10.159815	20.254952
H	13.793023	13.035573	17.188932
H	13.013790	4.269074	9.879988
H	12.029260	3.046714	10.721646
H	5.083729	7.443404	11.339580
H	12.112205	12.959241	8.803242
H	20.213503	11.925493	13.935953
H	16.946289	3.430655	11.422700
H	13.059756	4.178297	18.176948
H	13.945979	2.966186	17.219092
H	20.758730	8.393439	16.498590
H	12.654894	12.517589	19.403994

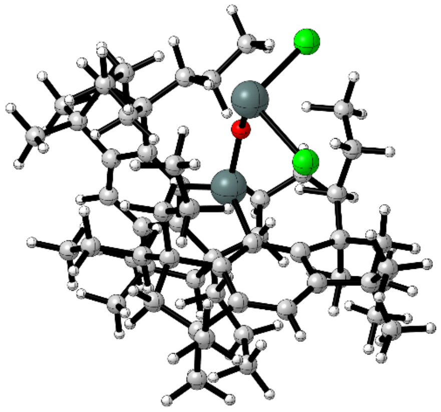	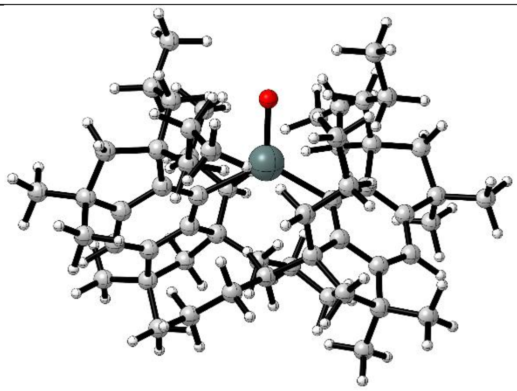
**I-SnCl$_2$**	**I**
E = -3612.724694	E = -2477.58966
Sn 9.34942958320023 3.55864513217565 5.35422705409398	Sn 9.47926562287716 3.66345182890883 5.57406573676814
C 7.97040049310737 2.64817756957346 6.81281543590226	O 11.16976097932771 2.82372610517075 5.72258811744893
C 9.21810077624783 5.39871771571846 4.20438603547142	C 7.77351200875909 2.77716375104507 6.73365267901227
C 7.24311489744400 3.46614203475714 7.71498975069343	C 9.32703061639439 5.50037902784219 4.30058614769692
C 7.65447867615630 1.26541196686133 6.73205077459300	C 6.99228725635502 3.61605436632037 7.56898940010726
C 10.25611988219353 6.34600914815207 4.29050350142665	C 7.45646580772179 1.39150783981949 6.69477771941566
C 8.19845563727462 5.57363246631590 3.23636419387065	C 10.25349336299808 6.57601689258105 4.39175026896592
C 7.51790807105580 4.91103316179857 8.16533309476547	C 8.28166990316502 5.59802922016822 3.34683345833335
C 6.14085647629667 2.93834671818614 8.42065576639955	C 7.29252222066752 5.05212856370765 8.02985253826122
C 6.53742814426956 0.76993844091441 7.45029003785954	C 5.84794915800030 3.12220839265143 8.22830352360464
C 8.33365171495252 0.12332084657999 5.95627074173286	C 6.29862692452270 0.92300465687262 7.36639754518705
C 11.34822465193816 6.58478992349304 5.35414544088244	C 8.20917298210931 0.20281404231186 6.06127443638107
C 10.31328068236892 7.40612473314047 3.35305332036489	C 11.59729091817140 6.69146183256384 5.14368396237875
C 8.17921763794745 6.74103382016892 2.44618110753771	C 10.01821965575246 7.75922743170670 3.64564610124373
C 7.10207162576842 4.60224711725076 2.78103842825719	C 8.07464367614665 6.78545244033100 2.61549855367617
C 6.56618163027361 5.07399645144604 9.39225119585894	C 7.34814151874510 4.48800857975955 2.83413808447033
C 7.16463760450999 5.90607741645810 7.02798261594954	C 6.25853374279543 5.27431064817327 9.17931870585649
C 9.00328325832717 5.04157001843096 8.57842381135096	C 7.07006904922555 6.06456501520216 6.87880026108052
C 5.46441468058942 3.98171535597503 9.30074736092509	C 8.74364873095305 5.10484790052155 8.56174245556650
C 5.76306803256694 1.60045906289425 8.26733766021919	C 5.13968926872764 4.20081508569850 9.03896229924067
C 6.28637599875556 -0.71104409410499 7.19225986075745	C 5.47304723992428 1.78102606801341 8.10104373329897
C 7.62833321538028 -1.13981248522227 6.54638116569194	C 6.07601929591914 -0.57268905853735 7.17895848967919
C 8.02106773983646 0.29061553215586 4.44297234762363	C 7.46430996537679 -1.03195916916877 6.66142291042999
C 9.86325598859485 0.00583333039581 6.20196703854316	C 8.04258289362590 0.24256760387457 4.52239904781943
C 12.40217708197729 7.35298555762380 4.51158767800353	C 9.71093725057441 0.15530961883737 6.44248315345051
C 11.90499248165862 5.34889637020105 6.08191148564522	C 12.21005933256273 7.99741584771662 4.54571401567683

C	10.67473147386746	7.49685834692304	6.43113126388248
C	11.61837544536147	8.19199601655636	3.45526129886578
C	9.25745864551061	7.63779636964657	2.46660383716704
C	6.88763819590382	6.87866328562666	1.64835068989515
C	6.19525508610192	5.48972602263807	1.86191464079448
C	7.75151445739116	3.44551371829435	1.96599444035926
C	6.29828310926681	4.04106285752802	3.96983225601918
H	7.13972343484093	4.91938897010385	10.32709928639089
H	6.13619443249110	6.09234687166748	9.45323464372643
H	7.85286951272946	5.73588553349426	6.17373734078202
H	6.15705617838293	5.65075890084797	6.63641471804991
C	7.20570263078643	7.40395791627450	7.35068412441561
H	9.61918399406069	4.93016179282122	7.65842040293807
H	9.20963935303585	6.07658453750490	8.92516986548168
C	9.51971379188983	4.03304636014687	9.61213952090377
C	4.19373444819063	4.50849388189403	8.59347234549133
C	5.09263772353587	3.43334126516919	10.68886620463999
H	4.89077224095390	1.19806824868687	8.80549846988405
C	5.09118218308868	-0.87771584141340	6.22502398125362
C	6.00525815205508	-1.49065554850026	8.48938330034883
H	7.49735929466492	-1.93394866149145	5.78404924687998
H	8.28009301821214	-1.56362534969789	7.33723725075103
H	6.91931385536681	0.26493242176543	4.30267397713118
H	8.33483063859816	1.30721913467484	4.12044548847521
C	8.68259478591309	-0.71690560237195	3.50002154005778
H	10.15315677024512	-1.03867852743596	5.95304348347650
H	10.41000261256403	0.65895225525399	5.49516762131518
C	10.34928699178925	0.33158299991897	7.61787916553957
H	13.08968000563994	7.96626717016414	5.12701225481890
H	13.01312178092683	6.60706718895583	3.96861555366718
H	12.18104453939545	5.63201526306055	7.12016823077478
H	11.03800686614505	4.64661722935821	6.29792571461362
C	13.02839141756470	4.51711529691981	5.48279051402244
H	10.03781615628720	8.25021359500441	5.92637178908700
H	9.96748648026337	6.85840060792637	6.99368678064528
C	11.60093637289225	8.21056591661286	7.41985416327886
C	12.35265628625984	8.17091552892921	2.09800098368724
C	11.38630648651039	9.65663875793181	3.88623103179495
H	9.28046898520579	8.50215088775423	1.78372240931277
C	7.12198323066439	7.22455793778333	0.16651284384936

C	12.54171072058102	5.49004722010875	4.88669861341180
C	11.34249387803603	6.85457367239349	6.66304771164021
C	11.07452292533496	8.82881286196613	3.89483953868332
C	8.91909577986767	7.88691107280600	2.78931200646197
C	6.87910851068507	6.68363028428709	1.67621280515055
C	6.68551023333674	5.14234950318609	1.57950132163977
C	8.20287060145748	3.26135750502402	2.44223926774894
C	6.28547017805671	4.12116121071377	3.89964059285499
H	6.75703381933314	5.14153551286140	10.15995797315259
H	5.84845211906230	6.30410362926940	9.17054523347250
H	7.62303270745114	5.73103948639000	5.97816473132579
H	5.99952043155941	6.01756170575610	6.58527609620961
C	7.45742872752452	7.52097009914574	7.15065247241940
H	9.42920205697770	4.98005736627684	7.68686094756812
H	8.98073556452738	6.12337344459324	8.93539843812895
C	9.13639821218092	4.04790902733526	9.60154322873781
C	3.93073068430791	4.73083022383153	8.23353745944956
C	4.66501802366569	3.69812174141213	10.41247802198093
H	4.57312751432507	1.39639543888113	8.60564294110957
C	4.95808850769399	-0.80337596684490	6.13543553327916
C	5.70132928516439	-1.27886718451984	8.49403488672617
H	7.38284276665882	-1.85919636477799	5.92737843052316
H	8.05181820270506	-1.42453587007529	7.51543819284551
H	6.95811226266199	0.17881720968326	4.28588385785610
H	8.35748327957512	1.24126010040091	4.16022242415375
C	8.79932852133695	-0.82275949796679	3.72000024759791
H	10.09610529851503	-0.84886327523372	6.15851356329762
H	10.28717682139317	0.89310597899605	5.84406445080905
C	10.03604873636323	0.42105717745700	7.91632872445323
H	12.76833106776840	8.57704838460602	5.30834000232123
H	12.94207825808786	7.72244465626807	3.76008735380809
H	13.55103707527406	5.77125204944731	5.26052381505190
H	12.21604465134841	4.61275447865068	5.48630627827997
C	12.65898467271069	5.01996559417969	3.43304046883150
H	10.72004369813475	7.76201663041493	6.81940407894516
H	10.71672376266281	6.00742465311040	7.00704610686622
C	12.57955045891276	6.92556196101233	7.56737857122563
C	11.54603258946718	9.50349039423349	2.59398588810919
C	10.51099688550378	9.90308985592180	4.85412326415717
H	8.75157572726845	8.81998463935007	2.22892744658425

C	6.03578945968510	7.99674142689941	2.29549365462623
H	5.99650762985028	4.98536271295605	0.89588864706441
H	5.20556089983725	5.63670829571275	2.33966908919915
H	8.40058749431566	2.84707817462251	2.64842555444431
H	6.94276349778941	2.75341311433827	1.64602541761754
C	8.57494329885590	3.86938059628562	0.74683585702571
H	6.98274592180829	3.53164024424801	4.68882768312216
H	5.88070549433819	4.89654212444377	4.54268354363942
C	5.18589683480257	3.03424975397080	3.66372564468991
H	6.43207953830850	7.65695511720278	8.10747282187459
H	8.17775414356682	7.66930891579685	7.81468655448510
C	6.99819913845447	8.25250647290849	6.09207013799042
H	9.08016045570285	4.25478856103868	10.60834357967730
H	9.16177336584248	3.01578238005351	9.34471623438471
C	11.04766595337571	4.02693484051821	9.71211942716626
H	4.43309084764974	4.94129959444273	7.60243190464786
H	3.70303411954772	5.29445865681904	9.20450045951801
H	3.46232621497767	3.69035920877216	8.43107423322117
H	4.30248708548727	2.65707116792439	10.62388773961973
H	4.70462957771427	4.24626107356645	11.33713982044549
H	5.97443808875021	2.98325114610234	11.18832037728287
H	4.15756635522011	-0.48373934349564	6.67693976758433
H	4.93178910158489	-1.94953729168888	5.98512578608193
H	5.25756720365803	-0.33310849244301	5.27587564214049
H	6.81726161726395	-1.34033884235446	9.22957186496176
H	5.92644353877213	-2.57750094618610	8.27941934427939
H	5.05063279936609	-1.17312981074175	8.95756133172993
H	9.78590537644072	-0.59733691683245	3.54877723713094
H	8.47344080322065	-1.75300799902026	3.84636599406600
C	8.21654049462378	-0.54914339888481	2.05211960901093
H	9.72416489363613	-0.18061675269216	8.38478147547740
H	10.21126829367683	1.41826481731832	7.81220256072997
C	11.82461120092774	-0.03558020161114	7.80419994407698
H	12.76812231850830	4.19095624317471	4.45657386014889
H	13.92581904819617	5.16441384266393	5.37446737219169
C	13.35676348425603	3.31439009257906	6.36615847784691
H	12.29647674912775	7.48326073831363	7.89308719326816
H	12.24650040002648	8.93202338891375	6.87482502399397
C	10.82067457734995	8.94989685954224	8.51211664529787
H	12.47122549097497	7.12992188937863	1.73696350743218
C	7.15424583697521	7.30706113063820	0.29739226166346
C	5.65720982571142	7.37673580718849	2.32407566403458
H	7.18692636560453	4.77888804610963	0.66091300631470
H	5.61684333968146	4.86326724464804	1.48712360878869
H	8.61864637801759	2.82129724954405	3.38262120605246
H	7.55499936929697	2.45301845264464	2.04179625852158
C	9.38691575718361	3.51284836474665	1.50093694279741
H	6.78692518020847	3.87483803275011	4.85703151840154
H	5.69171779832797	5.03382396636902	4.11936384169335
C	5.33577117076659	2.96424437117544	3.57391214371110
H	6.98306124045032	7.87253009441744	8.09379352704132
H	8.55348033326307	7.58922562870441	7.31512875148977
C	7.06713413087432	8.44207262174970	5.99183759545981
H	8.69779372901283	4.30971004014445	10.58889440114659
H	8.68729714115222	3.07201773004179	9.32076798949305
C	10.65428410349667	3.88383521995312	9.72275752525706
H	4.24146475142498	5.10820129539950	7.23939258435489
H	3.42486361992528	5.55676654118723	8.77641336980155
H	3.18776650651069	3.92480096673138	8.06212595712438
H	3.86911443863913	2.93088106624364	10.31558615195032
H	4.24678493017518	4.53427437125076	11.01075532626096
H	5.50365957732303	3.25010902585942	10.98321570339519
H	3.99546326264537	-0.37763552833938	6.48645047223317
H	4.80989299874515	-1.88836874819881	5.95295391590229
H	5.20036686574864	-0.32119800173012	5.16847756328966
H	6.45382251212818	-1.07476409691645	9.28262286437405
H	5.64754576424077	-2.37730441860815	8.34380786540927
H	4.71157546562868	-0.94732685384363	8.87138249404105
H	9.88411059738165	-0.58691373939581	3.72050586040908
H	8.70298210454254	-1.81136888442310	4.22053969338225
C	8.28921803748310	-0.93533020000093	2.27999569833347
H	9.53274641009347	-0.32366317223818	8.57423376602242
H	9.61483036482212	1.40696695193635	8.20504872113182
C	11.54556840352457	0.43169697524403	8.17009439648813
H	11.64630294466900	4.77800720952898	3.04688223635361
H	13.04004588069061	5.83633904999665	2.77816783605416
C	13.54583572223255	3.77723667388818	3.31645629346617
H	13.03259149987818	5.91595302392005	7.65538254929132
H	13.35783980243474	7.56776372451051	7.09961273047615
C	12.24740750968354	7.47319737226479	8.95903935920567

H	13.35936794114355	8.62901970016640	2.19616137056241
H	11.79436336826367	8.74579114098161	1.33005286302289
H	10.77851647594492	10.19480403758605	3.12979721929712
H	12.35386094845495	10.19166787565485	3.98096677606303
H	10.85580449385041	9.73341865611626	4.85438909218112
H	7.60947566106856	8.21544294541830	0.06131848050103
H	6.15718245404519	7.27031360061031	-0.37980367622785
H	7.76927576684206	6.47860516747371	-0.33407601971216
H	5.84978831250244	7.77692635403404	3.36536350869056
H	5.05595266238993	8.08940195265777	1.78125768183754
H	6.54996613355834	8.97824413590391	2.23784492235004
H	7.98583365436138	4.59011740091366	0.14369508506542
H	9.48394317773346	4.41115370623827	1.08258631194429
C	8.97390237244031	2.70004858073879	-0.15568934352762
H	4.51333448517488	3.44549391241511	2.87908006917916
H	5.62620187283203	2.10942636796317	3.23495255490430
C	4.37866794784830	2.68200580385076	4.91587469590696
H	7.74934863551345	8.00713807301319	5.31180755950330
H	7.07428545390259	9.33693721280958	6.30956262241170
H	5.99672952236032	8.07136900079164	5.64837069382547
H	11.51242153096355	3.70097461686076	8.75894539232196
H	11.40095477859349	3.33473838224794	10.50234046793347
H	11.44672313253665	5.03642851752763	9.94675180463037
H	7.11832899361521	-0.68706908832097	1.96030982160767
H	8.70699809194893	-1.27831083576813	1.37630660674799
H	8.45639535695607	0.46433185387751	1.67212608362599
H	11.97505810189230	-1.13360917362502	7.73760449500804
H	12.21567815724660	0.29971501415954	8.78637686579572
H	12.44865984614147	0.42600801100847	7.01119106036400
H	13.67462445455170	3.62689913262487	7.38417770054039
H	14.15155227051837	2.69360039325621	5.91261523683957
H	12.46856785372734	2.65684021589174	6.46224983207149
H	10.12190417209798	9.69422489970168	8.07580744761998
H	11.49584056090968	9.49083983691312	9.20566553549118
H	10.21296077043952	8.24704364406360	9.12045469594873
H	9.60804573327042	1.96577678814967	0.38424649350748
H	9.56072012944053	3.05155012460626	-1.02792228190776
H	8.08583109230738	2.15257750038436	-0.53727540825748
H	5.03212610714881	2.27209177454098	5.71240748485123
H	3.59633823420579	1.92654009348350	4.70131817732744

H	11.88080549783848	8.74779418913934	1.85474720174697
H	12.39591263324234	10.18754732024430	2.79824008893795
H	10.73985882143275	10.10676695620267	2.12724230890618
H	9.63818754446735	10.41648650339253	4.40068759864314
H	11.28293277914347	10.66793415838454	5.08099660759551
H	10.17388099131723	9.45842795700459	5.81064821692156
H	7.28981465318275	8.40612212538412	0.36689539831595
H	6.30468404034443	7.12066826650299	-0.39205279673253
H	8.07034344255484	6.87816097989657	-0.15730196047645
H	5.42745248663576	6.94452456964786	3.31769397608333
H	4.75798447753561	7.26959626252671	1.68188020969409
H	5.85099782262753	8.45917926685083	2.47067958862796
H	9.02063906081414	3.65329206167542	0.46099176013439
H	9.88140378486308	4.46706900129769	1.77976485860461
C	10.41720485036524	2.38027708035504	1.54939245277438
H	4.84495514957066	3.13384659106203	2.58974873529235
H	5.91605534364127	2.02369810480462	3.46607673212799
C	4.27377893703612	2.77716218729408	4.66130559623189
H	7.56029980564043	8.12444958724159	5.05120403080229
H	7.36132374706279	9.49336073334154	6.18548949436101
H	5.97087232460509	8.42573167050108	5.81566308495434
H	11.09567525526967	3.56911686808095	8.75353858795213
H	10.92088294257889	3.11502539022287	10.47506505416510
H	11.14723433905342	4.83152793404013	10.02256432920506
H	7.22771391259751	-1.26047244207463	2.25456597476012
H	8.87949127045568	-1.66375743656894	1.68778180242793
H	8.34351336038774	0.04165739154983	1.75621257325025
H	12.00869377083183	-0.54330988945768	7.90706247875905
H	11.78323836476971	0.64084475000400	9.23328574993323
H	12.01727315447292	1.21519036579587	7.54211579611657
H	14.57999195796985	3.98200882657840	3.66685359194453
H	13.61135454958348	3.41225597877760	2.27095432330327
H	13.12851306948552	2.96578185989124	3.94775876337479
H	11.87689642109629	8.51842372205959	8.90031321130999
H	13.13125145694483	7.46551618023685	9.62880540024912
H	11.45289392272683	6.87298287560626	9.44919975547114
H	10.83508554295582	2.27086760718624	2.57256330847955
H	11.26621536998891	2.57277157754234	0.86362060968702
H	9.97008772880380	1.40532059077265	1.26471404497369
H	4.74410782082722	2.58463402848185	5.64648112692324

H	3.87443022403400	3.57783237039958	5.33464611603480
O	10.62771276208211	2.37508758837622	4.51708157822018
Sn	11.47905318553109	1.96800965450236	2.59570439530764
Cl	11.95626725910557	4.38045572140414	2.00374751132888
Cl	13.66596049575946	1.32637832556844	3.55195866117620

H	3.60222668574390	1.92393319615850	4.43744984571419
H	3.64263464005917	3.68458930462804	4.76713450591354

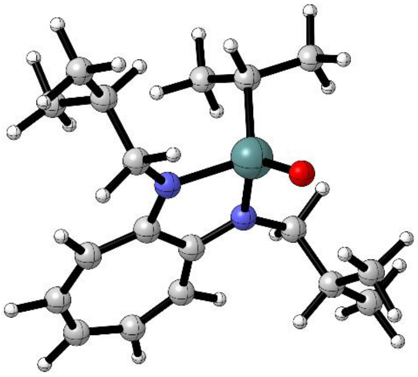

B (no LA)

E = -2965.972858

Ge	-0.00674527828944	-0.38872060376936	0.79788655314288
O	-1.15115968733635	-1.21773888812794	-0.10198193431719
N	0.58638294692028	1.29727819951270	-0.01839858303709
N	1.88539152088523	-0.83941344375900	0.51962233383417
C	2.62688887587648	0.09768597641841	-0.09818656084868
C	3.99829116100247	-0.12407883280679	-0.40375093796769
H	4.36638914723511	-1.11651025771831	-0.11023890741071
C	4.96749091112003	0.68724577086433	-1.00322097344540
H	5.96514982785996	0.22289950865148	-1.07898591754192
C	4.85932332353026	1.98247024525893	-1.52791398046272
H	5.76827228273305	2.42497127590267	-1.96341659050228
C	3.69245073926303	2.76041672943647	-1.55846600391259
H	3.79385578676876	3.75425777984510	-2.02612530508410
C	2.41007997247853	2.47729769671342	-1.07994608906965
H	1.68570809709117	3.28817588541630	-1.23366593152617
C	1.87099813331606	1.34428654852671	-0.41066559841030
C	2.39739059146934	-2.15644657580409	0.87203331874710
H	3.40697638472696	-2.05706026614371	1.32971318080574
H	1.73906720332109	-2.57128987533617	1.66490358577593

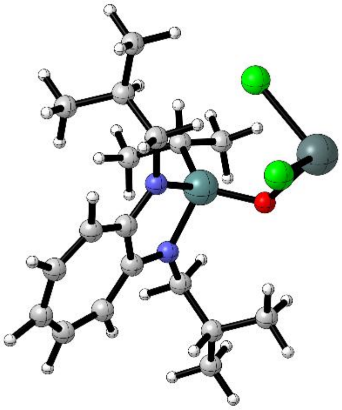

B

E = -4101.121640

Ge	-0.02244257929052	-0.32499888058324	0.79087397815553
O	-0.95273593248774	-1.50293988215066	-0.08537309876221
Sn	-2.93141811985554	-1.13688531726742	-0.78018606985497
Cl	-2.16919071590127	0.42106959950858	-2.56494598787372
Cl	-3.09991720746116	0.65130411070497	1.06012363419165
N	0.46940235594169	1.30387689990193	-0.11533082397441
N	1.81476672824936	-0.80872789933384	0.45780681176728
C	2.53644290782190	0.12360228470267	-0.20003683284788
C	3.91937953164402	-0.06363299101316	-0.45703336833103
H	4.32321740162662	-1.00822392082586	-0.06848205663026
C	4.86601153846383	0.73441706189476	-1.11089745533898
H	5.88183379909262	0.30631012440743	-1.13933854552011
C	4.70960558074301	1.97666505753391	-1.73709094288695
H	5.60056891654930	2.41402530832428	-2.21301861024021
C	3.51927937030103	2.71633148933820	-1.81086172965424
H	3.58631329663781	3.67496472156999	-2.35165387922662
C	2.25231021085548	2.43767342539530	-1.29205964268393
H	1.50668730029563	3.21875138840569	-1.48625075336722
C	1.74860586455087	1.33361504582170	-0.55391568591865

C	-0.43240253661158	2.29652968010314	-0.30390202634296
H	-1.35510662316379	1.73272539832821	-0.56320967107201
H	-0.17058588538846	2.89627840495036	-1.20232028603616
C	2.44191050295439	-3.15739164617063	-0.30949886797822
H	2.90464129437233	-2.62205332993430	-1.16963807071476
C	-0.73498917888945	3.23350533717965	0.88684096148855
H	-0.99824514562148	2.57775748446011	1.74733906872844
C	1.03445844805078	-3.61271054828033	-0.71925302384775
H	0.58920099433247	-4.24331477530626	0.08080959836828
H	1.07620350469432	-4.22617327774609	-1.64248532076864
H	0.33208431660980	-2.76648855654527	-0.87949274400453
C	3.34006226984019	-4.34728985885394	0.05685045638813
H	4.38173139177144	-4.03341718191049	0.28058954518555
H	3.37850546550270	-5.08375878738111	-0.77092075363993
H	2.94814191067932	-4.87651699244589	0.95197609373965
C	-1.95460849937353	4.10504916596240	0.55993005854006
H	-2.21812485259137	4.76098278111172	1.41403178822990
H	-2.84380523130110	3.48820224221533	0.31730005915354
H	-1.74990730090288	4.76049065894817	-0.31368547409723
C	0.47779973974724	4.08002076017835	1.29234059614050
H	1.37033612225004	3.45236036205583	1.48784874565181
H	0.26628241790088	4.66161707350270	2.21262925328226
H	0.74308374672683	4.80606115168748	0.49428341344398
C	-0.16724739278960	-0.09034349398132	2.76100970865386
H	-1.04514663776903	0.58869215017880	2.85017470930159
C	-0.50988024419098	-1.41375421277973	3.46119592174953
H	0.34363843609781	-2.12413524003435	3.42517312915297
H	-1.37809989821198	-1.91323410318595	2.98666289330140
H	-0.74763967994280	-1.24841282352823	4.53474826248420
C	1.06915433960547	0.60167071438769	3.34090010659092
H	0.94068043964129	0.81509619520705	4.42453437344562
H	1.28281376333965	1.56471707316857	2.83414792483188
H	1.97411710587693	-0.03253607156894	3.23448533541728

C	2.36714800862279	-2.08483070623069	0.90077615048827
H	3.31829622815554	-1.90094917144333	1.45028205093914
H	1.66187980373272	-2.51326613211284	1.64381495361132
C	-0.50053420057826	2.38755765428979	-0.28142736372684
H	-1.47864524345051	1.91802357687356	-0.49538849429075
H	-0.25967287726015	2.99271152926252	-1.17805184474067
C	2.59918093485667	-3.12849884071193	-0.21924321089610
H	3.14659026792840	-2.61569382410169	-1.04170659410246
C	-0.62908629404717	3.28429753585672	0.96582543389636
H	-0.88539270126017	2.61009872641888	1.81290527242876
C	1.27988925549429	-3.66269086794813	-0.78798556454185
H	0.73868021900747	-4.25891402991919	-0.02225346279265
H	1.46787094147907	-4.32246344194405	-1.65927548804504
H	0.59781217233263	-2.84566592928737	-1.09305487554721
C	3.48976893055860	-4.25744436530338	0.31745536765062
H	4.47761958691240	-3.88331092632191	0.66003260257329
H	3.67035167045618	-5.02452821453354	-0.46210321538846
H	3.00659405022483	-4.76817696071891	1.17806338400634
C	-1.81240761955582	4.24007105290934	0.76838605187028
H	-1.95306945151232	4.88732096507759	1.65767851972178
H	-2.74839740058339	3.67027314376411	0.60385586596212
H	-1.64897927904555	4.90311727724378	-0.10820951250058
C	0.66870320890082	4.02419813279717	1.31171259601345
H	1.52359938446014	3.32863215870007	1.43984238500386
H	0.55871225396785	4.59449411741739	2.25675698967426
H	0.94181985804734	4.75034995822172	0.51636044643505
C	-0.24455634676964	-0.11474479527830	2.74388777319404
H	-0.98229349877412	0.71073573347969	2.81624158243414
C	-0.87861978458328	-1.37742579292968	3.33935858054537
H	-0.23409500923763	-2.27254939873540	3.21278444245551
H	-1.85592427540203	-1.58751051575716	2.86361644744168
H	-1.05058032285224	-1.24357651623206	4.42879328250384
C	1.07554743767351	0.29891360205758	3.40223813266000
H	0.91708766533591	0.51214407070609	4.48096638174521
H	1.50431229245975	1.21372306003033	2.94386897644715
H	1.84280288652840	-0.50017849193199	3.33645001586771

5. References

[1] Bailey, P. J.; Coxall, R. A.; Dick, C. M.; Fabre, S.; Henderson, L. C.; Herber, C.; Liddle, S. T.; Loroño-González, D.; Parkin, A.; Parsons, S. The first structural characterisation of a group 2 metal alkylperoxide complex: comments on the cleavage of dioxygen by magnesium alkyl complexes. *Chem. Eur. J.* **2003**, *9* (19), 4820–4828.

[2] V. S. V. S. N. Swamy, M. Kumar, F. Krischer, K.-S. Feichtner, B. Mallick, V. H. Gessner, *ZAAC* **2024**, *650*, e202400079.

[3] M. Jörges, A. Kroll, L. Kelling, R. Gauld, B. Mallick, S.M. Huber, V. H. Gessner, *Chem. Open*, **2021**, *10*, 1089-1094.

[4] M. Jörges, S. Mondal, M. Kumar, P. Duari, F. Krischer, J. Löffler, V. H. Gessner, *Organometallics* **2024**, *43*, 585–593.

[5] M. Jörges, F. Krischer, V. H. Gessner, *Science*, **2022**, *378*, 1331-1336.

[6] a) G. M. Sheldrick, *Acta Crystallogr.* **2008**, *A64*, 112. b) G. M. Sheldrick, *Acta Cryst.* **2015**, *C71*, 3. c) A. Thorn, B. Dittrich, G. M. Sheldrick, *Acta Cryst.* **2012**, *A68*, 448. d) G. M Sheldrick, *Acta Cryst.* **2015**, *A71*, 3.

[7] GaussView, Version 6.1, Roy Dennington, Todd A. Keith, and John M. Millam, Semichem Inc., Shawnee Mission, KS, 2016.

[8] Neese, F. Software update: the ORCA program system, version 5.0 WIRES Comput. Molec. Sci., 2022 12(1)e1606 doi.org/10.1002/wcms.1606

[9] a) C. Adamo, V. Barone, *J. Chem. Phys.* **1999**, *110*, 6158 – 6169. b) A. Schaefer, H. Horn, R. Ahlrichs, *J. Chem. Phys.* **1992**, *97*, 2571 – 2577. c) A. Schaefer, C. Huber, R. Ahlrichs, *J. Chem. Phys.* **1994**, *100*, 5829 – 5835.

[10] (a)A. D. Becke, *Phys. Rev. A* **1988**, *38*, 3098−3100; (b)J. P. Perdew, *Phys. Rev. B* **1986**, *33*, 8822-8824; (c)J. P. Perdew, *Phys. Rev. B* **1986**, *34*, 7406-7406.

[11] F. Weigend, R. Ahlrichs, *Phys. Chem. Chem. Phys.* **2005**, *7*, 3297 – 3305.

[12] S. Grimme, J. Antony, S. Ehrlich, H. Krieg, *J. Chem. Phys.* **2010**, *132*, 154104

[13] S. Grimme, S. Ehrlich, L. Goerigk, *J. Comput. Chem.* **2011**, *32*, 1456−1465.

[14] NBO 7.0. E. D. Glendening, J. K. Badenhoop, A. E. Reed, J. E. Carpenter, J. A. Bohmann, C. M. Morales, P. Karafiloglou, C. R. Landis, and F. Weinhold, Theoretical Chemistry Institute, University of Wisconsin, Madison, WI (2018).

[15] R. F. W. Bader, *Chem. Rev.* **1991**, *91*, 893 – 928.

[16] T. Lu, F. Chen, *J. Comput. Chem.* **2012**, *33*, 580 – 592.

[17] W. Humphrey, A. Dalke, K. Schulten, *J. Molec. Graphics* **1996**, *14*, 33 – 38

[18] CYLview, 1.0b; Legault, C. Y., Université de Sherbrooke, **2009** (http://www.cylview.org)
